# Molecular mechanisms and therapeutic strategies of cGAS-STING pathway in liver disease: the quest continues

**DOI:** 10.3389/fimmu.2025.1692365

**Published:** 2025-12-08

**Authors:** Yichen Fan, Zihao Dong, Yufeng Wu, Hao Wen

**Affiliations:** 1 State Key Laboratory of Pathogenesis, Prevention and Treatment of High Incidence Diseases in Central Asia, Clinical Medicine Institute, The First Affiliated Hospital of Xinjiang Medical University, Urumqi, Xinjiang, China; 2 The First Affiliated Hospital of Xinjiang Medical University, Urumqi, Xinjiang, China

**Keywords:** cGAS-STING, NAFLD, ALD, viral hepatitis, HIRI, C-DILI, HCC, liver cirrhosis

## Abstract

The cyclic GMP-AMP synthase–stimulator of interferon genes (cGAS-STING) pathway has emerged as a central regulator of liver homeostasis and pathology, governing innate immunity, inflammation, fibrogenesis, and tissue repair. Dysregulated cGAS-STING signaling, often driven by cytosolic DNA sensing, cellular stress, or cross-activation with other immune pathways, leads to excessive type I interferons and pro-inflammatory cytokine production, exacerbating liver injury. This aberrant activation is implicated in chronic liver inflammation, fibrosis, and carcinogenesis, highlighting its dualistic role in both protective and pathogenic processes. This systematic review synthesizes current evidence on the context-dependent roles of the cGAS-STING pathway across liver diseases, including non-alcoholic fatty liver disease (NAFLD), alcoholic liver disease (ALD), viral hepatitis, hepatocellular carcinoma (HCC), drug-induced liver injury (DILI), hepatic ischemia-reperfusion injury (HIRI), and parasitic infections. The cGAS-STING pathway exhibits dualistic functions in liver pathophysiology: while its activation promotes antiviral defense and tissue regeneration in acute injury, chronic hyperactivation drives inflammation, fibrosis, and oncogenesis. In NAFLD/ALD, metabolic stress and mitochondrial DNA leakage perpetuate STING-dependent inflammation, whereas in HCC, persistent signaling accelerates tumor progression and immune evasion. Similarly, in parasitic diseases or HIRI, cGAS-STING activation may enhance pathogen clearance or exacerbate tissue damage, depending on disease stage. Emerging therapeutic strategies, including STING inhibitors, agonists, and nano modulators, show promise in preclinical models but require context-specific optimization to balance beneficial immunity and pathologic outcomes. Understanding these context-dependent functions of cGAS-STING pathway provides critical insights for the development of targeted therapeutic strategies that may selectively modulate this pathway to treat diverse hepatic disorders.

## Introduction

The global burden of liver diseases continues to escalate, with viral hepatitis, fatty liver diseases, cirrhosis, and HCC presenting significant public health challenges worldwide (1). Current therapeutic strategies are limited by suboptimal durability of response, frequent adverse events, and poor patient tolerability. These challenges are particularly pronounced in conditions such as nonalcoholic steatohepatitis (NASH), advanced-stage liver cancer, and autoimmune liver diseases, for which effective clinical interventions are limited (2). There is an urgent need to identify novel therapeutic targets that address the fundamental mechanisms underlying these disorders (3). Consequently, research focus has transitioned from the macroscopic characterization of liver pathology to a more in-depth exploration of molecular pathways, aiming to uncover upstream regulatory nodes and convergent signaling axes that may provide new avenues for intervention. The elucidation of cytosolic DNA sensing mechanisms represents a significant breakthrough in modern immunology, providing critical insights into how cells precisely detect aberrant molecular patterns (4). Upon recognizing anomalous DNA signals—whether derived from viruses, bacteria, or released during cellular injury—cells can rapidly initiate innate immune responses, subsequently modulating adaptive immunity (5). Throughout evolution, cells have developed intricate and highly selective surveillance systems that convert such mislocalized DNA into potent immunostimulatory signals, exemplifying the sophisticated transition from physical danger detection to molecular immune activation. At the core of cytosolic DNA recognition is the cGAS-STING pathway. By detecting double-stranded DNA that is ectopic within the cytoplasm, cyclic GMP-AMP synthase (cGAS) catalyzes the production of the second messenger cyclic GMP-AMP (cGAMP), which in turn activates the adaptor protein STING. This activation triggers a robust immune cascade, including the induction of type I interferons (IFN-I) and pro-inflammatory cytokines (6). Beyond its pivotal role in innate immune defense, the cGAS-STING pathway also modulates antigen presentation and T cell activation, thereby serving as a crucial link between innate and adaptive immunity (7). The role of the cGAS-STING pathway axis in liver disease is not unidimensional; rather, it exhibits a classic “double-edged sword” effect (8). On one hand, by sensing pathogen- and damage-associated DNA, it effectively initiates immune protective mechanisms against viral infections, tumors, and metabolic injuries, thereby facilitating the clearance of pathogenic factors and suppressing uncontrolled cellular proliferation. On the other hand, excessive activation or dysregulation of the cGAS-STING pathway may trigger autoimmune responses, exacerbate chronic inflammation, and even promote tumor progression and fibrosis through mechanisms such as immune tolerance and T cell exhaustion. The cGAS-STING pathway exerts diverse regulatory functions across various cell types in the liver. In hepatocytes, it monitors mitochondrial DNA (mtDNA) release and viral infections; in Kupffer cells, it mediates inflammatory responses to necrotic cell debris; in hepatic stellate cells (HSCs), it modulates cellular activation and fibrogenesis; and in infiltrating T cells, it influences anti-tumor functionality, thereby orchestrating the immune response (9). This cellular heterogeneity underscores the context-dependent nature of cGAS-STING pathway in hepatic pathophysiology, where the balance between protective and pathogenic effects determines disease outcomes (8–10). This molecular regulatory network permeates multiple cell types and traverses multilayered pathological processes, positioning cGAS-STING as a pivotal signaling hub in hepatic disorders, intimately connecting upstream DNA pattern recognition with downstream histopathological alterations (11). Understanding and precisely modulating the activation thresholds and signal outputs of cGAS-STING across different hepatic cells enables intervention in liver disease progression at molecular, cellular, and tissue levels. This approach not only deepens our comprehension of hepatic pathomechanisms but also provides multidimensional entry points for the development of novel targeted therapeutic strategies. Building on these insights, our review provides a comprehensive synthesis of the literature linking the cGAS–STING pathway to a broad spectrum of liver diseases. Detailed methods, inclusion criteria, and the complete search strategy are described in the **Supplementary Materials**.

## cGAS-STING pathway: fundamental mechanisms and core functions

**Figure 1:**
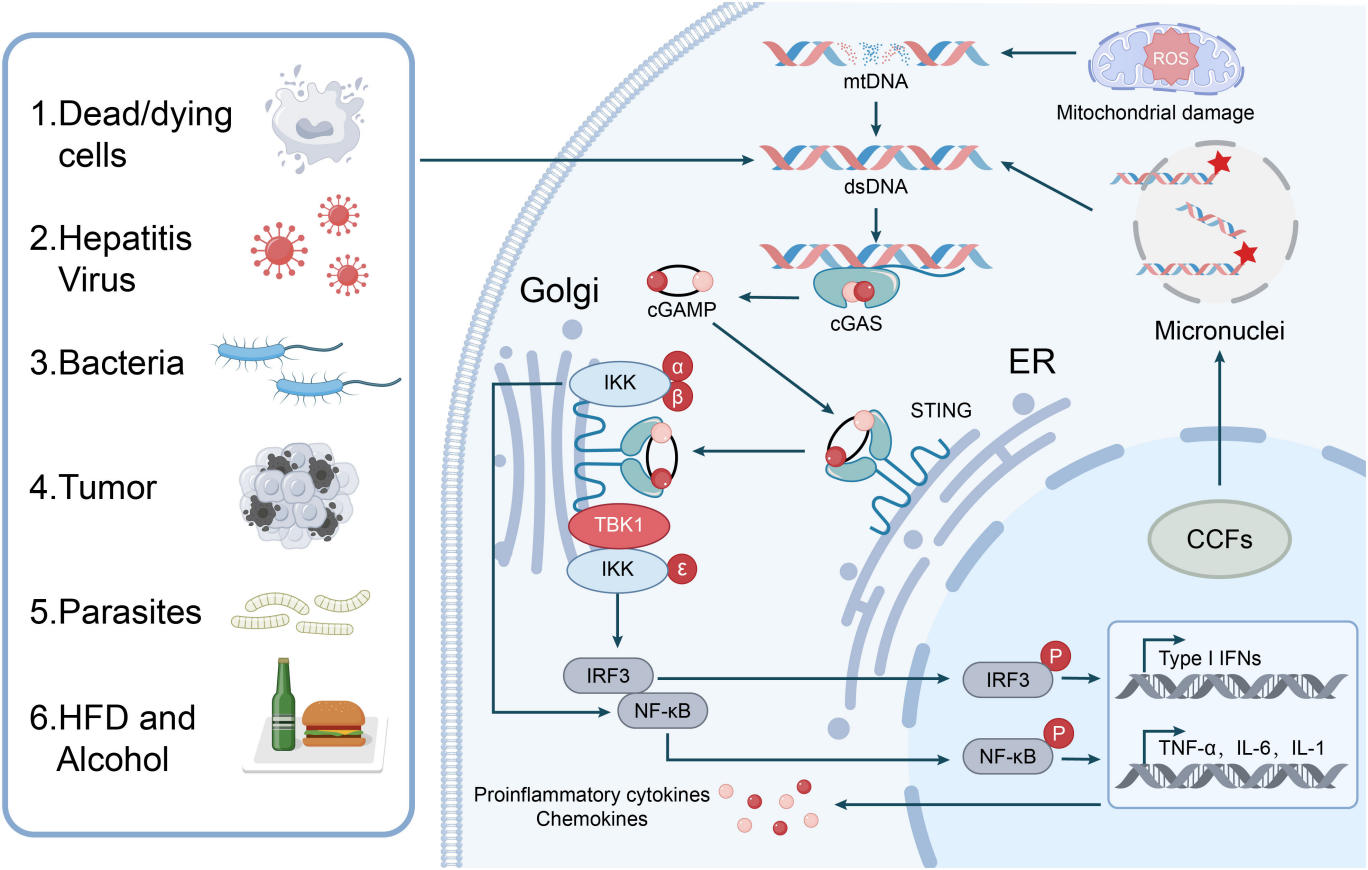
cGAS-STING signaling pathway activation in liver cells. The cytosolic DNA sensor cGAS is activated by various stimuli, including dead/dying cells, hepatitis viruses, bacteria, tumor cells, parasites, HFD and alcohol, leading to the detection of aberrant DNA such as mtDNA, dsDNA from the cytoplasm, and DNA from micronuclei. Mitochondrial damage, potentially induced by ROS, can release mtDNA. Upon DNA binding, cGAS catalyzes the production of cGAMP, which then binds to STING located in the ER. STING activation triggers downstream signaling cascades involving TBK1 and IKK complexes (IKKα/β/ϵ). These kinases phosphorylate and activate IRF3 and NF-κB. Activated IRF3 and NF-κB translocate to the nucleus to induce the expression of Type I IFNs and proinflammatory cytokines and chemokines (TNF-α, IL-6, IL-1, and other chemokines), contributing to CCFs.

The cGAS-STING pathway has recently attracted significant attention in liver disease research due to its crucial role in regulating immune responses (12–15). This signaling consists of the enzyme cGAS and the transmembrane protein STING, which together function to recognize cytosolic double-stranded DNA (dsDNA), a hallmark of cellular stress or infection (16, 17). Upon recognizing dsDNA, cGAS, a vital DNA sensor, activates downstream immune responses (18). As a structurally conserved member of the CDNtase superfamily, cGAS is widely present in both prokaryotic and eukaryotic organisms (19–21). Its catalytic domain is composed of N-terminal and C-terminal regions. The N-terminal region is primarily responsible for catalytic activity, while the C-terminal lobe comprises a helical bundle that contains a conserved zinc-ion-binding module, which mediates both DNA binding and cGAS dimerization (16, 22, 23). cGAS is exclusively activated by B-form dsDNA and does not possess the ability to recognize A-form dsDNA or RNA, exhibiting no sequence specificity (18). Furthermore, full activation of cGAS requires dsDNA of a minimum length of 20 base pairs, as shorter fragments induce only weak dimerization of cGAS (24). In 2013, James Chen and his team investigated a fundamental question concerning the mechanism by which the cGAS-STING pathway detects cytosolic dsDNA (22). Upon the detection of dsDNA, cGAS catalyzes the production of cGAMP, a second messenger that binds to and activates STING (22). Using biochemical purification and quantitative mass spectrometry, Chen’s team identified cGAMP as a novel signaling molecule characterized by a unique 2′-5′ phosphodiester bond, which distinguishes it from bacterial-derived cyclic dinucleotides such as c-di-GMP and c-di-AMP (20, 22, 25). Furthermore, they demonstrated that cGAMP binds to STING with greater affinity and activation potential than bacterial CDNs, underscoring its pivotal role in innate immune signaling (25). The importance of cGAS in this pathway was confirmed through studies utilizing cGAS-knockout mouse models, which exhibited a complete loss of cytosolic dsDNA sensing and immune activation, thereby highlighting the enzyme’s essential role (11). This translocation is critical for the downstream signaling cascade, ultimately leading to the activation of interferon regulatory factors (IRFs) and the induction of an antiviral state. STING, a 42 kDa transmembrane protein characterized by four transmembrane domains and a cytoplasmic C-terminal tail, is localized in the endoplasmic reticulum (ER) (26). Upon binding to the second messenger cGAMP, STING undergoes conformational changes and becomes activated. Once activated, STING translocates to perinuclear compartments, where it assembles into punctate structures and recruits TANK-binding kinase 1 (TBK1), thereby initiating a highly ordered phosphorylation cascade. Interferon regulatory factor 3 (IRF3) is a key target of this phosphorylation process. Following phosphorylation, IRF3 dimerizes and translocates to the nucleus, driving the production of IFN-I (27, 28). The induction of type I interferons mediated by the STING-TBK1-IRF3 pathway represents effect of STING activation. IFN-I transmit signals between cells through paracrine or autocrine mechanisms. Upon binding to cell surface receptors, IFN-I activates the JAK/STAT signaling pathway, which subsequently induces the expression of interferon-stimulated genes (ISGs), ultimately establishing an antiviral state that inhibits viral replication and spread (29). Furthermore, STING activation triggers downstream signaling events, including the activation of the IκB kinase (IKK) complex, leading to the phosphorylation and degradation of IκB proteins, thereby releasing NF-κB. Subsequently, NF-κB translocates to the nucleus to initiate the transcription of inflammatory cytokine genes, including interleukin-6 (IL-6) and tumor necrosis factor-α (TNF-α), thus promoting inflammatory responses (**Figure 1**) (30, 31). Notably, TBK1 exerts negative feedback regulation by recruiting and phosphorylating p62 (also known as sequestosome 1, SQSTM1). Phosphorylated p62 enhances its binding affinity to ubiquitinated STING, thereby facilitating STING degradation through autophagy, which limits the duration and intensity of cGAS-STING signaling to prevent excessive immune responses (32, 33). In summary, the activation of the cGAS-STING pathway triggers two principal downstream pathways: STING-TBK1-IRF3 and IKK-NF-κB. These pathways mediate the production of IFN-I and inflammatory cytokines, respectively, and collectively regulate immune and inflammatory responses. These intricate signaling networks play critical roles in the pathogenesis of various diseases, including viral hepatitis, liver fibrosis, and HCC. A comprehensive analysis of the cGAS-STING signaling pathway not only enhances our understanding of the molecular mechanisms underlying immune and inflammatory responses but also identifies significant targets and strategies for the development of innovative therapies for cancer, infectious diseases, and autoimmune disorders.

## Initial activation of cGAS-STING pathway in liver disease

The innate immune system functions as the body’s primary defense against pathogenic invasion, playing a crucial role in both pathogen recognition and the activation of adaptive immune responses (34). The aberrant presence of dsDNA, originating from either endogenous sources (such as damaged mitochondria, dying cells, DNA damage, and genomic instability) or exogenous sources (including parasites, bacteria, DNA viruses, and retroviruses), serves as a significant signal of liver injury or infection. These DNA signals are primarily detected by the cytosolic DNA sensor cGAS-STING pathway (13, 35). The mtDNA can be released into the cytoplasm when the integrity of the mitochondrial membrane is compromised by various stressors, such as oxidative stress, pathogen infection, and toxin exposure. Similarly, while microbial DNA is typically safeguarded within nucleoid-like structures, the disruption of microbial cellular barriers during infection can expose this DNA to cytosolic cGAS detection, subsequently activating the STING pathway and triggering immune responses (**Figure 1**) (36–38). Research indicates that exposure to microplastics, CCl_4_, and mitochondrial dysfunction can activate the cGAS-STING pathway, resulting in inflammation, fibrosis, and endothelial dysfunction. In HCC, the cGAS-STING pathway suppresses tumor progression by promoting immune cell infiltration and activation (39, 40). The mechanisms by which cellular DNA is sensed play diverse biological roles, opening new avenues for disease prevention and treatment. The therapeutic potential of modulating the cGAS-STING pathway has emerged as a promising strategy in clinical applications (41). Pharmaceutical development has primarily focused on STING activation, particularly to enhance cancer immunotherapy (42). Conversely, the development of inhibitors targeting cGAS or STING functions represents another therapeutic approach, particularly for treating complex inflammatory and autoimmune diseases (43, 44). To better understand the role of cGAS-STING signaling in various liver diseases, it is essential to investigate its disease-specific activation patterns, regulatory mechanisms, and downstream effects. This includes examining how different hepatic pathologies trigger pathway activation, how the signal is propagated within the liver microenvironment, and how its dysregulation contributes to disease progression or resolution. Accumulating evidence has highlighted the significance of the cGAS-STING pathway in diverse hepatic disorders, including NAFLD, ALD, viral hepatitis, HIRI, C-DILI, Liver Neoplasms, Liver Cirrhosis, and Parasitic Liver Disease (12, 13, 45). While the cGAS-STING pathway primarily functions to orchestrate innate immune responses through the production of IFN-I and proinflammatory cytokines, its activation can also trigger programmed cell death, suggesting a dual role in cellular fate. This dual functionality becomes particularly significant in liver pathology, where dysregulation of this innate immune pathway can either protect against or contribute to various hepatic diseases (8, 46). Excessive activation of the cGAS-STING pathway in NAFLD/ALD may lead to sustained inflammatory responses and tissue damage (47, 48). The cGAS-STING pathway, emerges as a pivotal target in cancer therapeutics due to its acute activation-induced antitumor immunity. However, emerging evidence underscores its dichotomous nature in oncogenesis – chronic activation paradoxically drives protumorigenic processes including sustained inflammation and immunosuppressive niche formation (49, 50).

## Non-alcoholic fatty liver disease and alcoholic liver disease

In recent years, studies have elucidated the pivotal role of the cGAS-STING pathway in the pathogenesis of NAFLD and ALD (**Table 1**, **Supplementary Table 1**). The transport of gut-derived endotoxin lipopolysaccharide (LPS) to the liver is a primary cause of ALD, as it triggers innate immune dysregulation and hepatic inflammation (76). Petrasek et al. (2013) first identified the role of IRF3 as a signaling molecule mediating the crosstalk between ER stress and hepatocyte apoptosis, demonstrating the critical function of STING in the phosphorylation of IRF3 within the ALD (70). Patel et al. (2018) showed that cGAS can induce alcohol-related IRF3 activation via the gap junction protein Cx32, revealing a link in the pathogenesis of ALD (56). Huang et al. (2022) revealed that abnormally elevated sterol regulatory element-binding protein cleavage-activating protein (SCAP) in macrophages activates the NF-κB signaling pathway, amplifying inflammatory responses in adipose tissue and the livers (61). Wang et al. (2022) demonstrated that STING functions as a mtDNA sensor in liver Kupffer cells, initiating STING signaling pathway-dependent inflammation that exacerbates hepatocyte apoptosis in a Gao binge ethanol model (71). To maintain mitochondrial homeostasis, dysfunctional mitochondria can be selectively eliminated through mitophagy and replenished via mitochondrial biogenesis, constituting an adaptive response to alcohol consumption (71). Ma et al. (2023) utilized liver samples from patients with alcoholic hepatitis and liver-specific DRP1 gene KO mice exhibiting impaired mitochondrial fission. As ALD progresses, sustained DRP1 deficiency may lead to mitochondrial maladaptation and impaired mitophagy, which intensifies innate immune dysregulation and exacerbates liver injury (60).

**Table 1:** Summary of studies on the effects of STING in NFALD and ALD.

Study	Disease	Agent	STING	Effects on liver disease	Ref.
Luo 2018	NAFLD/NASH	STINGgt/DMXAA or cGAMP	Inhibition/Activation	Reducing severe hepatic steatosis, inflammation, and fibrosis/Promote HSC activation	(51)
Qiao 2018	NAFLD	STING-IRF3 KO	Inhibition	Improving the glucose and lipid metabolism disorder of L-O2 cells induced by FFA.	(52)
Liu 2022	NAFLD/NASH	Aucubin	Inhibition	Inhibiting HFD-induced inflammation, alleviating the progression of NASH	(53)
Qi 2022	ALD	Curcumin	Inhibition	Inhibition of hepatocyte senescence, LC3B-II accumulation, and CCF formation in AFLD hepatocytes	(54)
Wang 2024	NAFLD/NASH	FE	Inhibition	Anti-inflammatory effects of flavonoid components and the scientific connotation of the Chinese medicine’s multi-component synergism	(55)
Patel 2018	ALD	Cx32	Activation	The role of gap junction–dependent communication	(56)
Luo 2023	NAFLD/NASH	LE	Inhibition	Reducing liver inflammation and fibrosis in NASH	(57)
Li 2022	NFALD/HCC/ALD/Hepatitis	Iron overload	Activation	Enhancement of TBK1, IRF-3, and NF-κB expression An important risk factor for HCC	(58)
cho 2018	NAFLD/NASH	Lipotoxicity	Activation	TBK1 activation induces phosphorylation and aggregation of p62	(59)
Ma 2023	ALD/AH	DRP1 KO	Activation	Dysfunctional innate immune response and impaired mitophagy	(60)
Huang 2022	NAFLD	SCAP	Activation	Promoting inflammation and metabolic disorders leading to NAFLD	(61)
Luo 2022	NAFLD/NASH	mEV	Activation	Increasing hepatocyte inflammation and HSC activation.	(62)
Yang 2023	NASH	YAP KO	Inhibition	Increasing PLIN2 expression and reduces LD degradation in lysosomes	(63)
Ribeiro 2023	NASH	cGAS KO	Inhibition	Promoting liver injury, steatosis and inflammation in NASH Impairs intestinal barrier, increases gut permeability	(64)
Li 2020	NFALD	Remdesivir	Inhibition	Reducing lipid accumulation, inflammation and metabolic syndrome	(65)
Donne 2022	NFALD	RS	Activation	Slow replication fork progression and the activation of an S-phase checkpoint (ATR signaling)	(66)
Lin 2023	NFALD/NASH	RNF13	Inhibition	Alleviation of insulin resistance, steatosis, inflammation, cell injury, and fibrosis.	(67)
Miao 2023	NFALD/NASH	SelW	Activation	Reprograms glycolysis, activates HIF‑1α, and drives macrophage reprogramming.	(68)
Wang 2020	NFALD/NASH	MoMFs	Activation	Promoting liver inflammation and fibrosis progression	(69)
Petrasek 2013	ALD	Ethanol	Activation	Initial discovery of IRF3’s activation and pro-apoptotic function	(70)
Wang 2022	ALD	DMXAA	Activation	Promoting hepatocyte apoptosis and hepatic inflammatory infiltration and liver injury	(71)
Yu 2019	NFALD/NASH	KCs	Activation	Acts as a mtDNA sensor, inducing NF-κB-dependent inflammation	(72)
Zhang 2022	NFALD	STING gt	Inhibition	Alleviating steatohepatitis and liver injury and affecting the biodiversity of intestinal microbiota	(73)
Siao 2022	NFALD/NASH	Tmem173 gt	Inhibition	No protective effect against hepatic steatosis or injury Systemic insulin resistance is significantly reduced	(74)
Cao 2022	NFALD	LGZG	Inhibition	Reducing insulin resistance, inflammation and lipid deposition	(75)

**Figure 2:**
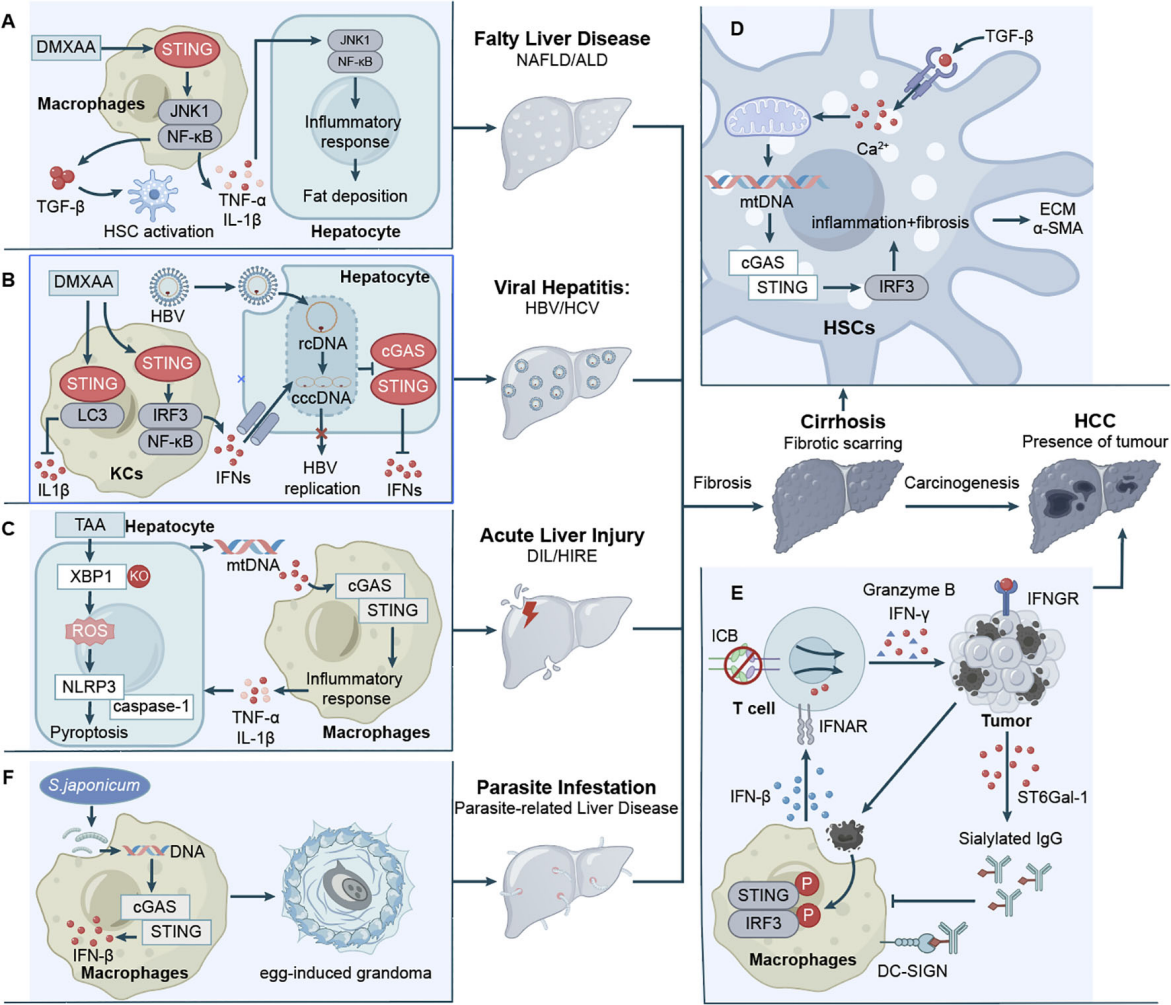
cGAS-STING signaling pathway activation in liver cells. This figure illustrates how the cGAS–STING pathway contributes to different liver injury contexts and disease progression. **(A)** In NAFLD, macrophage STING activation drives TGF-β release and HSC activation, promoting fibrosis and hepatic fat deposition. **(B)** HBV cccDNA suppresses innate DNA sensing in KCs, whereas pharmacologic STING activation restores antiviral signaling through IL-1β and LC3 induction. **(C)** In ALI, hepatocyte mitochondrial stress triggers mtDNA release, which activates cGAS–STING and NLRP3 inflammasome signaling in macrophages, amplifying inflammation. **(D)** In fibrosis and cirrhosis, HSC-derived mtDNA further stimulates the cGAS–STING–IRF3 axis, reinforcing HSC activation and extracellular matrix deposition that may progress to carcinogenesis. **(E)** In HCC, STING signaling in macrophages supports IFN-β production and antitumor T-cell responses, enhanced by DC-SIGN-mediated IgG desialylation and immune checkpoint blockade. **(F)** In schistosomiasis, macrophage DNA release activates cGAS–STING, inducing IFN-β production and promoting egg-induced granuloma formation and liver fibrosis.

Obesity often leads to hepatic steatosis, which can cause lipotoxic damage to hepatocytes, triggering NASH (77). Cho et al. (2018) showed that activation of TBK1 phosphorylates and aggregates p62, promoting ubiquitinated protein inclusions under lipotoxic stress (59). Qiao et al. (2017) reported elevated STING and phosphorylated IRF3 expression in obese mice livers and fatty acid-treated L-O2 cells, with STING or IRF3 knockdown improving glucose and lipid metabolism (52). Wang et al. investigated STING expression predominantly in liver macrophages, including monocyte-derived macrophages, and associated these findings with clinical data from 98 patients with non-alcoholic fatty liver disease (69). Siao et al. (2022) noted that STING deficiency does not affect steatosis, inflammation, or fibrosis but worsens insulin resistance in long-term models, possibly due to dietary differences (e.g., high-fructose vs. high-fat diets) in STING pathway activation (74). This apparent contradiction with earlier findings, such as Luo et al. (2018), where STING knockout ameliorates fibrosis and macrophage inflammation in high-fat diet (HFD)-induced models, underscores the context-dependent nature of STING signaling in NAFLD (**Figure 2A**) (51). The role of STING in NAFLD/NASH is phase-specific and trigger-dependent. Under acute or HFD-induced lipotoxic stress, STING activation amplifies NF-κB–mediated inflammation and fibrosis through mitochondrial DNA sensing in Kupffer cells (Yu et al., 2019), whereas its deficiency mitigates cytokine storms (TNF-α, IL-6) and thus exerts protective effects (72). Gut microbiota composition also appears to influence this process, Zhang et al. (2022) showed that STING-deficient mice exhibit reduced hepatic lipid accumulation and inflammation (73). Mechanistically, Yang et al. (2023) demonstrated that macrophage STING deletion decreases TBK1–LAST1-mediated inhibitory phosphorylation of YAP, thereby enhancing nuclear YAP activity to promote lipid metabolism, fatty acid oxidation, and lipophagy-dependent degradation of the lipid droplet protein PLIN2, collectively alleviating steatosis and oxidative stress (63). In contrast, Ribeiro et al. (2023) found that cGAS or STING deficiency exacerbates HF-HC-HSD–induced NASH, increasing steatosis, inflammation, and injury—likely due to impaired gut barrier integrity (64). Consistently, Luo et al. (2023) demonstrated that depletion of hepatic Vsig4^+^ macrophages together with accumulation of bacterial DNA in hepatocytes and stellate cells drives NAFLD/NASH progression by reflecting gut–liver barrier dysfunction and microbial translocation (62). The cGAS-STING pathway in NAFLD/ALD reveals that it functions as a common inflammatory amplifier, yet is engaged by disease-specific triggers and elicits distinct pathological consequences—exemplified by the dual-edged role of STING deficiency, which is protective against acute inflammation but potentially detrimental to chronic metabolic adaptation.

Therapeutic exploration of the cGAS-STING pathway has achieved some progress. Li et al. (2020) found that remdesivir (RDV) inhibits STING signaling, reducing lipid metabolism abnormalities and inflammation in NAFLD (65). Li et al. (2022) utilized ferric ammonium citrate to investigate iron metabolism dysregulation contributes to the progression of chronic liver diseases (58). Cao et al. (2022) demonstrated that compounds from Linggui Zhugan Decoction (cinnamaldehyde, atractylenolide II, glycyrrhizin) dose-dependently suppress STING-induced inflammation (75). Liu et al. (2022) showed that aucubin mitigates NASH progression in HFD-fed mice by upregulating miR-181a-5p, inhibiting STING/NF-κB pathway, and reducing inflammation (53). Lin et al. (2023) identified RNF13 as a stabilizer of TRIM29, promoting STING degradation and offering an innate immunity-related target for NAFLD (67). Luo et al. (2023) showed that licorice extract inhibits STING oligomerization, alleviating inflammation and fibrosis in MCD diet-induced NASH models (57). Miao et al. (2023) revealed that SelW interacts with PKM2 to regulate glycolysis and lipid metabolism, triggering HIF-1α-mediated apoptosis, ROS production, and NLRP3 inflammasome activation, with mtDNA leakage driving macrophage transformation via cGAS-STING, promoting NAFLD, fibrosis, and HCC (68). Wang et al. (2024) found that Epimedium flavonoid (EF) suppresses cGAS-STING activation, reducing IFN-I and pro-inflammatory cytokine expression (55). For ALD, Qi et al. (2022) reported that curcumol inhibits cGAS-STING activation, possibly by maintaining nuclear membrane integrity via LC3B-lamin B1 disruption, reducing inflammatory cytokine release (54). The cGAS-STING pathway in NAFLD/ALD reveals that it functions as a common inflammatory amplifier, yet is engaged by disease-specific triggers and elicits distinct pathological consequences. The shared mechanisms are rooted in the fundamentals of hepatic injury: mitochondrial damage and gut-derived signals lead to cytosolic DNA accumulation, which is primarily sensed by cGAS-STING in macrophages and Kupffer cells, driving inflammation mediated by IRF3 and NF-κB. However, the divergence in activation mechanisms defines the unique pathology of each disease. In ALD, the pathway is uniquely activated by alcohol metabolism, engaging specific mechanisms such as Cx32 gap junction-mediated propagation and DRP1-dependent mitophagy impairment. Conversely, in NAFLD, lipotoxicity engages unique regulatory axes, including the STING-YAP pathway that governs lipid autophagy and TBK1-p62-mediated protein aggregation. These distinct molecular networks naturally inform different therapeutic targeting strategies. This synthesis underscores that while the cGAS-STING pathway represents a convergent node in sterile inflammation, its context-dependent regulation dictates disease outcomes.

### Viral hepatitis

The cGAS-STING pathway has garnered significant attention for its role in hepatitis, particularly in HBV infection (**Table 2**; **Supplementary Table 2**). The cellular innate immune system is essential for recognizing pathogen infections and plays a crucial role in the host’s defense against HBV (98). Research conducted by Guo et al. (2015) and Liu et al. (2015) has demonstrated that the STING agonist DMXAA activates the STING pathway in macrophages, leading to the upregulation of IFI16/IFI204 expression and the promotion of inhibitory histone modifications on covalently closed circular DNA (cccDNA), which in turn suppresses HBV replication (94). Additionally, it has been shown that HBV polymerase directly interacts with STING, inhibiting its K63-linked ubiquitination and disrupting the production of IFN-I (85). Dansako et al. (2016) and He et al. (2016) identified cGAS as a crucial host factor for recognizing DNA viral genomes, demonstrating that HBV infection induces ISG56 expression through the cGAS-STING pathway. This finding emphasizes the vital role of cGAS in activating IFN-β expression and inhibiting HBV replication (89, 97). Conversely, Thomsen et al. (2016) reported that hepatocytes exhibit low STING expression and diminished responsiveness to exogenous DNA, leading to a reduced antiviral immune response during adenovirus (AdV)-mediated HBV infection (91). Lauterbach-Rivière et al. (2020) proposed that HBV employs a passive immune evasion strategy by generating non-immunostimulatory RNA and evading DNA sensing through cGAS-STING, rather than actively suppressing the DNA-sensing pathway (84). In contrast, Chen et al. (2022) found that HBx, an HBV-specific protein, actively inhibits cGAS expression by promoting its ubiquitination and autophagic degradation, thereby evading the cGAS-STING-mediated innate immune response and facilitating viral persistence (83). Zhao et al. (2023) further revealed that HBV achieves innate immune evasion through mechanisms involving the acetylation of histone acetyltransferase 1 (HAT1), histone H4 lysine 5 (H4K5), H4K12, and microRNA-181a-5p, or by modulating the karyopherin alpha 2 (KPNA2)/cGAS-STING/IFN-I signaling axis (81). Li et al. (2022) demonstrated that STING activation induces autophagy flux, suppressing macrophage inflammasome activation, which in turn inhibits cccDNA-driven transcription, HBV replication in hepatocytes, and the progression of HBV-associated liver fibrosis (**Figure 2B**) (99). Zheng et al. (2021) provided novel insights by demonstrating that the STING axis disrupts NK cell function in HBeAg-negative CHB infections (82). These findings illuminate potential therapeutic strategies. Guo et al. (2017) reported that pharmacological activation of the STING pathway in macrophages and hepatocytes effectively induces a host innate immune response, suppressing HBV replication (79). Similarly, Polidarova et al. (2023) showed that STING agonists induce cytokine-mediated HBV suppression in primary human hepatocytes and non-parenchymal liver cells (NPCs) (95). Choi et al. (2020) found that GV1001 enhances the release of oxidized DNA from mitochondrial stress into the cytosol, eliciting IFN-I-dependent anti-HBV effects through the STING-IRF3 axis (78). Lin et al. (2021) demonstrated that Mn²^+^ promotes type I IFN production during AAV-HBV infection and HBsAg immunization, suggesting its potential as a vaccine adjuvant (92). Shu et al. (2023) reported that RVX-208 inhibits HBV by regulating cholesterol metabolism and activating the cGAS-STING pathway (93). Rodriguez-Garcia et al. (2021) described the bacterial diguanylate cyclase AdrA, which induces IFN-β and ISG54 via STING (80). Zhao et al. (2023) reported that Schisandra chinensis (SC) enhances cGAS-STING pathway activation to inhibit HBV replication (100), while Wu et al. (2024) identified its component luteolin as a potent antiviral agent that synergistically suppresses HBV replication (96).

**Table 2:** Summary of studies on the effects of STING in HBV and HCV infections.

Study	Disease	Agent	STING	Effects on liver disease	Ref.
Choi 2020	HBV	GV1001	Activation	A safe peptide, shows potential synergy with NA drugs against HBV	(78)
Guo 2017	HBV	cGAMP/DMXAA	Activation	Inducing a JAK1-dependent antiviral response and inhibiting cccDNA transcription.	(79)
Rodriguez 2021	HBV	AdrA	Activation	An alternative adjuvant for vaccines or immunomodulatory candidates	(80)
Zhao 2023	HBV	HAT1	Inhibition	Epigenetic regulation of cGAS-STING enables viral immune evasion	(81)
Zheng 2021	HBV	HBsAg	Inhibition	Providing a new mechanism of NK cell disability in CHB infection	(82)
Chen 2022	HBV	HBx	Inhibition	Targeting cGAS via autophagy and ubiquitination enables evasion of innate immune responses	(83)
Lauterbach 2020	HBV	HBV RNA	No effect	The first time that HBV RNAs are not immunostimulatory	(84)
Liu 2015	HBV	HBV Pol	Inhibition	Revealing a novel mechanism by which HBV antagonizes the innate DNA sensing pathway	(85)
Ding 2013	HCV	HCV NS4B	Inhibition	Revealing molecular mechanisms of HCV infection persistence	(86)
Yi 2016	HCV	HCV 2a NS4B	Inhibition	Conferring resistance to cGAMP in HCV genotype 2a JFH1	(87)
Nitta 2013	HCV	HCV NS4B	Inhibition	Inhibition of RIG-I-mediated IFN-β production signaling	(88)
He 2016	HBV	cGAMP	Activation	Non-canonical cGAMP may inhibit HBV replication	(89)
Ono 2014	HCV	rBV-GFP	Activation	Baculovirus-induced innate immune response attenuates transgene expression	(90)
Thomsen 2016	HBV	AdV-HBV	Inhibition	The innate DNA-sensing pathway is not operative in hepatocytes due to lack of STING expression	(91)
Lin 2021	HBV	Mn2+	Activation	Enhancing the immune response to HBsAg immunization by functioning as an rHBV vaccine adjuvant	(92)
Shu 2023	HBV	RVX-208	Activation	Enhancing antiviral interferons, cytokines, and chemokines	(93)
Zhao 2023	HBV	SC	Activation	Reducing levels of HBeAg, HBcAg, HBsAg and HBV DNA	(100)
Guo 2015	HBV	DMXAA	Activation	IFN-dominant cytokine induction for HBV suppression	(94)
Li 2022	HBV	DMXAA	Activation	Epigenetic modifications lead to cccDNA functional inhibition, and enhanced autophagic flux suppresses the macrophage inflammasome.	(99)
Polidarova 2023	HBV	Various agonists	Activation	A cytokine-mediated anti-HBV response in NPCs	(95)
Wu 2024	HBV	SC+Lut	Activation	Significant inhibition of *in vivo* HBV DNA replication	(96)
Dansako 2016	HBV	p-dGdC/p-dAdT	Activation	cGAS: Host factor potentially modulating HBV infection sensitivity	(97)

HCV evades host immune responses through multiple complex mechanisms. Studies have shown that the NS4B protein of HCV directly interacts with key molecules in the RIG-I-like receptor signaling pathway, such as STING and MAVS, thereby disrupting their normal functions. The work of Ding et al. (2013) and Nitta et al. (2013) further confirmed these interactions, demonstrating that the NS4B protein suppresses interferon production and facilitates immune evasion by the virus. This allows HCV to persist in the host and contributes to the establishment of chronic infection (86, 88). Yi et al. (2016) demonstrated that the STING agonist cGAMP exerts potent antiviral effects against HCV. They also found that transient expression of the genotype 2a NS4B protein markedly inhibits STING-mediated activation of the IFN-β reporter gene expression, with the transmembrane domains of 2a NS4B being primarily responsible for the reduced sensitivity to cGAMP (87). Furthermore, Ono et al. (2014) proposed an innovative therapeutic strategy, showing that recombinant baculovirus can selectively induce apoptosis in cells lacking STING or other key antiviral signaling components, thereby eliminating HCV-infected cells. This provides a novel approach for HCV therapy (90). When considered alongside HBV, certain shared cGAS–STING–related mechanisms become apparent—such as macrophage-driven STING activation, IFI16-mediated sensing of viral genomes, and IRF3-dependent transcriptional responses. However, the viruses employ different evasion tactics. HBV primarily disrupts cGAS stability, STING ubiquitination, and nucleic acid immunogenicity, whereas HCV mainly targets the STING–MAVS interactions through NS4B. These differences naturally extend to therapeutic approaches: HBV research focuses on STING agonists that suppress cccDNA transcription, while HCV studies explore cGAMP treatment and NS4B-specific interventions. In conclusion, while the cGAS-STING pathway serves as a critical innate immune sensor against HBV, the virus has developed various evasion strategies, underscoring the complex interplay between viral genotype, liver-specific cellular responses, and STING pathway. Understanding these pathogen-specific signatures of cGAS–STING modulation will be critical for designing disease-tailored antiviral strategies.

### Chemical and drug induced liver injury

The cGAS-STING pathway plays a pivotal role in various forms of C-DILI, providing a foundation for potential therapeutic strategies (**Table 3**; **Supplementary Table 3**). Current evidence suggests that activation of the cGAS-STING pathway in DILI may be mediated by a variety of cellular stress responses and metabolic disturbances (131). Mitochondrial dysfunction emerges as a critical mechanism, as Zhong et al. (2022) elucidated that mtDNA leakage from senescent macrophages can trigger cGAS-STING pathway (102). Consistently, Liu et al. (2022) demonstrated that X-box binding protein 1 (XBP1) deficiency promotes mtDNA release and subsequent macrophage inflammation by suppressing mitophagy and fostering ROS/NLRP3/caspase-1/GSDMD-mediated hepatocyte pyroptosis (**Figure 2C**) (108). Furthermore, Saimaier et al. (2024) reported that trace element dyshomeostasis, specifically manganese overload, activates the cGAS-STING pathway, thereby mediating inflammation and exacerbating liver injury in a murine model of autoimmune hepatitis (AIH) (106). Zhao et al. (2024) observed that STING KO reduces hepatic iron accumulation, suggesting a key role for iron metabolism and its dysregulation in AIH pathogenesis (107). Yang et al. (2023), using PTEN gene knockout models of acute liver injury (AILI), discovered that NICD and NRF2 signaling crosstalk modulates and ultimately suppresses STING-TBK1 signaling, thereby mitigating acetaminophen (APAP)-induced hepatic inflammation (105). In recent years, targeting the cGAS-STING pathway for therapeutic inhibition has emerged as a promising strategy for managing drug/chemical-induced liver injury. Shaoyao Gancao Decoction (SGD) inhibits the liver cGAS-STING signaling pathway, thereby reducing HSCs activation and ultimately delaying the progression of AIH-related liver fibrosis (132). These findings align with broader observations in AILI models, where pathway modulation influences outcomes across etiologies. Liu et al. (2023) substantiated that L-Exosomes (L-Exo) can suppress macrophage STING signaling by enhancing mitophagy and preventing the cytoplasmic release of mtDNA, ultimately attenuating septic liver injury (101). Shen et al. (2022) and Li et al. (2023) demonstrated that Emodin and Ginsenoside Rd, respectively, can protect liver function by inhibiting the cGAS-STING pathway, thereby significantly alleviating APAP- and CCl_4_-induced acute liver injury in mice (103, 104).

**Table 3:** Summary of studies on the effects of STING in other liver diseases.

Study	Disease	Agent	STING	Effects on liver disease	Ref.
Liu 2023	Septic liver injury	L-Exo	Inhibition	mtDNA release inhibition, attenuates septic liver injury by enhancing ATG2B-mediated mitophagy	(101)
Zhong 2022	Liver sterile inflammation	In aged macrophages	Activation	Potential new therapeutic target for sterile inflammatory liver injury in elderly patients	(102)
Shen 2022	Injury (APAP)	Emodin	Inhibition	Hepatoprotection via inflammation and apoptosis reduction	(103)
Li 2023	Injury (CCl4)	Ginsenoside Rd	Inhibition	Reducing ferroptosis and protects against acute liver injury	(104)
Yang 2023	Injury (APAP)	PTEN	Inhibition	Therapeutic potential for managing sterile liver inflammation	(105)
Saimaier 2024	AIH	MnCl2	Activation	Accumulation promotes inflammation, leading to severe liver injury	(106)
Zhao 2024	AIH	Iron Overload	Activation	Cause liver damage and oxidative stress	(107)
Liu 2022	ALI	XBP1 Deficiency	Activation	Regulating hepatocyte pyroptosis and macrophage signaling activation	(108)
Zhong 2020	HIRI	Aging	Activation	Aggravating hepatocellular injury and intrahepatic inflammation	(109)
Jiao 2022	HIRI	C-178/H-151	Inhibition	Activating pro-inflammatory macrophages rely on glycolysis for rapid, short-term energy	(110)
Kong 2024	HIRI	Sirt3 Deficiency	Activation	Promotes p65 nuclear translocation and thereby upregulates cGAS transcription	(111)
Shen 2020	HIRI	miR-24-3p	Inhibition	Protecting against apoptosis and inflammation in liver	(112)
Zhan 2022	HIRI	TXNIP	Activation	Potential mechanisms of liver inflammation in BMDMs	(113)
Peng 2024	HIRI	PPM1G reduction	Activation	Enhancing M1 macrophage polarization, exacerbates HIRI	(114)
Wu 2022	HIRI	In KCs	Activation	Calcium influx promotion, macrophage caspase 1-GSDMD activation, potential NLRP3 inflammasome involvement	(115)
Shen 2022	Liver Cirrhosis	Micro-PS	Activation	Inducing nucleus damage and micronucleus formation	(116)
Luo 2023	Liver Cirrhosis	In LSECs	Activation	Promotion of liver fibrosis and sinusoidal microthrombosis formation, leading to elevated portal pressure	(117)
Iracheta 2016	Liver Cirrhosis	CCL4	Activation	Excessive IRF3-mediated apoptosis can cause secondary necrosis	(118)
Wu 2024	Liver Cirrhosis	IRF KO	Inhibition	IRF3-RB axis inhibits liver fibrosis by promoting cellular senescence	(119)
Shan 2023	Liver Cirrhosis	MitoQ	Inhibition	LonP1 activation and TDP-43 degradation improve mitochondrial function and inhibit inflammatory response	(120)
Sun 2023	Liver Cirrhosis	Oroxylin A	Activation	Regulation of HSC ferritinophagy and senescence	(121)
Zhao 2023	Liver Cirrhosis	Oroxylin A	Activation	Promoting HSC senescence, contributing to anti-fibrotic effects	(122)
Wang 2022	Liver Cirrhosis	SR-717	Activation	Inhibiting YAP, reduces pathological angiogenesis and organ fibrosis	(123)
Gu 2024	Liver Cirrhosis	Mn2+	Activation	Promoting HSC senescence, enhances NK cell activation	(124)
Wu 2023	Liver Cirrhosis	Parkin	Inhibition	Ubiquitinates VDAC1 at K53, disrupts VDAC1 oligomerization	(125)
Wang 2022	Liver Cirrhosis	XBP1 Deficiency	Activation	BNIP3-mediated mitophagy inhibition exacerbates liver fibrosis.	(126)
Xiao 2023	Liver Cirrhosis	NLRP3 Deficiency	Inhibition	Hepatic metabolic reprogramming attenuation for fibrosis amelioration	(127)
Arumugam 2023	Liver Cirrhosis	TGF-β	Activation	TGF-β–mediated transdifferentiation of HSCs drives liver fibrosis.	(128)
Liang 2022	Parasitic liver disease	STING KO	Inhibition	Reduction of egg granuloma formation without affecting liver fibrosis	(129)
Souza 2020	Parasitic liver disease	STING KO	Inhibition	Anti-infective: Neutrophil frequency and inflammatory profile of the gut microbiota	(130)

### Hepatic ischemia-reperfusion injury

Multiple studies have elucidated the pivotal role of STING in HIRI (**Table 3**; **Supplementary Table 4**). Macrophages play a complex yet pivotal role in the pathophysiology of HIRI, acting as both key mediators of inflammatory responses and participants in tissue repair (133). Zhong et al. (2020) elucidated the critical role of the STING-NLRP3 axis in HIRI in aged mice. Using BMDMs, they demonstrated that STING KO mitigated the detrimental effects of hepatic injury and intrahepatic inflammation (109). Jiao et al. (2022) indicated that STING may attenuate BMDM hyperactivation by suppressing hypoxia-inducible factor-1α (HIF-1α) and enhancing the activity of phosphorylated AMP-activated protein kinase (p-AMPK), suggesting a potential protective role against liver ischemia-reperfusion injury (110). Zhan et al. (2022) explored the function of thioredoxin-interacting protein (TXNIP) in BMDMs and primary hepatocytes, revealing its role in reducing hepatic inflammation and apoptosis/necrosis by modulating the CYLD-NRF2-OASL1 axis (113). Wu et al. (2022) reported that increased intracellular calcium in KCs activates the caspase 1-GSDMD pathway, potentially contributing to NLRP3 inflammasome activation and thereby exacerbating HIRI (115). Peng et al. (2024), utilizing the RAW264.7 macrophage cell line, discovered that decreased expression of PPM1G leads to STING pathway activation, promoting inflammation and M1 macrophage polarization, and consequently worsening liver injury (114). Kong et al. (2024), using the AML-12 hepatocyte cell line, revealed that Sirt3 deficiency results in p65 nuclear translocation, thereby amplifying cGAS transcription and hepatocyte damage (111). Shen et al. (2020) used bioinformatics analysis and experimental validation to demonstrate that miR-24-3p attenuates I/R injury by downregulating STING expression. This downregulation reduced STING and its downstream effector p-IRF3, decreased inflammatory cytokine release, and subsequently mitigated liver dysfunction and apoptosis (112). While STING predominantly acts as a central driver of pathological inflammation in macrophages and hepatocytes, evidence also suggests it may exert protective effects in certain settings, highlighting the intricate balance of this pathway in sterile injury.

### Liver cirrhosis

Accumulating evidence underscores the pivotal yet complex role of the cGAS-STING pathway in liver fibrosis, primarily through two interconnected mechanisms: the direct activation of HSCs and the amplification of immune-driven profibrotic responses (**Table 3**; **Supplementary Table 5**). The activation of quiescent HSCs into collagen-producing myofibroblasts is a central event in fibrosis (134), and the cGAS-STING pathway serves as a direct sensor for this process. A study by Arumugam et al. (2023) revealed that TGF-β signaling induces mtDNA release as a physiological mechanism that activates HSCs via the cGAS-STING pathway (**Figure 2D**) (128). Targeting activated HSCs directly is a promising strategy, as demonstrated by Gu et al. (2024), who developed an albumin-mediated manganese ion delivery system to specifically target activated HSCs for potent anti-fibrotic effects (124).

Beyond direct HSC activation, the cGAS-STING pathway potently exacerbates fibrosis by driving a pro-inflammatory microenvironment. Cellular stress and exogenous insults—such as microplastics and CCl_4_—induce nuclear and mitochondrial DNA damage; the release of double-stranded DNA subsequently activates cGAS–STING signaling (Shen et al. 2022; Shan et al. 2023) (116, 120). This immune axis involves multiple cell types: in hepatocytes, Xiao et al. (2023) identified that the STING-WDR5/DOT1L/IRF3-NLRP3 signaling axis potentiates pyroptosis and inflammation (127), while ER stress can trigger STING-IRF3-mediated hepatocyte apoptosis (Iracheta-Vellve et al., 2016) (118). In macrophages, Wang et al. (2022) also proposed that XBP1 regulates the cGAS/STING/NLRP3 inflammasome activation via BNIP3-mediated mitophagy, and its inhibition with Toyocamycin ameliorates fibrosis in mice (126). Furthermore, Luo et al. (2023) demonstrated that cGAS-STING activation in liver sinusoidal endothelial cells (LSECs) exacerbates intrahepatic inflammation and promotes microthrombosis, contributing to a profibrotic niche (117). The interplay between these direct and immune-mediated mechanisms is governed by intricate regulatory networks. For instance, IRF3, a key downstream transcription factor of STING, can interact with the retinoblastoma protein (RB) to inhibit CDK4/6-mediated hyperphosphorylation, and its conditional deletion in hematopoietic cells exacerbates fibrosis, underscoring its cell-specific protective role (Wu et al., 2024) (119). Conversely, therapeutic inhibition of downstream effectors like YAP, which is upregulated upon cGAS deletion, can suppress pathological angiogenesis and alleviate fibrosis (Wang et al., 2022) (123). Wu et al. (2023) found that E3 ubiquitin ligase Parkin ubiquitinates voltage-dependent anion channel 1 (VDAC1) at specific sites, preventing liver fibrosis by disrupting its oligomerization and mtDNA release (125). Additionally, Sun et al. (2023) and Zhao et al. (2023) have shown that natural compounds such as Oroxylin A have been shown to induce HSC ferroptosis and senescence by modulating the cGAS-STING pathway, offering alternative therapeutic avenues (121, 122). In summary, the cGAS-STING pathway orchestrates liver fibrosis through a dual-pronged mechanism: it directly transduces profibrotic signals in HSCs and simultaneously amplifies a damaging inflammatory cascade across hepatocytes, immune cells, and endothelial cells. A comprehensive understanding of this cell-specific duality is essential for developing precise and effective anti-fibrotic therapies.

### Hepatocellular carcinoma

In recent years, multiple studies have elucidated the pivotal role of the STING pathway in HCC, particularly in modulating antitumor immunity (**Table 4**; **Supplementary Table 6**). In the pathogenesis of HCC, cGAS functions as a pivotal sentinel that monitors genomic instability, adept at detecting both exogenous pathogen-derived DNA and endogenous DNA damage (160, 161). Genomic instability triggers pro-inflammatory signaling cascades and enhances tumor immunogenicity, thereby increasing the susceptibility of malignant cells to immune surveillance (162, 163). DNA damage in preneoplastic hepatocytes, which is induced by metabolic stress or viral hepatitis cofactors, results in the formation of micronuclei (164). The rupture of these micronuclei exposes fragmented DNA to cytoplasmic cGAS, triggering a dual response. The production of type I interferons and HCC-relevant chemokines, and the activation of the senescence-associated secretory phenotype (SASP) (165, 166). This activation drives competing biological processes in the cirrhotic liver microenvironment, promoting pro-inflammatory remodeling while simultaneously enhancing immune surveillance of transformed hepatocytes. Takahashi et al. (2018) demonstrated that the blockage of the cGAS/STING pathway prevented the SASP in senescent HSCs and reduced the incidence of obesity-associated HCC in mice (159). Zhang et al. (2021) revealed that low STING expression in HCC patients significantly correlates with larger tumor volumes, elevated serum alpha-fetoprotein (AFP) levels, and reduced infiltration of CD8+ T cells. These patients exhibited poorer overall and disease-free survival (138). Recent research by Xu et al. (2024) demonstrated that inhibition of eEF2K activates the cGAS-STING pathway, thereby enhancing NK cell proliferation while suppressing apoptosis. When combined with anti-PD-1 treatments, this intervention synergistically enhances NK cell infiltration and cytotoxic activity. This enhancement is characterized by an upregulation of NKG2D expression, suppression of NKG2A signaling, and increased secretion of granzyme B, TNF-α, and IFN-γ (140). Su et al. (2023) demonstrated that the deficiency of TAK1 in hepatocytes initiates oxidative stress and leads to ferritin deposition. This accumulation of ferritin results in the production of 8-OHdG, a marker of DNA oxidation, which subsequently activates the cGAS-STING pathway in macrophages. This activation ultimately contributes to liver injury, fibrosis, and tumorigenesis (155). Thomsen et al. (2019) demonstrated that treatment with a cyclic dinucleotide (CDN) STING agonist effectively inhibits tumor growth by promoting cell death, autophagy, and interferon responses. Building on these findings, the combination of CDNs with PD-L1 inhibitors, radiotherapy, or chemotherapy may produce synergistic effects (156). The advancement of next-generation STING agonists, presents novel strategies for the treatment of HCC, thereby making this combination of immunotherapy and conventional therapies worthy of further investigation (156).

**Table 4:** Summary of studies on the effects of STING in HCC.

Study	Disease	Agent	STING	Effects on tumor immunocytes	Ref.
Chen 2023	HCC	MOF-CpG-DMXAA NPs	Activation	A superior immunotherapeutic efficiency in orthotopic and recurrent HCC	(135)
Sun 2023	HCC	rgFlu/PD-L1	Activation	Enhancement of CD8+ T cell cytotoxicity	(136)
Sheng 2020	HCC	AZD6738 + RT+ anti-PD-L1	Activation	Improving TME after radioimmunotherapy Better efficacy in inhibiting tumor growth, prolonging survival and preventing recurrence of tumor mice	(137)
Zhang 2021	HCC	cAIMP	Activation	STING expression is positively correlated with CD8+ T cell infiltration in HCC	(138)
Du 2021	HCC	IFNAR1	Inhibition	Prevents RILD without impacting tumor response	(139)
Xu 2024	HCC	NH125	Activation	Enhancing the infiltration and activity of NK cells in HCC	(140)
Li 2022	HCC	ABX	Inhibition	A potential approach for modulating and predicting the efficacy of RT through the gut microbiome–liver axis	(141)
Song 2024	HCC	DPPA-1M@AIE-Mito-TPP NPs	Activation	Trigging incomplete mitophagy and inducing macrophage repolarization	(142)
Huang 2020	HCC	TT-LDCP NPs	Activation	Exhibiting immunoadjuvant properties and promoting CD8+ T cell infiltration	(143)
Li 2022	HCC	HBO+Teniposide	Activation	Enhancing CD8+ T cell cytotoxicity and improving the efficacy of PD-1 inhibitors	(144)
Zhao 2022	HCC	RNASEH2A KO	Activation	A positive correlation between poor prognosis	(145)
Wu 2023	HCC	IgG sialylation	Inhibition	Activating Raf-1-ATF3 and Inhibiting the antitumorigenic activities	(146)
Chan 2023	HCC	CAF-1 KO	Activation	Suppression of the CAF1/H3.1 axis may lead to the substitution of histone variants	(147)
Lasarte-Cia 2021	HCC	c-di-GMP + IRE	Activation	Enhancing antitumor immune response and promoting CD8+ T cell infiltration	(148)
Li 2024	HCC	NRF2	Inhibition	The failure of STING activation and tumor immune escape	(149)
Chen 2024	HCC	Olaparib	Activation	Activating neoantigen-specific CD8+ T cells and alleviating radiation-induced immune failure	(150)
Du 2022	HCC	RT	Activation	Resulting in PD-L1 upregulation and inhibiting cytotoxic T-cell activity	(151)
Hong 2024	HCC	RECQL4	Inhibition	Serving as a biomarker for HCC treatment monitoring and Repairs radiation-induced DNA damage	(152)
Wang 2022	HCC	STAT3 KO	Activation	Sorafenib triggers ER stress-induced apoptosis, thereby enhancing CD103+ DC recruitment.	(153)
Ao 2024	HCC	ALG@MSA-2	Activation	Enhancing M1 macrophage polarization and providing novel insights for the RFA treatment	(154)
Su 2023	HCC	TAK1^△HEP^	Activation	A novel method for regulating iron allergy and STING-dependent immunopathology	(155)
Thomsen 2019	HCC	CDNs	Activation	The combination of CDNs with PD-L1 inhibitors provides a new strategy for the treatment of HCC	(156)
Huang 2021	HCC	ICG-001 + IR	Activation	Dual effects of radiosensitization and immunological activation on HCC	(157)
Cen 2021	HCC	ZnS@BSA NPs	Activation	Promoting CD8+ T cell infiltration and facilitating DC cross-presentation	(158)
Takahashi 2018	HCC	DNase2/TREX1 downregulation	Activation	Investigating the key mechanisms of the SASP, triggered by abnormal HSC activation, and Exploring potential strategies for its control.	(159)

Sun et al. (2023) demonstrated that a PD-L1 antibody-expressing oncolytic virus (rgFlu/PD-L1) targets HCC cells by activating the cGAS-STING pathway, thereby enhancing CD8+ T cell function (136). Wu et al. (2023) identified a negative feedback loop in immune checkpoint blockade (ICB) therapy, wherein IgG sialylation induced by ICB engages DC-SIGN+ macrophages, thereby activating Raf-1/ATF3 signaling to suppress cGAS-STING activity and type-I interferon production. Importantly, the targeted disruption of this feedback loop significantly enhances macrophage type-I interferon responses and reinvigorates anti-tumor T cell immunity, suggesting a promising strategy to overcome ICB resistance (**Figure 2E**) (146). In contrast, Chan et al. (2023) demonstrated that Chromatin Assembly Factor 1 (CAF-1) facilitates DNA replication stress and chromosomal instability, which result in cytosolic DNA leakage, micronuclei formation, and subsequent activation of cGAS, ultimately increasing HCC sensitivity to ICB. A hallmark feature of the TME may modulate cGAS activity through mitochondrial DNA release and/or HIF-1α-mediated transcriptional regulation (147). Li et al. (2022) demonstrated that hyperbaric oxygen (HBO) therapy combined with teniposide enhances the efficacy of PD-1 antibodies by alleviating tumor hypoxia and augmenting chemotherapy-induced DNA damage-mediated STING activation. This combination ultimately enhances immunogenicity and promotes T cell infiltration (144). Furthermore, Zhao et al. (2022) established a connection between high RNASEH2A expression and the progression of HCC as well as poor prognosis. This suggests that RNASEH2A may promote immune evasion by restricting the release of cytosolic DNA and inhibiting cGAS activation (145). Li et al. (2024) discovered that mutations in NRF2 reduce STING expression and immune infiltration, thereby promoting immune evasion. They also demonstrated that the combination of NRF2 inhibitors with STING agonists could enhance CD8+ T cell activity (149). Wang et al. (2022) demonstrated that the knockout of STAT3 in HCC cells enhances sorafenib-induced endoplasmic ER stress and apoptosis, subsequently leading to DNA release and activation of the cGAS-STING pathway in CD103+ DCs. Low-dose sorafenib combined with STAT3 knockout therapy can directly eliminate tumor cells (153).

Early phase I clinical trials of STING agonists as monotherapy have demonstrated limited efficacy in solid tumors and lymphomas, highlighting the critical need for optimized delivery strategies (167). Nanomedicine presents innovative solutions to address this challenge (168–172). For example, Huang et al. (2020) engineered TT-LDCP nanoparticles for the targeted delivery of PD-L1 siRNA and IL-2-encoding plasmid DNA to HCC cells. This approach achieved dual activation of the cGAS-STING pathway and reshaped the tumor immune microenvironment, significantly enhancing the activation of tumor-infiltrating CD8+ T cells (143). Cen et al. (2021) utilized ZnS@BSA nanoclusters that release zinc ions in the acidic tumor microenvironment, leading to the accumulation of ROS and subsequent mitochondrial damage, as well as the release of mtDNA. This process effectively activates the cGAS-STING pathway, demonstrating potential for preventing tumor recurrence and metastasis in HCC (158). Lasarte-Cia et al. (2021) demonstrated that the combination of irreversible electroporation (IRE) and the STING agonist c-di-GMP significantly enhanced long-term survival in mice while promoting an increase in tumor-infiltrating activated CD8+ T cells (148). Chen et al. (2023) developed multifunctional MOF-CpG-DMXAA nanomedicines that serve dual roles as drug delivery vehicles and STING agonists. These nanomaterials effectively reprogram tumor-associated macrophages (TAMs), enhance DC maturation (135). Moreover, Song et al. (2024) utilized FDA-approved AIE-Mito-TPP drug delivery technology, which demonstrated potent capabilities in disrupting mitochondria. This disruption induced significant incomplete mitophagy and sustained leakage of mtDNA, effectively activating *in vitro* immune responses by promoting cGAS-STING activation, DC activation, and macrophage polarization (142). Ao et al. (2024) developed an injectable alginate hydrogel designed for the sustained release of the STING agonist MSA-2. This innovative approach successfully promotes macrophage M1 polarization and enhances cytotoxic T lymphocyte infiltration, thereby improving the TME. Consequently, it serves as a valuable adjunct to radiofrequency ablation therapy for HCC (154). Mn²^+^, when combined with tumor-disrupting adjuvants, forms antitumor nanoregulators that cascade-activate the cGAS-STING pathway, thereby enhancing immunotherapeutic efficacy against cancer. Notably, mitoxantrone (MIT) has demonstrated potential to stimulate cGAS-STING signaling, with studies confirming the feasibility of co-delivering MIT and NAP via nanoparticles for synergistic chemoimmunotherapy in HCC (166). Nevertheless, several challenges must be addressed before successful clinical translation. First, the tissue selectivity and cellular uptake efficiency of these delivery systems require rigorous validation within the metabolically active and detoxification-intensive hepatic environment. As a central component of innate immunity, uncontrolled activation of the cGAS–STING pathway can trigger severe systemic inflammatory responses, such as cytokine storms, leading to high fever, fatigue, and potentially multi-organ damage. Although nanomedicine can reduce systemic exposure through targeted delivery, excessive local release of STING agonists may still induce intense inflammation in the peritumoral region, causing collateral injury to normal tissues. Moreover, certain agonists—such as DMXAA—exhibit strict species specificity, being effective only in murine STING, thereby limiting direct translation to human applications. Safety considerations are equally critical. Nanomaterials and delivery systems may introduce additional toxicity risks. Mitochondria-targeting strategies, such as ZnS@BSA and AIE-Mito-TPP, while effective in promoting mitochondrial DNA leakage and cGAS–STING activation, may also impair mitochondrial function in normal cells and cause long-term metabolic disturbances. Furthermore, the *in vivo* biodistribution, degradation products, and potential immunogenicity of nanoparticles constitute essential components of comprehensive safety evaluation.

Radioimmunotherapy presents potential in the HCC treatments, primarily due to the complex modulation of the TME by radiotherapy (173–176). However, the activation of DNA damage repair (DDR) pathways triggered by ionizing radiation (IR) can, paradoxically, inhibit the immune microenvironment, thereby reducing the anti-tumor efficacy of radioimmunotherapy. Importantly, the adverse effects of radiotherapy on healthy tissues, particularly the high radiosensitivity and vulnerability to radiation-induced liver disease (RILD), continue to pose a major challenge in cancer treatment (163, 177, 178). The study conducted by Du et al. (2022) demonstrated that the combination of radiotherapy with anti-PD-1/PD-L1 blockade significantly enhances therapeutic efficacy in HCC. This enhancement is associated with increased infiltration of cytotoxic T lymphocytes (CTLs) within the TME and is dependent on functional CD8+ T cells (151). However, Du et al. (2021) highlighted that this therapeutic approach may simultaneously activate the cGAS-STING pathway, which senses hepatocyte-derived DNA damage and amplifies IFN-I signaling, ultimately contributing to the pathogenesis of RILD (139). Chen et al. (2024) demonstrated that the PARP inhibitor olaparib effectively sensitizes BRCA-deficient HCC cells to radiotherapy. This combined treatment induces significant DNA damage, thereby activating the cGAS-STING pathway and remodeling the tumor immune microenvironment (150). Similarly, Sheng et al. (2020) indicated that the ATR inhibitor AZD6738 may synergistically enhance the anti-tumor effects of radioimmunotherapy through the activation of the cGAS-STING pathway (137). Huang et al. (2021) demonstrated that the Wnt/β-catenin signaling pathway inhibitor ICG-001, when used in conjunction with radiotherapy, enhances the DNA damage response and increases the radiosensitivity of HCC by inhibiting the activation of the p53-mediated cGAS-STING pathway (157). Given the complex tumor-driving alterations induced by radiotherapy, Hong et al. (2024) identified RECQL4 as a crucial DDR marker in malignant HCC cells through the application of single-cell RNA sequencing (scRNA-seq). They demonstrated that tumor-derived RECQL4 inhibits the activation of the DC cGAS-STING pathway, thereby interfering with anti-tumor immune reconstitution following radiotherapy and ultimately reducing HCC sensitivity to treatment (152). Furthermore, extratumoral factors can significantly influence the efficacy of radiotherapy. Li et al. (2022) demonstrated that gut microbial metabolites can interfere with radiotherapy-induced cGAS-STING pathway in DCs, subsequently weakening antigen presentation and T cell function, ultimately compromising the anti-tumor immune efficacy of radiotherapy (141). In summary, the cGAS-STING pathway holds significant potential for application in cancer therapy; however, its complexity necessitates a more nuanced and personalized approach in both research and clinical practice.

### Parasitic liver disease

The cGAS-STING pathway is implicated not only in immune defense during schistosomiasis but also appears to be closely associated with liver pathological alterations (**Table 3**; **Supplementary Table 7**). Souza et al. (2020) revealed that the cGAS–STING pathway recognizes the DNA of *Schistosoma mansoni*, leading to the activation of host cells and the initiation of immune responses. In STING-deficient mice, a more pronounced pro-inflammatory phenotype was observed, characterized by increased levels of splenic IFN-γ, elevated proportions of neutrophils, and inflammatory features within the gut microbiota (130). Liang et al. (2022) demonstrated that cGAS plays a crucial role in sensing schistosome-derived DNA and regulating type I interferon production. Notably, cGAS deficiency significantly improved the survival rates of mice infected with *Schistosoma japonicum* and attenuated liver injury and fibrosis, whereas STING deficiency primarily affected egg granuloma formation. Furthermore, evidence suggests that cGAS exerts functions independent of STING in promoting liver fibrosis, indicating that the roles of cGAS and STING may be uncoupled in various biological processes (**Figure 2F**) (129, 179). Studies by Jin et al. and Chen et al. have revealed how Toxoplasma gondii ROP effectors facilitate infection, respectively: ROP5 modulates the infection mechanism, while ROP18I suppresses host type I interferon responses for immune evasion (180, 181). During Plasmodium infection, the cGAS-STING pathway exhibits dual and seemingly contradictory immunoregulatory effects. On one hand, its protective role manifests during the adaptive immune phase: in blood-stage infection, parasite-derived DNA is sensed by cytosolic cGAS in host cells, providing critical immune stimulation for downstream B cell responses. This signaling promotes the maturation and selection of B cells within the germinal center (GC), leading to the generation of high-affinity, long-lived antibodies that effectively restrict parasite replication and establish protective immunity. However, paralleling this immune-enhancing effect is its potential pathogenic role: hemozoin (Hz), a metabolic byproduct of the malaria parasite, frequently carries parasite genomic DNA and can be phagocytosed by host cells. When these complexes gain access to the cytosol, they are recognized by cGAS, resulting in robust STING pathway activation. In certain critical cell types—including hepatocytes, neurons, and astrocytes—this signaling does not elicit protective immune responses but instead triggers apoptosis and proinflammatory cytokine release, culminating in tissue destruction and organ dysfunction, such as the neuropathology observed in cerebral malaria and hepatic tissue damage (10). The same immune-sensing axis induces diametrically opposite immune outcomes depending on the cellular context, infection stage, or parasite strain involved. In-depth investigation of this bidirectional regulatory mechanism will help elucidate the delicate balance between innate and adaptive immunity during parasitic infections and provide a theoretical foundation for designing more precise immunotherapeutic strategies.

## Current clinical landscape of STING agonists

MIW815 (ADU-S100) is the first synthetic cyclic dinucleotide (CDN) STING agonist to enter clinical trials, marking the beginning of therapeutic exploration targeting the STING pathway in cancer immunotherapy. In the first-in-human study, MIW815 monotherapy demonstrated limited clinical activity and was insufficient to overcome immune resistance. Nevertheless, evident systemic immune activation was observed, supporting its combination with PD-1 inhibitors. Subsequent trials confirmed that MIW815 combined with spartalizumab was well tolerated in patients with advanced or metastatic malignancies, including PD-1–refractory disease, although antitumor responses remained modest (182, 183). These clinical experiences have guided the development of second- and third-generation STING agonists, particularly those amenable to intravenous administration. Ulevostinag (MK-1454), an optimized synthetic CDN, is administered via intratumoral injection. A phase I open-label study (NCT03010176) evaluated MK-1454 alone and in combination with pembrolizumab in patients with advanced or metastatic solid tumors or lymphomas, revealing immunostimulatory potential but necessitating monitoring for systemic toxicity. In a subsequent phase II trial (NCT04220866), 4 of 8 patients treated with the combination achieved complete or partial responses, whereas only 1 of 10 receiving pembrolizumab alone responded (184).

In another study (NCT03249792), MK-2118 was assessed as an intratumoral monotherapy, in combination with pembrolizumab, and as a subcutaneous formulation combined with pembrolizumab. The incidence of grade 3/4 treatment-related adverse events was 22%, 23%, and 11%, respectively, indicating manageable toxicity yet limited antitumor activity (185). IMSA-101 was investigated in a phase I/IIa study (NCT04020185) in patients with advanced refractory malignancies to evaluate its safety and efficacy. The ongoing phase II trial (NCT05846659) is exploring an innovative combinational strategy of radiotherapy (PULSAR) plus PD-1 inhibition to potentiate STING activation in oligoprogressive solid tumors.

Building on first-generation experiences, second-generation STING agonists have improved pharmacokinetic properties, stability, and delivery routes. BMS-986301 (NCT03956680) is under phase I evaluation as monotherapy and in combination with nivolumab or ipilimumab, to determine the optimal route of administration (systemic vs. intratumoral) (186). Comparative studies by Li et al. assessed hepatic/intratumoral versus intramuscular systemic administration, providing references for clinical strategy optimization. GSK3745417 is undergoing first-in-human trials in advanced solid tumors (NCT03843359) and phase I combination studies (CTIS2024-513574-21-00); results are pending but aim to establish intravenous/systemic delivery feasibility and inform future non-nucleotide agonist design. DN015089 (CTR20212462) is being evaluated in a multicenter phase Ia/Ib study for safety, tolerability, pharmacokinetics/pharmacodynamics, and preliminary efficacy in advanced solid tumors after standard-of-care failure. KL340399 is under two phase I intratumoral trials (NCT05549804, NCT05387928) assessing safety and early efficacy. XA5508, a liposomal complex combining a STING agonist and an anti–PD-L1 nanobody, is being investigated for its antitumor mechanism and efficacy against HCC, representing an innovative delivery approach (187).

Third-generation STING agonists, characterized by small-molecule, non-CDN structures suitable for systemic administration, aim to prolong STING activation, minimize off-target effects, and enhance the controllability of antitumor immune responses. TAK-676, administered intravenously, induces durable STING activation and is currently under a phase I/II trial (NCT05070247) evaluating monotherapy and combination with pembrolizumab in patients with locally advanced or metastatic solid tumors (target enrollment: 106) (188). SB11285 (NCT04096638) is also in phase Ia/Ib dose-escalation trials, assessing safety alone or with atezolizumab, with results yet to be reported. E7766, evaluated for intratumoral injection (NCT04144140), was terminated for non-safety reasons. DW18343 has demonstrated systemic antitumor efficacy in preclinical models (189). Preclinical investigations include ALG-03104, which exhibits potent antitumor activity in murine models; INDP-010, capable of inducing systemic innate and adaptive immune activation across animal and human systems via intravenous delivery and approved by the U.S. FDA to enter clinical trials (190); and Licochalcone B (LicoB), which shows hepatoprotective and anti-hepatocarcinoma effects in HepG2 cells and CCl_4_-induced acute liver injury mice, suggesting new perspectives for STING modulation in liver diseases. To date, all STING agonists remain in early clinical development and primarily target malignancies, with none approved for liver cancer or viral hepatitis (191). From the first-generation CDN compounds with local administration to the optimized second-generation CDN analogs exploring diverse delivery routes and the third-generation non-CDN small molecules enabling systemic use, STING agonist research reflects a clear trajectory of optimization. Combination therapy with immune checkpoint inhibitors has emerged as the mainstream strategy, while overcoming immune resistance and improving clinical efficacy remain the central challenges for future development.

## Concluding remarks and future perspectives

This review comprehensively examines the multifaceted roles of the cGAS-STING pathway in hepatic diseases, highlighting its crucial involvement in disease initiation, progression, and therapeutic strategies. Through systematic analysis, we identify a dual role of cGAS-STING, exhibiting either pro-inflammatory or immunosuppressive effects based on disease stage and microenvironment. Our analysis reveals the need for further research to elucidate the pathway’s role across various liver cell types, its interactions with other signaling cascades, and its behavior during disease progression. Optimizing therapeutic strategies requires precise modulation of pathway activation, achieved through next-generation STING agonists with improved pharmacokinetic profiles, rational combination regimens (e.g., ICB synergy), and optimized delivery modalities. While STING activation offers substantial immunotherapeutic potential, chronic or uncontrolled stimulation may induce detrimental consequences, including autoimmune reactions and RILD. Sustained interferon signaling can disrupt immune tolerance and hepatic homeostasis, underscoring the need to balance antitumor efficacy with systemic safety. Future strategies should focus on context-specific modulation of STING activity—optimizing dose, duration, and cell-targeting approaches to preserve therapeutic benefit while minimizing toxicity, ultimately improving patient outcomes and quality of life.
