# Supplementary material for: Molecular mechanisms and therapeutic strategies of cGAS-STING pathway in liver disease: the quest continues
Source: Front Immunol. 2025 Dec 8;16:1692365. doi: 10.3389/fimmu.2025.1692365 (PMC12719526; doi:10.3389/fimmu.2025.1692365)
Supplement: Supplementary file 1 [file DataSheet1.docx]

**Supplementary Information for:**

Molecular mechanisms and therapeutic strategies of cGAS-STING pathway in liver disease: the quest continues.

*Yichen Fan^1^, Zihao Dong^2^, Yufeng Wu^2^, Hao Wen^1^**

1 State Key Laboratory of Pathogenesis, Prevention and Treatment of High Incidence Diseases in Central Asia, Clinical Medicine Institute, The First Affiliated Hospital of Xinjiang Medical University, Urumqi, Xinjiang, China.

2 The First Affiliated Hospital of Xinjiang Medical University, Urumqi, Xinjiang, China.

*Corresponding authors: Hao Wen.

E-mail: dr.wenhao@163.com (Hao Wen).

# Table of contents

**Supplementary Tables 1.** Summary of key findings from studies investigating the role of cGAS-STING pathway in NAFLD and ALD.

**Supplementary Tables 2.** Summary of key findings from studies investigating the role of cGAS-STING pathway in Hepatitis.

**Supplementary Tables 3.** Summary of key findings from studies investigating the role of cGAS-STING pathway in Chemical and Drug Induced Liver Injury.

**Supplementary Tables 4.** Summary of key findings from studies investigating the role of cGAS-STING pathway in HIRI

**Supplementary Tables 5.** Summary of key findings from studies investigating the role of cGAS-STING pathway in Liver Neoplasms.

**Supplementary Tables 6.** Summary of key findings from studies investigating the role of cGAS-STING pathway in Liver Neoplasms.

**Supplementary Tables 7.** Summary of key findings from studies investigating the role of cGAS-STING pathway in Parasitic liver disease.

**Supplementary Tables 8-1** PRISMA 2020 checklist.

**Supplementary Tables 8-2** PRISMA 2020 for Abstracts checklist.

**Supplementary Table 9-1.** Search strategies for Hepatitis and STING in Medical Subject Headings.

**Supplementary Table 9-2.** Search strategies for Liver Neoplasms and STING in Medical Subject Headings.

**Supplementary Table 9-3.** Search strategies for NAFLD/ALD and STING in Medical Subject Headings

**Supplementary Table 9-4.** Search strategies for Liver Cirrhosis and STING in Medical Subject Headings.

**Supplementary Table 9-5.** Search strategies for HIRI and STING in Medical Subject Headings.

**Supplementary Table 9-6.** Search strategies for C-DILI and STING in Medical Subject Headings.

**Supplementary Table 9-7.** Search strategies for Parasitic Liver Disease and STING in Medical Subject Headings.

**Supplementary Table 10.** Eligibility criteria.

**[Supplementary Tables 11-1.](#_Table_1.__1)**  [List of excluded studies in NFLD/ALD.](#_Table_1.__1)

**[Supplementary Tables 11-2.](#_Table_2._)**  [List of excluded studies in Hepatitis.](#_Table_2._)

**[Supplementary Tables 11-3.](#_Table_3._)**  [List of excluded studies in Liver Neoplasms.](#_Table_3._)

**[Supplementary Tables 11-4.](#_Table_4._)**  [List of excluded studies in](#_Table_4._)  C-DILI

**[Supplementary Tables 11-5.](#_Table_5._)**  [List of excluded studies in Liver Cirrhosis.](#_Table_5._)

**[Supplementary Tables 11-6.](#_Table_6._)**  [List of excluded studies in HIRI.](#_Table_6._)

**[Supplementary Tables 11-7.](#_Table_6.__1)**  [List of excluded studies in Parasitic Liver Disease.](#_Table_6.__1)

**Supplementary Table 12.**  List of secondary exclusion.

# Figure of contents

**Supplementary Fig 1.** Risk of bias trends across included studies using the SYRCLE tool.

**Supplementary Fig 2.** ARRIVE 2.0 reporting quality assessment across included studies.

**Supplementary Table 1.** Summary of key findings from studies investigating the role of cGAS-STING pathway in NAFLD and ALD.

| **Item** | **Study** | **Title** | **Disease** | Treatment/Method | Key Findings | Treatment/Method | Key Findings | Treatment/Method | Key Findings | Treatment/Method | Key Findings | Treatment/Method | Key Findings |
| --- | --- | --- | --- | --- | --- | --- | --- | --- | --- | --- | --- | --- | --- |
| #1 | Luo et al., 2018 | Expression of STING Is Increased in Liver Tissues From Patients With NAFLD and Promotes Macrophage-Mediated Hepatic Inflammation and Fibrosis in Mice | NAFLD/NASH | 1.HFD/STINGgt mice | **Down-regulation:**  weight gain, ALT , liver weight, intrahepatic triglyceride, fat deposits in the liver, F4/80, JNK p46, NFkB p65, TNFa, IL1b, IL6 and FAS. | 2.HFD ( BMT-WT)/STINGgt mice | **Up-regulation:** F4/80, JNK p46 and NFkB p65, NFa, IL1b, IL6 and FAS. **Unchanged indicators：**weight gain, ACC1, carnitine palmitoyltransferase 1a, and sterol regulatory element-binding protein 1c. | 3.MCD/STINGgt mice | **Down-regulation:** ALT, liver weight, intrahepatic triglyceride levels, F4/80 positive cells, NK p46, NFkB p65, TNFa, IL1b, Col1a1, and Fn. | 4.DMXAA-STINGgt/BMDM-CM | **Down-regulation:**  JNK p46 and NFkB p65, TNFa, IL1b, IL6, ACC1, FAS, TGF-β1, Fn. | 5.Co-culture of LX2 cells with DMXAA-STINGgt/BMDM-CM.(NASH) | **Down-regulation:**  Col1a1, Fn, TGFb1. |
| #2 | Qiao et al., 2018 | Activation of the STING-IRF3 pathway promotes hepatocyte inflammation, apoptosis and induces metabolic disorders in nonalcoholic fatty liver disease | NAFLD | 1.FFA/STING siRNA | **Down-regulation:**  IRF3, IFN-β, p-p65/p65, IL6, IL1b, Bax/Bcl2, clv-Casp3/Casp3, clv-PARP/PARP, p-AKT/t-AKT, p-GSK3β, GCK, PFK, PK, G-6-pase, PEPCK, PC | 2.FFA/IRF3 siRNA | **Down-regulation:**  IRF3, IFN-β, p-p65/p65, IL6, IL1b, Bax/Bcl2, clv-Casp3/Casp3, clv-PARP/PARP, p-AKT/t-AKT, p-GSK3β, GCK, PFK, PK, G-6-pase, PEPCK, PC |  |  |  |  |  |  |
| #3 | Liu et al., 2022 | Aucubin administration suppresses STING signaling and mitigated high-fat diet-induced atherosclerosis and steatohepatosis in LDL receptor deficient mice | NAFLD/NASH | 1.Aucubin administration-HFD/LDLr mice | **Up-regulation:** miR-181a-5p. **Down-regulation:** TNF-α, ALT, AST, IL1b, IL6, Tnf. | 2.miR-181a-5p-mtDNA/BMDM | **Down-regulation:** NFκB, Tnf, IL1b, IL6 |  |  |  |  |  |  |
| #4 | Qi et al., 2022 | Curcumol Suppresses CCF-Mediated Hepatocyte Senescence Through Blocking LC3B–Lamin B1 Interaction in Alcoholic Fatty Liver Disease | ALD | 1.Curcumol-Ethanol/ transfected LO2 cells with cGAS plasmid | **Up-regulation:** SA-β-gal, lipid accumulation, p21, p16, TRF1. **Down-regulation:** TERT, TRF2 |  |  |  |  |  |  |  |  |
| #5 | Wang et al., 2024 | Flavonoid extracted from Epimedium attenuate cGAS-STING-mediated diseases by targeting the formation of functional STING signalosome | NAFLD/NASH | 1.EF-( ISD, cGAMP, DMXAA, diABZI)/BMDMs, THP-1, PBMCs | **Down-regulation:** IRF3, STING, IFN-β, CXCL10, IL6, TNF-α | 2. EF-MCD/NASH mice | **Down-regulation:** F4/80 positive cells, α-SMA, Col1a1, IL6, TNF-α. Up-regulation: ALT, AST |  |  |  |  |  |  |
| #6 | Patel et al., 2018 | HEPATIC GAP JUNCTIONS AMPLIFY ALCOHOL-INDUCED LIVER INJURY BY PROPAGATING CGAMP-MEDIATED IRF3 ACTIVATION | ALD | 1.LD/cGAS KO mice | **Down-regulation:** IRF3, ALT, AST, IFN-β, IFIT2, IFIT3, Liver injury degree |  |  |  |  |  |  |  |  |
| #7 | Luo et al., 2023 | Licorice extract inhibits the cGAS-STING pathway and protects against non-alcoholic steatohepatitis | NAFLD/NASH | 1.LE/HT DNA-BMDMs | **Down-regulation:** IRF3, Ifnb, IFN-β, P65, TNF-α, IL6, CXCL10, ISG15 | 2.LE-(2'3'-cGAMP, DMXAA, diABZI)/BMDMs | **Down-regulation:** IRF3, Ifnb, IFN-β, P65, TNF-α, IL6, CXCL10, ISG15 | 3.LE-MCD/NASH mice | **Down-regulation:** AST, ALT, α-SMA, Col1a1, p-IRF3, IL6, TNF-α | 4.C-176-MCD/NASH mice | **Down-regulation:** AST, ALT, α-SMA, Col1a1, p-IRF3, IL6, TNF-α |  |  |
| #8 | Li et al., 2022 | Iron Activates cGAS-STING Signaling and Promotes Hepatic Inflammation | Chronic liver disease （NFALD/HCC/ALD/Hepatitis） | 1.FAC/HepG2 | **Up-regulation:** STING, cGAS, TBK1, IRF3, NFkB, IFN-β, IL6, STAT1 | 2.FAC/mice | **Up-regulation:** STING, cGAS, TBK1, IRF3, NFkB, IFN-β, IL6, STAT1 |  |  |  |  |  |  |
| #9 | cho et al., 2018 | Lipotoxicity induces hepatic protein inclusions through TANK binding kinase 1–mediated p62/sequestosome 1 phosphorylation | NAFLD/NASH | 1.PA-SFA+sh STING/HepG2 | **Down-regulation:** p-TBK1, p-p62 | 2.PA-SFA+sh cGAS/HepG2 | **Down-regulation:** p-TBK1, p-p62 | 3.PA-SFA+Sting or cGAS KO/mice | **Down-regulation:** p-TBK1, p-p62 | L-TBK1-KO/mice | **Down-regulation:** p-TBK1, p-p62 | 5.CD-HFD+BX795/NASH mice | **Down-regulation:** p-p62, TNF-1, IL6, IL10, α-SMA, Col1a1, Col3a1, MMP-2, MMP-9, TIMP-2, LOX, CTGF, PECAM-1 |
| #10 | Ma et al., 2023 | Loss of hepatic DRP1 exacerbates alcoholic hepatitis by inducing megamitochondria and mitochondrial maladaptation | ALD/AH | 1.EtOH-L-DRP1 KO/mice | **Up-regulation:** Mitochondria size, AST, ALT, Col1a1, Acta2, Tgfb, LC3-II, p62. **Down-regulation:**  Red-only mitochondria number, MFN1, MFN2 | 2.EtOH-L-DRP1 KO/mice (NPCs, Liver nuclear, mtDNA) | **Up-regulation:** (mtDNA) 16S, ND1, Loop1, Loop3. (Nuclear) IRF-3. (NPCs) IRF-7, STING, cGAS. | 3.EtOH-L-DRP1 KO/mice (proinflammatory microenvironment) | **Up-regulation:** IL1b, Tnfa, Ccl2, Ccl4, Icam1, Vcam1, 12-HEPE, 5-HETE, 15-HETE, Ddit3, sXbp1/ uXbp1, BIP, p-Eif2α, caspase3/7, TUNEL, IL-13, IL-16, IL-1F3, CXCL-9, CXCL-10, CCL5. |  |  |  |  |
| #11 | Huang et al., 2022 | Macrophage SCAP Contributes to Metaflammation and Lean NAFLD by Activating STING–NF-κB Signaling Pathway | NAFLD | 1.PD-SCAPΔMφ/mice | **Down-regulation:** p-p65, N-P65, P-IKKα/β, p-IKBα, Ewat, F4/80, α-SMA, ALT, Col1a1, Col4a4, TGF-β, TNF-α, IL1b, IL6, MCP-1, p-TBK1 | 2.OE-SCAPi/ RAW264.7 | **Down-regulation:** TNF-α, IL1b, IL6, MCP-1, p-p65, p-TBK1, P-IKKα/β, p-IKBα. |  |  |  |  |  |  |
| #12 | Luo et al., 2022 | Microbial DNA enrichment promotes liver steatosis and fibrosis in the course of non-alcoholic steatohepatitis | NAFLD/NASH | 1.NASH mEVs/ mice, hep | **Up-regulation:** cGAS, p-sting | 2.24wks WD NASH-cGAS KO/mice | **none:** Sirius Red or Masson's trichrome staining signals | 3.NASH mEVs-siRNA Cgas/ hep | **Down-regulation:** Col1a1, IL1b, Tnfa, Acta2, Pai1 |  |  |  |  |
| #13 | Yang et al., 2023 | The macrophage STING-YAP axis controls hepatic steatosis by promoting the autophagic degradation of lipid droplets | NASH | 1.HFD-STING KO/ NASH mice | **Down-regulation:** p-TBK1, p-LATS1, P-YAP, Body weight, TG, TC, Blood glucose, FFA, ALT, Masson, Fabp1, Fas, FAT, CD36, Humgcr | 2.HFD-(STING-YAP MDKO)/NASH mice | **Up-regulation:** Body weight, Blood glucose, ALT, FFA, TG, TC, NFALD Activity score, ACC-α, SQSTM1-P62, PLIN2. **Down-regulation:** ACS-1, CPT-1α, ECH1, LCAD, ATG7, ATG5, LAMP1, LC3B-2/LC3B-1. | 3.PA/OA-(STING-YAP MDKO)/macrophage-hepatocyte co-culture system | **Up-regulation:** PLIN2, 3QSTM1, TG. **Down-regulation:** ATG7, ATG5, LC3B | 4.PA/OA-(STING-YAP MDKO)/BMM | **Down-regulation:** TMEM205 |  |  |
| #14 | Ribeiro et al., 2023 | Protective role of cGAS in NASH is related to the maintenance of intestinal homeostasis | NASH | 1.16w(HF-HC-HSD)-cGAS KO/NASH mice | **Up-regulation:** ALT, TG, MCP-1, IL1b, Collagen, Sirus red | 2.MCD-cGAS KO/NASH mice | **Up-regulation:** ALT, TG. **Down-regulation:** IFN-β, TNF-α. | 3.16w and 30w(HF-HC-HSD)-STING KO/NASH mice | **Up-regulation:** ALT, TG, Oil-Red-O. **Down-regulation:** MCP-1, IL-1b. | 4.8w(MCD)-cGAS KO/NASH mice | **Up-regulation:** α-SMA, Collagen. |  |  |
| #15 | Li et al., 2020 | Remdesivir attenuates high fat diet (HFD)-induced NAFLD by regulating hepatocyte dyslipidemia and inflammation via the suppression of STING | NFALD | 1.HFD-RDV/mice | **Down-regulation:** ALT, AST, TG, TC, LDL-C, HMGCR, SREBF1, FASN, SCD1, PPARγ, CD36, FABP1, IL18, IL6, IL1b, TNF-α, CXCL-10, F4/80, STING, p-TBK1, p-IRF3, IFN-β， p-NFkB. **Up-regulation:** HDL-C, CPT1α，PPARα, ACOX1 | 2.PA-RDV/Kupffer, Hep | **Down-regulation:** IL18, IL6, IL1b, TNF-α, CXCL-10, STING | 3.PA-(STING siRNA)/Hep | **Down-regulation:** SREBF1, FASN, SCD1, CD36, IL18, IL6, IL1b, TNF-α, CXCL-10, p-TBK1, p-IRF3, IFN-β, p-NFκB. **Up-regulation:** CPT1α, ACOX1, PPARα | 4.PA-RDV-oeSTING/Hep | **Up-regulation:** SREBF1, FASN, SCD1, CD36, IL18, IL6, IL1b, TNF-α, CXCL-10, p-TBK1, p-IRF3, IFN-β, p-NFκB. **Down-regulation:** CPT1α, ACOX1, PPARα |  |  |
| #16 | Donne et al., 2022 | Replication stress triggered by nucleotide pool imbalance drives DNA damage and cGAS-STING pathway activation in NAFLD | NFALD | 1.HFHS, CDHFD/mice, primary hepatocytes | **Up-regulation:** CHK1, RPA32, PCNA, γH2aX. Down-regulation: dATP, dTTP | 2.dNTPs-(HFHS, CDHFD)/primary hepatocytes | **Down-regulation:** p-CHK1, γH2AX | 3.RS-(HFHS, CDHFD)/primary hepatocytes | **Up-regulation:** STING, H3, cGAMP(60h), IFN-β | 4.dNTPs-RS-(HFHS, CDHFD)/primary hepatocytes | **Down-regulation:** STING, cGAMP, IFN-β |  |  |
| #17 | Lin et al., 2023 | RING finger protein 13 protects against nonalcoholic steatohepatitis by targeting STING-relayed signaling pathways | NFALD/NASH | 1.PAOA-AdshRnf13/Hep | **Up-regulation:** Nile red, TG, Srebf1, Fasn, Acaca, Pparg, Scd1, TNF-α, IL1b, IL6, Cxcl12, Mcp-1, STING, IFN-β | 2.HFD-Rnf13 HKO/mice | **Up-regulation:** Blood glucose, TC, TG, Liver weight, Acaca, Scd1, Fasn, Pparg, Cd36, AST, ALT Down-regulation: Cpt1α, Ppara | 3.HFD-Rnf13 HKO/mice | **Up-regulation:** Liver weight, TG, TC, LDL-C, Acaca, Scd1, Fasn, Pparg, Cd36, Tnfa, Cxcl2, Cxcl10, Ccl2, Ccl5, Col1a1, Col3a1, Acta2, Ctgf, Timp1. **Down-regulation:** Cpt1α, Ppara | 4.HFHC-Rnf13HepTg/mice | **Down-regulation:** Blood glucose, Liver weight, TG, TC, LDL-C, ALT, AST, Acaca, Scd1, Fasn, Pparg, Cd36, Acaca, Scd1, Fasn, Pparg, Cd36, Tnfa, Cxcl2, Cxcl10, Ccl2, Ccl5, Col1a1, Col3a1, Acta2, Ctgf, Timp1. **Up-regulation:** Cpt1α, Ppara | 5.PAOA-AdSTING1/Kupffer | **Up-regulation:** TG, Nile Red, TNF-α, IL1b, IL6, Cxcl12, Ccl5, Srebf1, Fasn, Acaca, Scd1, Pparg |
|  |  |  |  | 6.HFHC-Rnf13 HKO/mice | **Up-regulation:** STING, p-TBK1, IFN-β, p-p65. Down-regulation: RNF13, IκBα | 7.HFHC-Rnf13 HepTg/mice | **Down-regulation:** STING, p-TBK1, IFN-β, p-p65. Up-regulation: RNF13, IκBα | 8.HFHC-Rnf13-STING1/mice | **Up-regulation:** Blood glucose, TC, Liver TG, Liver weight, Acaca, Scd1, Fasn, Cd36, Tnfa, Ccl2, Col1a1, Col3a1, Ctgf, Timp1, IL1b, IL6 |  |  |  |  |
| #18 | Miao et al., 2023 | Role of Selenoprotein W in participating in the progression of non-alcoholic fatty liver disease | NFALD/NASH | 1.HFD-SelW KO/mice, BMDMs | **Down-regulation:** cGAS, STING, CD206+, CD86+, IL1b, CXCL9, ARG1, IL10 | 2.SelW Overexpressed-mtDNA/BMDMs | **Up-regulation:** cGAS, STING |  |  |  |  |  |  |
| #19 | Wang et al., 2020 | STING expression in monocyte-derived macrophages is associated with the progression of liver inflammation and fibrosis in patients with nonalcoholic fatty liver disease | NFALD/NASH | 1.PA-cGAMP/THP1, LX2 | **Up-regulation:** p-TBK1, α-SMA, IL6, IL1b, Col1a1, Fn, TGFβ1 |  |  |  |  |  |  |  |  |
| #20 | Petrasek et al., 2013 | STING-IRF3 pathway links endoplasmic reticulum stress with hepatocyte apoptosis in early alcoholic liver disease | ALD | 1.4wEthand/mice (protection from TNF-α induction) | IRF3-KO: -100%, ASC-KO: -99%, Casp-1-KO: -84%, IL-IRI-KO: -96% | 2.4wEthand/mice (protection from ALT induction) | IRF3-KO: -77%, ASC-KO: -42%, Casp-1-KO: -52%, IL-IRI-KO: -53% | 3.Ethanol/mice | Association between IRF3 and STING or TBK1: Whole cell liver extract, Endoplasmic retic. extract (early after ethanol adminstration. No Association between IRF3 and STING or TBK1: Mitochondral extract | 4.Thapsigargin-Temem173 gt/mice | **Down-regulation:**  p-IRF3/IRF3 | 5.Ethanol-Temem173 gt/mice | **Down-regulation:**  ALT, Ifnb-1, Isg15 |
| #21 | Wang et al., 2022 | STING-mediated inflammation contributes to Gao binge ethanol feeding model | ALD | 1.EtOH-fed/KCs | **Up-regulation:** STING | 2.mtDNA-STING overexpressed/Raw264.7 cell | **Up-regulation:** STING, IL6, IL1b, TNFα | 3.EtOH-fed-Raav8-STING/mice | **Down-regulation:** Apoptotic cell, AST, ALT, TG, IL6, TNF-α, IL1b, BAX/Bcl2, Caspase-3 | 4.Etoh-fed DMXAA/mice | **Up-regulation:** Oil red O, TUNEL, Apopotic cell, AST, ALT, TG, IL6, IL1b, TNFα, BAX/Bcl2, Caspase-3 | 5.mtDNA-RAW2647-CM/AML12 | **Up-regulation:** BAX/Bcl2, Caspase-3, TUNEL, QI-URC(31.95%), |
|  |  |  |  | 6.(EtOH-fed, mtDNA, Pegfp)/mice | **Up-regulation:** STING, p-TBK1 (There were no differences in mtDNA.), p-65, p-IRF3. | 7.STING siRNA/Raw264.7 cell | **Down-regulation:** STING, p-TBK1, p-65, p-IRF3. |  |  |  |  |  |  |
| #22 | Yu et al., 2019 | STING-mediated inflammation in Kupffer cells contributes to progression of nonalcoholic steatohepatitis | NFALD/NASH | 1.HFD-Tmem173gt/mice | **Down-regulation:** Body weight, AST, ALT, Cholesterol, Triglycerides, Hydroxyproline, LDL, F4/80, TNF-α, IL6, α-SMA, Col1a1 | 2.HFD-mtDNA-Tmem173gt/KCs | **Down-regulation:** NF-κB, TNF-α, IL6 | 3.HFD-mtDNA-TLR9 siRNA/KCs | **Down-regulation:** TNF-α, IL6, IL1b |  |  |  |  |
| #23 | Zhang et al., 2022 | The Absence of STING Ameliorates Non-Alcoholic Fatty Liver Disease and Reforms Gut Bacterial Community | NFALD | 1.HFD-STINGgt/mice | **Down-regulation:** Body weight, Body weight gain, Liver weight, Liver triglyceride, ALT, AST, TNF-α, IL1b, IL1a, IFN-g, CD3 T cell, CD8 T cell | 2.HFD-STINGgt/mice (Relative abundance) | **Up-regulation:** Prevotellaceae, Lactobacillaceae. **Down-regulation:** Lachnospiraceae, Rikenellaceae, Bacteroidaceae |  |  |  |  |  |  |
| #24 | Siao et al., 2022 | The Role of STING in Liver Injury Is Both Stimulus- and Time-Dependent | NFALD/NASH and Toxic liver injury | 1.72hTM-Tmem173gt/mice | **Down-regulation:** ALT | 2.High fructose feeding-Tmem173gt/mice | **Unchanged indicators:** ALT, TBK1, Irf3, Ifnb | 3.16wFPC-Tmem173gt/Nash mice | **Unchanged indicators:** ALT, TG. **Down-regulation:** Lfit1, Lfit3 |  |  |  |  |
| #25 | Cao et al., 2022 | Traditional Chinese medicine Lingguizhugan decoction ameliorate HFD-induced hepatic-lipid deposition in mice by inhibiting STING-mediated inflammation in macrophages | NFALD | 1.HFD-LGZG-H/mice | **Down-regulation:** Oil Red O, Steatosis, Cholesterol, GGT, ALT, Blood Glucose, IPGTT, ITT, 8-OHdG, 4-HNE, 3-NT, mtDNA, STING, F4/80 | 2.HFD-C176/mice | **Down-regulation:** Oil Red O, Steatosis, Cholesterol, GGT, ALT, Blood Glucose | 3.(HFD, DMXAA)-LGZG-H/mice | **Down-regulation:** TNF-α, INF-β, p-TBK1/TBK1, STING | 4.DMXAA-(PA60, CA40, ATR50, GLY250)/BMDMs, Kupffer | **Down-regulation:** Except for PA60 (TNF-α, INF-β). Except for PA60, ATR50 (p-TBK1/TBK1, p-NFκB) | 5.LGZG-(PA-DMXAA-BMDMs)/Hep | **Down-regulation:** Oil-Red-O (PA60, CA40, ATR50, GLY250, MIX40) |

**Supplementary Table 2.** Summary of key findings from studies investigating the role of cGAS-STING pathway in Hepatitis.

| **Item** | **Study** | **Title** | **Disease** | Treatment/Method | Key Findings | Treatment/Method | Key Findings | Treatment/Method | Key Findings | Treatment/Method | Key Findings | Treatment/Method | Key Findings |
| --- | --- | --- | --- | --- | --- | --- | --- | --- | --- | --- | --- | --- | --- |
| #1 | Choi et al., 2020 | A Telomerase-Derived Peptide Exerts an Anti-Hepatitis B Virus Effect via Mitochondrial DNA Stress-Dependent Type I Interferon Production | HBV | 1.GV1001-C2 HBV/HepG2, Huh7, HepG2-2.15 | **Down-regulation:** HBV virion, HBsAg | 2.GV1001-C2 HBV/HepG2 | **Down-regulation:** cccDNA, PgRNA. **Up-regulation:**TNF-α, IFN-β, p-IRF3, p-STAT1, mtDNA, mtROS, 8-OHdG | 3.GV1001+ETV-C2HBV/HepG2-2.15 | **Down-regulation:** cccDNA, PgRNA, HBV virion, HBsAg | 4.GV1001-C2 HBV-STING siRNA/HepG2 | **Up-regulation:** HBsAg, HBeAg, HBV virion |  |  |
| #2 | Guo et al., 2017 | Activation of stimulator of interferon genes in hepatocytes suppresses the replication of hepatitis B virus | HBV | 1.dsDNA90-cGAS-STING/HepAD38 | **Up-regulation:** INF-β, IL-29, CXCL10 | 2.(Tet+/-)-cGAMP/HepAD38,AML12HBV10 | **Unchanged indicators:** HBV, IFN-β, IL-29, IL-28, TNF-α | 3.cGAMP, DMXAA/AML12HBV10 | **Down-regulation:** HBV DNA | 4.Ruxolitinib(JAK inhibitor)-DMXAA, cGAMP/AML12HBV10 | **Up-regulation:** HBV DNA |  |  |
| #3 | Rodriguez-Garcia et al., 2021 | AdrA as a potential immunomodulatory candidate for STING-mediated antiviral therapy that required both type I IFN and TNF - α production | HBV | 1.AdrA-hSTING/HEK293T | **Down-regulation:** IFN-β, c-di-GMP | 2.RAW-Luc-STING2/2/ISG54 | **Down-regulation:** HBcAg | 3.AdrA wt- STING2/2/B6 DC | **Down-regulation:** IL6, IL12, Mcp-1, IFN, CD40, CD86, PD-L1, CD80 | 4.AdrA wt/AAV-HBV | **Down-regulation:** HBV viremia in serum, HBV DNA, HBV RNA. **Unchanged indicators:** HBcAg | 5.3d AdrA wt/AAV-HBV | **Down-regulation:** HBV viremia in serum (B6, IFNAR2/2). **Unchanged indicators:** HBV viremia in serum (STING2/2). |
| #4 | Zhao et al., 2023 | HBV confers innate immune evasion through triggering HAT1/acetylation of H4K5/H4K12/miR-181a-5p or KPNA2/cGAS-STING/IFN-I signaling | HBV | 1.HBV-Huhep-URG/mice | **Down-regulation:** cGAS. **Up-regulation:**HAT1 | 2.HT-DNA-HAT1 KO/HepG2 | **Up-regulation:** cGAS, p-IRF3, p-TBK1, CXCL10 | 3.HDI-HAT1f/f, Alb-cre/mice | **Up-regulation:** cGAS, p-IRF3, p-TBK1, CXCL10 | 4.HBV-HT-DNA-si HAT1/HepG2 | **Up-regulation:** cGAS, IFNb2, CXCL10, cGAMP, IFN-β, p-IRF3, p-TBK1 | 5.HBV-si HAT1/HepG2-NTCP | **Down-regulation:** miR-181A-5P |
|  |  |  |  | 6.HBV-miR-181A-5P/HepG2-NTCP | **Down-regulation:** cGAS | 7.HBV-HAT1 KO/HepG2 | **Down-regulation:** cGAS (Nuclear) | 8.HDI-HAT1 | **Down-regulation:** cGAS (Nuclear) |  |  |  |  |
| #5 | Zheng et al., 2021 | Hbsag dampened sting associated activation of nk cells in hbeag-negative chb patients | HBV | 1.CHB/CD3-CD56+ NKc | **Down-regulation:** STING, IFN-α | 2.Incubating(CHB Serum, HBV-Positive HepG2.1.15)/NK-92 | **Down-regulation:** STING | 3.2'3'-cGAMP-CHB/NKc | **Down-regulation:** CD107a, Granzyme B | 4.shSTAT3/NK-92 | **Down-regulation:** STING | 5.2'3'-cGAMP-shSTAT3/NK-92 | **Down-regulation:** Granzyme B, CD107a, IFN-γ, IRF3, IFNα |
| #6 | Chen et al., 2022 | HBx inhibits DNA sensing signaling pathway via ubiquitination and autophagy of cGAS | HBV | 1.HBx-cGAS STING/HEK293T | **Down-regulation:** IFN-β, Dimerization, p-IRF3, | 2.HBx/SMMC-7721, LO2 | **Down-regulation:** Cgas | 3.HBx-3MA, MG132/SMMC-7721 | **Down-regulation:** cGAS, GFP-LC3. **Up-regulation:** LC3-2. | 4.HBx-cGAS/SMMC-7721 | **Up-regulation:** K48 | 5.HBx-ISD/HBV1.3, HepG2.2.15 | **Down-regulation:** IFN-β, ISG54, ISG56 |
| #7 | Lauterbach-Rivière et al., 2020 | Hepatitis B virus DNA is a substrate for the cGAS/STING pathway but is not sensed in infected hepatocytes | HBV | 1.Sev-HBV DNA/MDDCs | **Up-regulation:** ISG54 | 2.HT-DNA-(STING KO, cGAS KO)/THP-1 KO cell | **Down-regulation:** ISG54, HBV(n.ac.) | 3.1500c/c HBV(n.ac.)-cGAS+STING/HepG2-hNTCP | **Up-regulation:** ISG54 | 4.Over-expressed(cGAS, STING)/HepG2-hNTCP | **Down-regulation:** IRF3 | 5.HT-DNA-Not over-expressed(cGAS, STING)/HepG2-hNTCP | **Down-regulation:** IRF3 |
| #8 | Liu et al., 2015 | Hepatitis B virus polymerase disrupts K63-linked ubiquitination of STING to block innate cytosolic DNA-sensing pathways | HBV | 1.STING/HBV1.3, CMV-HBV | **Down-regulation:** IFN-β, p-55C1B-Luc | 2.cGAMP-STING/HBV1.3, CMV-HBV | **Down-regulation:** IFN-β | 3.HBV1.3-cGAMP-STING/Huh7 | **Down-regulation:** IFN-β | 4.HBV-infected-cGAMP/PHHs | **Down-regulation:** IFN-β | 5.HBV pol-STING/Huh7 | **Down-regulation:** IFN-β |
|  |  |  |  | 6.HBV pol-(STING, PolydA·dT)/(HEK293, 9H5CH8) | **Down-regulation:** IFN-β, ISG56 | 7.STING/Pol, RT/R H, RT, RH | **Down-regulation:** IFN-β | 8.HA-Ub-K63-STING-myc-Pol/293T cells | **Down-regulation:** HA |  |  |  |  |
| #9 | Ding et al., 2013 | Hepatitis C virus NS4B blocks the interaction of STING and TBK1 to evade host innate immunity | HCV | 1.SEV-NS4B/HEK293 | **Down-regulation:** IFN-β, IL28, IL29 | 2.NS4B-IFN-β receptor/HEK293 | **Down-regulation:** RIG-1N, MAVS/VISA, IKK-e, TBK-1, STING/MITA. **Unchanged indicators:** IRF3, p65 | 3.NS4B-STING-myc/HEK293 | **Up-regulation:** STING/MITA(α-Flag) | 4.NS4B-HA-TBK1-Flag-STING/PH5CH8, HEK293 | **Down-regulation:** STING/MITA(α-HA) |  |  |
| #10 | Yi et al., 2016 | Hepatitis C virus NS4B can suppress STING accumulation to evade innate immune responses | HCV | 1.cGAMP/Huh7.5(1b/Con1) | **Down-regulation:** HCV RNA | 2.STING siRNA/Huh7.5(1b/Con1) | **Down-regulation:** 1b/Con1 | 3.NS4Bs(1b/Con1, 2a/JFH1)-STING-Flag/HEK293 | **Up-regulation:** anti-Flag | 4.NS4Bs(2a/JFH1)/293T cells | **Down-regulation:** IFN-β, ISRE, STING-Flag | 5.NS4Bs(2a/JFH1)/HEK293, Huh7.5 | **Down-regulation:** STING, TBK1, IRF3 |
| #11 | Nitta et al., 2013 | Hepatitis C virus NS4B protein targets STING and abrogates RIG-I-mediated type I interferon-dependent innate immunity | HCV | 1.NS4B-(△RIG-1, STING, Poly-dA:dT)/HEK293T cells | **Down-regulation:** IFN-β | 2.NS4B-(△RIG-1, STING, Cardif)/HEK293T cells | **Down-regulation:** p-IRF3 | 3.NS4B-Cardif-Flag/HEK293T Huh7 | **Down-regulation:** HA | 4.STING-siRNA/Huh7-Feo cells | **Up-regulation:** HCV | 5.Overexpressing NS4B/Huh7-Feo cells | **Up-regulation:** HCV |
|  |  |  |  | 6.NS4Btl-84-(△RIG-1, STING, Cardif)/HEK293T, | **Down-regulation:** IFN-β |  |  |  |  |  |  |  |  |
| #12 | He et al., 2016 | Inhibition of hepatitis B virus replication by activation of the cGAS-STING pathway | HBV | 1.cGAS-STING/HepG2-Phbv1.3 | **Down-regulation:** HBV RNA, HBV DNA, HBeAg(LO2). **Up-regulation:** IFN-β(LO2). | 2.HBV-si cGAS/PBMCs | **Down-regulation:** IFN-β. **Up-regulation:** HBV DNA | 3.Phbv1.3-cGAS-STING | **Down-regulation:** HBV DNA, HBV RNA, HBeAg, HBsAg. **Up-regulation:** IFN-β |  |  |  |  |
| #13 | Ono et al., 2014 | Innate immune response induced by baculovirus attenuates transgene expression in mammalian cells | HCV | 1.rBV-GFR-(IRF3-/-, TBK1-/-, IPS1-/-)/MEFs | **Down-regulation:** IFN-β, IP10. **Up-regulation:** GFR | 2.rBV-GFR-(STING-/-)/MEFs | **Down-regulation:** IFN-β. **Up-regulation:** GFR | 3.rBV-GFP-FLag IPS1-/-/MEFs | **Up-regulation:** Transgene expression | 4.shRNA(STING, IPS-1, IRF3)/Huh7 | **Up-regulation:** rBV-Luc | 5.NS3/4A-rBV-BIMs/HCV replicon cells | **Down-regulation:** Cell viability |
| #14 | Thomsen et al., 2016 | Lack of immunological DNA sensing in hepatocytes facilitates hepatitis B virus infection | HBV | 1.AdV-HBV-STING gt/mice (Hepatocytes) | **Unchanged indicators:** HBsAg, pgRNA, IFN-β, ALT. **Up-regulation:** MAVS (compared to spleen). **Down-regulation:** IRF3 (compared to spleen) | 2.AdV-HBV/Kupffer cell | **Down-regulation:** STING, p-TBK-1 | 3.AdV-HBV/Hepatocytes | **Down-regulation:** IFN-β. **Up-regulation:** HBsAg | 4.AdV-HBV-ploy(I:C)/mice | 5.5h HBV-DNA/Liver HDI-STINGgt mice | **Up-regulation:** ISG15 | 6.3Days AdV-HBV/Liver HDI-STINGgt mice(Hepatocytes) |
| #15 | Lin et al., 2021 | Manganese Breaks the Immune Tolerance of HBs-Ag | HBV | 1.1h AAV-HBV-Mncl2/Mouse hepatocytes | **Up-regulation:** HBcAg, (Reach a peak: IFN-α, IFN-β), p-IBK1, p-IRF3 | 2.AAV-HBV-Mncl2-STING-/-/Mouse Hepatocytes, Kupffer cell | **Down-regulation:** IFN-α, IFN-β | 3.AAV-HBV-Mncl2/Kupffer cell | **Up-regulation:** (Reach a peak at 2 hours: IFN-α, IFN-β), ISG15, Osal1. **Down-regulation:** IL6, TNF-α | 4.AAV-HBV-Mncl2-TLR9-/-/Kupffer cell | **Up-regulation:** IFN-α, IFN-β | 5.AAV-HBV-Mncl2/mice | **Down-regulation:** HBsAg. **Up-regulation:** ALT, IFN-α, IFN-β |
|  |  |  |  | 6.rHBVvac-Mncl2/mice | **Up-regulation:** Anti-HBsAg, ALT, IFN-β, CD80, CD86, HBsAg-specific CD8+. **Down-regulation:** HBsAg |  |  |  |  |  |  |  |  |
| #16 | Shu et al., 2023 | RVX-208, an inducer of Apolipoprotein A-I, inhibits the particle production of hepatitis B virus through activation of cGAS-STING pathway | HBV | 1.100μMRVX-208/HepG2.2.15 | **Down-regulation:** HBV DNA(12, 24h), HBeAg(4, 24h), HBsAg(12, 24h). **Up-regulation:** IFN-β, IFN-γ, IL6, IL1b, p-TBK1, p-IRF3, TNF-α, MCP1, MX-1, ISG15 | 2.RVX-208-siApoA-1/HepG2.2.15 | **Up-regulation:** HBV DNA, STING |  |  |  |  |  |  |
| #17 | Zhao et al., 2023 | Schisandrin C enhances cGAS-STING pathway activation and inhibits HBV replication | HBV | 1.HT-DNA-SC/PMA-primed THP-1, L929 | **Up-regulation:** p-IRF3, INF-β | 2.cGAMP-SC/PMA-primed THP-1 | **Up-regulation:** p-IRF3, INF-β, Oligomer | 3.SC-CMA/mice | **Up-regulation:** IFN-β, INF-α, IL6 | 4.20μg pAAV/HBV1.2-30mg SC/mice | **Down-regulation:** HBV DNA(32d), HBsAg(18d), HBeAg(11d, 18d). **Up-regulation:** INF-β, ISG15, IFIT1, CXCL10 |  |  |
| #18 | Guo et al., 2015 | Sting agonists induce an innate antiviral immune response against hepatitis B virus | HBV | 1.DMXAA, CMA, Tilorone/RAW264.7 | **Down-regulation:** RC DNA, DSL DNA | 2.DMXAA/RAW264.7 | **Up-regulation:** IFN-β | 3.Pam3CSK4, Poly 1:C, gardiquimod/RAW264.7 | **Up-regulation:** IL6, IL1, IL10, TNF-α, IL12, CXCL10 | 4.DMXAA-shSTING/RAW264.7 | **Down-regulation:** p-TBK1, IFN-β. **Up-regulation:** HBV DNA | 5.1.3mev HBV-DMXAA/NOD mice | **Down-regulation:** HBV DNA, Viperin. **Up-regulation:** OAS1b |
| #19 | Li et al., 2022 | STING signaling activation inhibits HBV replication and attenuates the severity of liver injury and HBV-induced fibrosis | HBV | 1.DMXAA-prcccDNA/AML12 | **Unchanged indicators:** HBsAg, pgRNA, HBV DNA, rcccDNA. **Up-regulation:** H3K9me3, H3K27me3 | 2.DMXAA-prcccDNA-AML12/RAW264.7 | **Down-regulation:** HBsAg, pgRNA, HBV DNA. **Unchanged indicators:** rcccDNA. **Up-regulation:** p-IRF3, p-TBK1, p-p65 | 3.DMXAA-Ad-rcccDNA/Alb-Cre Tg mice | **Down-regulation:** HBsAg, pgRNA, HBV DNA, α-SMA, Masson area, Sirius red area, Col1a1, MMP2, TIMP1, AST, ALT, IL1b, IL18, p20, p62. **Unchanged indicators:** rcccDNA. **Up-regulation:** IFN-β, TNF-α, p-TBK1, p-p65, STING, p-IRF3, H3K9me3, H3K27me3 | 4.DMXAA-si STING/RAW264.7 | **Up-regulation:** HBsAg, pgRNA, HBV DNA. **Down-regulation:** STING, p-p65, p-IRF3. | 5.DMXAA-LPS+ATP-si STING/KCs | **Up-regulation:** IL1b, p20. **Down-regulation:** LC3-Ⅱ |
|  |  |  |  | 6.DMXAA-LPS+ATP-Leupetin/KCs | **Up-regulation:** p20, IL1b | 7.cGAMP-LPS+ATP-Leupetin/KCs | **Up-regulation:** LC3-Ⅱ, p20, p62, IL1b | 8.DMXAA-Ad-rcccDNA-Leupeptin/Alb-Cre Tg mice | **Up-regulation:** LC3-Ⅱ, p62, p20, IL1b, ALT, AST, α-SMA, Sirius Red area, Masson area, Col1a1, MMP2, TIMP1 |  |  |  |  |
| #20 | Polidarova al., 2023 | Synthetic Stimulator of Interferon Genes (STING) Agonists Induce a Cytokine-Mediated Anti-Hepatitis B Virus Response in Nonparenchymal Liver Cells | HBV | 1.HBV-GS-9620/PHHs | **Down-regulation:** TNF-α, IFN-β, IL6, IL-1b. **Up-regulation:** HBsAg, HBeAg, vRNA, vDNA | 2.prodrug, parent, 2',3'-cGA/hNPCs, PBMCs | **Up-regulation:** IFNα, IFNγ, TNFα, IL6, IL1b | 3.GS-9620, LPS, prodrug, parent, 2',3'-cGA/hNPCs, PBMCs | **Unchanged indicators:** T cells | 4.LPS, 2',3'-cGA/hNPCs, PBMCs | **Up-regulation:** CD4+T |  |  |
| #21 | Wu al., 2024 | The combination of Schisandrin C and Luteolin synergistically attenuates hepatitis B virus infection via repressing HBV replication and promoting cGAS-STING pathway activation in macrophages | HBV | 1.2mg LWWL/HepG2.2.15 | **Down-regulation:** HBeAg. HBsAg, HBV DNA | 2.LWWL+ETV/mice | **Down-regulation:** HBsAg, HBV DNA | 3.(SC-ISD/THP-1)/HepG2.2.15 | **Down-regulation:** HBeAg, HBsAg. **Up-regulation:** p-IRF3 | 4.PAAV/HBV1.2-Lut+SC/mice | **Down-regulation:** HBV DNA(20d), HBeAg(10d), HBsAg(10d). **Unchanged indicators:** HBeAg(20d), HBsAg(20d) |  |  |
| #22 | Dansako al., 2016 | The cyclic GMP-AMP synthetase-STING signaling pathway is required for both the innate immune response against HBV and the suppression of HBV assembly | HBV | 1.p-dAdT, p-dGdc, HBV, VACV, HSV-si cGAS/Li23 | **Down-regulation:** ISG56 | 2.p-dAdT, p-dGdc, HBV, VACV, HSV-si STING/Li23 | **Down-regulation:** ISG56 | 3.p-dAdT, p-dGdc, HBV, VACV, HSV-si IRF3/Li23 | **Down-regulation:** ISG56 | 4.cGAS, STING/HepG2.2.15, HepG2-NTCP | **Up-regulation:** ISG56. **Down-regulation:** pgRNA, HBV transcript, HBV DNA | 5.A8(lowest level of cGAS), B34, B48/Li23-NTCP | **Unchanged indicators:** cGAS, ISG56. **Up-regulation:** HBV transcript |

**Supplementary Table 3.** Summary of key findings from studies investigating the role of cGAS-STING pathway in Chemical and Drug Induced Liver Injury.

| **Item** | **Study** | **Title** | **Disease** | Treatment/Method | Key Findings | Treatment/Method | Key Findings | Treatment/Method | Key Findings | Treatment/Method | Key Findings | Treatment/Method | Key Findings |
| --- | --- | --- | --- | --- | --- | --- | --- | --- | --- | --- | --- | --- | --- |
| #1 | Liu et al., 2023 | ATG2B upregulated in LPS-stimulated BMSCs-derived exosomes attenuates septic liver injury by inhibiting macrophage STING signaling | Septic liver injury | 1.CLP-L-Exo/Septic mice | **Down-regulation:** ALT, AST, TNF-α, IL1b, IL6, TUNEL cells, cGAS, STING, p-TBK1, TNF-α, p62. **Up-regulation:** LC3B, PINK1, parkin | 2. LPS-L-Exo/BMDMs | **Up-regulation:** mitophagosomes | 3.L-Exo/Septic mice, BMDMs | **Up-regulation:** ATG2B | 4.LV-ATG2B-L-Exo/BMSCs | **Up-regulation:** p62. **Down-regulation:** LC3B, PINK1, parkin, ATG2B | 5.CLP-LV-ATG2B-L-Exo/Septic mice | **Up-regulation:** cGAS, STING, p-TBK1, TNF-α, IL1b, IL6. |
| #2 | Zhong et al., 2022 | Defective mitophagy in aged macrophages promotes mitochondrial DNA cytosolic leakage to activate STING signaling during liver sterile inflammation | liver sterile inflammation | 1.HR-Aged/BMDMs | **Up-regulation:** cGAS, p-STING, p-TBK1, p-NFκB, TNF-α, IL6, 2,3-Cgamp | 2.EtBr-Aged/BMDMs | **Down-regulation:** mtDNA, TNF-α, IL6, 2,3-cGAMP | 3.cGAS si RNA-Aged/BMDMs | **Down-regulation:** TNF-α, IL6, 2,3-cGAMP, p-TBK1, p-NFκB | 4.Pink Overexpression-Aged/BMDMs | **Down-regulation:** k48, k63, Mitolysosomes | 5.(Pink1+Torin-1)-Aged/BMDMs | **Up-regulation:** Mitolysosomes. **Down-regulation:** mtDNA, MMP, MitoSOX |
|  |  |  |  | 6.H2O2, TAA, APAP-Aged/BMDMs | **Up-regulation:** cGAS, p-STING, p-TBK1, p-NFκB, TNF-α, IL6, 2,3-cGAMP, mtDNA. **Down-regulation:** Mitolysosomes. | 7.IR, TAA, APAP-STING KO/mice | **Down-regulation:** ALT, AST, p-NFκB, p-TBK1, TNF-α, IL6 |  |  |  |  |  |  |
| #3 | Shen et al., 2022 | Emodin Attenuates Acetaminophen-Induced Hepatotoxicity via the cGAS-STING Pathway | APAP-induced liver injury | 1.APAP-Emo-H/mice | **Down-regulation:** Suzuki's score, ALT, AST, ALP, CYP2E, p-TBK1, p-IRF3, cGAS, STING, IFN-α, TUNEL cells, Bcl2, BAX, IL1b, IL6, IL10, Caspase 1, NLRP3. **Up-regulation:** ALB, GSH, Nrf2, HO-1, NQO1 |  |  |  |  |  |  |  |  |
| #4 | Li et al., 2023 | Ginsenoside Rd Inhibited Ferroptosis to Alleviate CCl4-Induced Acute Liver Injury in Mice via cGAS/STING Pathway | CCl4-Induced Acute Liver Injury | 1.Rd-CCl4/mice | **Down-regulation:** AST, ALT, LDH, Iron, cGAMP, cGAS, STING. **Up-regulation:** T-AOC, GSH | 2.Rd-IKE-CCl4/mice | **Up-regulation:** AST, ALT, LDH, Iron, cGAMP, cGAS, STING. **Down-regulation:** GSH |  |  |  |  |  |  |
| #5 | Yang et al., 2023 | Macrophage PTEN controls STING-induced inflammation and necroptosis through NICD/NRF2 signaling in APAP-induced liver injury | APAP-induced liver injury | 1.APAP-PTEN KO/mice | **Down-regulation:** p-STING, p-TBK1, p-IRF3, p-P65, RIPK3, p-MLKL | 2.APAP-PTEN KO-Notch1 KO/mice | **Down-regulation:** NICK, NRF2. **Up-regulation:** p-STING, p-TBK1, p-P65 | 3.APAP-siRNA Nrf2/mice | **Up-regulation:** AST, ALT, F4/80, Ly6G, Tnf-α, IL1b, IL6, Cxcl-1, Mcp-1, p-STING, p-TBK1, p-IRF3, p-P65 | 4.APAP-PTEN FL/FL-STING KO/mice | **Down-regulation:** INF-β, TNF-α, IL1b, Cxcl-1, Mcp-1, p-TBK1, STING, p-IRF3, p-P65, ROS | 5.APAP-PTEN KO-STING ACT/mice | **Up-regulation:** RIPK3, p-MLKL |
| #6 | Saimaier et al., 2024 | Manganese Exacerbates ConA-Induced Liver Inflammation via the cGAS-STING Signaling Pathway | Autoimmune hepatitis(AIH) | 1.ConA-Mncl2/mice | **Up-regulation:** ALT, AST, TNF-α, IL6, Fas, Fasl, Ifna4, Ifnb1, Mb21dl, Tmem173 | 2.ConA-Mncl2-cGAS-/-/mice | **Down-regulation:** ALT, AST, IL6, TNFα, Ifna4，Ifnb1 |  |  |  |  |  |  |
| #7 | Zhao et al., 2024 | STING modulates iron metabolism to promote liver injury and inflammation in acute immune hepatitis | Autoimmune hepatitis(AIH) | 1.Fer1-ConA/mice | **Down-regulation:** ALT, AST, MDA, STING, 4-HNE. **Up-regulation:** GSH | 2.DFO-ConA/mice | **Down-regulation:** ALT, AST, FTL, MDA, FTH, STING. **Up-regulation:** GSH | 3.ConA-STING-/-/mice | **Down-regulation:** ALT, AST, STING, INF-γ, MDA, TF, TfR, F4-80. **Up-regulation:** GSH | 4.ConA-AAV-STING-CKO/KCs | **Down-regulation:** ALT, AST, TF, FTH, FTL |  |  |
| #8 | Liu et al., 2022 | XBP1 deficiency promotes hepatocyte pyroptosis by impairing mitophagy to activate mtDNA-cGAS-STING signaling in macrophages during acute liver injury | Acute liver injury (ALI) | 1.TAA-HKO/mice | **Up-regulation:** ALT, AST, Necrotic area, Death cells, F4/80, MDA, ROS. **Down-regulation:** GSH/GSSG, SOD, Mitophagosome, LC3-a, LC3-b, p62, parkin, PINK1 | 2.TAA-HKO/BMDMs | **Up-regulation:** STING, mtDNA-Cy5, F4/80, cGAS, STING, p-TBK1, p-IRF3, p-NF-κB | 3.TAA-cGAS-shRNA-HKO/mice | **Down-regulation:** STING, cGAS, p-TBK1, p-IRF3, p-NF-κB, AST, ALT, MCP-1, F4/80, IL-1β, IL18, CXCL-10 | 4.TAA-mtDNA-cGAS-shRNA/mice | **Down-regulation:** cGAS, STING, p-TBK1, p-IRF3, p-NF-κB | 5.TAA-MCC950/mice | **Up-regulation:** NLRP3, C-Caspase-1. GSDMD |
|  |  |  |  | 6.TAA-Mitotempo-HKO/mice | **Down-regulation:** DCFDA, MFI, NLRP3, C-Caspase-1, GSDMD-NT | 7.TAA-CQ-WT/mice | **Up-regulation:** NLRP3, C-Caspase-1, GSDMD-NT, cGAS, STING | 8.TAA-CQ-HKO/mice | **Up-regulation:** LC3-b, p62. **Unchanged indicators:** MDA, GSH/GSSG, SOD, ROS | 9.LPS-HKO/mice | **Up-regulation:** XBPIs, mRNA, AST, ALT, STING |  |  |

**Supplementary Table 4.** Summary of key findings from studies investigating the role of cGAS-STING pathway in HIRI.

| **Item** | **Study** | **Title** | **Disease** | Treatment/Method | Key Findings | Treatment/Method | Key Findings | Treatment/Method | Key Findings | Treatment/Method | Key Findings | Treatment/Method | Key Findings |
| --- | --- | --- | --- | --- | --- | --- | --- | --- | --- | --- | --- | --- | --- |
| #1 | Zhong et al., 2020 | Aging aggravated liver ischemia and reperfusion injury by promoting sting-mediated NLRP3 activation in macrophages | HIRI | 1.IR-Aged/mice | **Up-regulation:** NLRP3, IL1b, IL18, MCP-1, CXCL-10, IL6, TNF-α, Suzuki score, IFN-β, Cleaved caspase-1 | 2.IR-Aged/KCs | **Up-regulation:** NLRP3, IL1b, IL18, MCP-1, CXCL-10 | 3.IR-NLRP3 siRNA-Aged/KCs | **Down-regulation:** Cleaved caspase-1, ALT, AST | 4.IR-NLRP3 siRNA-Aged/mice | **Down-regulation:** NLRP3, IL1b, IL18, MCP-1, CXCL-10, Suzuki score | 5.(HR-Stressed)-Aged(Cell, Sup, mtDNA)/BMDMs | **Up-regulation:** p-STING, p-TBK1, NLRP3, Cleaved caspase-1(except Sup), IL1b, IL18, CXCL-10, MCP-1 |
|  |  |  |  | 6.C-176-Aged/BMDMs | **Down-regulation:** p-STING, p-TBK1, NLRP3, Cleaved caspase-1, IL-1b, IL18, MCP-1, CXCL-10 | 7.IR-STING siRNA-Aged/mice | **Down-regulation:** NLRP3, ALT, AST, Suszuki score, IL1b, IL18, MCP-1, CXCL-10 |  |  |  |  |  |  |
| #2 | Jiao et al., 2022 | Expression of STING Is Increased in Monocyte-Derived Macrophages and Contributes to Liver Inflammation in Hepatic Ischemia-Reperfusion Injury | HIRI | 1.(I/R)/mice | **Up-regulation:** STING mRNA, STING positive cells, STING, STING Gene, STING(CD68+), STING mRNA(macrophages) | 2.C-178, H-151-(I/R)/mice | **Down-regulation:** AST, ALT, Necrotic area, TUNEL, IL6, IL1b, TNF-α, MPO, F4/80, STING, p-TBK1, p-p65, p-IκBα | 3.Lysm-Cre+-(I/R)/mice | **Down-regulation:** AST, ALT, Necrotic area, TUNEL, IL6, IL1b, TNF-α, MPO, F4/80, STING, p-TBK1, p-p65, p-IκBα, C-caspase3, Bad, Bax, HIF-1α. **Up-regulation:** Bcl-2, p-AMPK | 4.mtDNA-STING KO/BMDMs | **Down-regulation:** p-TBK1, p-p65, HIF-1α, IL6, TNF-α, IL1b, HK2, GLUT1, LDHA, PKM2, Lactate excretion. **Up-regulation:** p-AMPK |  |  |
| #3 | Kong et al., 2024 | Inhibition of Sirt3 activates the cGAS-STING pathway to aggravate hepatocyte damage in hepatic ischemia–reperfusion injury mice | HIRI | 1.30/60 min IRI/mice | **Up-regulation:** ALT, AST, LDH, Suzuki score, mtDNA, p-cGAS, p-STING, p-IRF3, TNF-α, IL1b, IFN-β | 2.2h/4h (OGD/R)/mice | **Up-regulation:** LDH, mtDNA, p-cGAS, p-STING, p-IRF3, TNF-α, IL1b, IFN-β | 3.IRI-si cGAS, si STING/mice | **Down-regulation:** TNF-α, IL1b, IFN-β, LDH, Suzuki score, AST, ALT | 4.IRI, ORD/R-Sirt3(-/-)/mice | **Up-regulation:** p-cGAS, p-STING, p-IRF3, TNF-α, IL1b, IFN-β, n-p65 | 5.OGD/R-si cGAS, si STING/mice | **Down-regulation:** TNF-α, IL1b, IFN-β, LDH |
|  |  |  |  | 6.si-p65/AML-12 | **Down-regulation:** cGAS | 7.IRI-Sirt3(-/-)/AML-12 | **Up-regulation:** n-p65 |  |  |  |  |  |  |
| #4 | Shen et al., 2020 | MicroRNA-24-3p alleviates hepatic ischemia and reperfusion injury in mice through the repression of STING signaling | HIRI | 1.I1R9/mice | **Up-regulation:** STING, AST, ALT. **Down-regulation:** miR-24-3p | 2.I/R-si STING/mice | **Down-regulation:** p-IRF3, TNF-α, IL6, ALT, AST | 3.I/R-Ago-miR-24-3p mimic/mice | **Down-regulation:** ALT, AST, TNF-α, IL6, TUNEL, Suzuki's grade, STING, p-IRF3 |  |  |  |  |
| #5 | Zhan et al., 2022 | Novel role of macrophage TXNIP-mediated CYLD–NRF2–OASL1 axis in stress-induced liver inflammation and cell death | HIRI | 1.IR-TXNIP KO/mice | **Down-regulation:** Suzuki score, sALT, sAST, CD116+, Ly6G+, IL6, TNF-α, CXCL-10, MCP-1, IFN-β, p-TBK1, p-IRF3, p-IκBα, p-p65 | 2.IR/mice | **Up-regulation:** TXNIP, p-STING, p-TBK1 | 3.IR/Kupffer cell | **Up-regulation:** CD68+, p-STING | 4.IR-TXNIP KO/Kupffer cell | **Down-regulation:** p-TBK1, p-IRF3, p-p65 |  |  |
| #6 | Peng et al., 2024 | PPM1G regulates hepatic ischemia/reperfusion injury through STING-mediated inflammatory pathways in macrophages | HIRI | 1.H/R-plasmind/RAW264.7 | **Up-regulation:** PPM1G. **Down-regulation:** p-STING, p-IRF3, p-p65, TNF-α, IL6 | 2.H/R-LV/RAW264.7 | **Down-regulation:** PPM1G, CD206. **Up-regulation:** p-STING, P-TBK1, p-IRF3, p-IRF7, p-p65, TNF-α, IL6, INOS | 3.H/R-LV/mice | **Up-regulation:** Suzuki score, ALT, AST | 4.H/R-LV-C-176/RAW264.7 | **Down-regulation:** p-STING, p-TBK1, p-IRF3, p-IRF7, p-p65, p-p38, p-JNK1/2/3, F4/80, TNF-α, IL6, INOS, CD86. **Up-regulation:** IL10, CD206, Arg-1 | 5.H/R-LV-C-176/mice | **Down-regulation:** Suzuki score, ALT, AST |
| #7 | Wu et al., 2022 | STING Induces Liver Ischemia-Reperfusion Injury by Promoting Calcium-Dependent Caspase 1-GSDMD Processing in Macrophages | HIRI | 1.H/R-BAPTA-AM/KCs | **Down-regulation:** 480/520 nm, GSDMD, GSDMD-H, Caspase1, LDH, IL1b, IL18 | 2.IRI-RNAi STING/mice | **Down-regulation:** AST, ALT, STING, Suzuki socre | 3.H/R-si RNA STING/KCs | **Down-regulation:** STING, Caspase1, GSDMD, GSDMD-N, LDH, IL1b, IL18 | 4.IRI-VX765/KCs | **Down-regulation:** Caspase1, cleaved-caspase1, GASDMD, GSDMD-N, LDH | 5.IRI-VX765/mice | **Down-regulation:** AST, ALT, IL1b, Suzuki score |

**Supplementary Table 5.** Summary of key findings from studies investigating the role of cGAS-STING pathway in Liver Cirrhosis.

| **Item** | **Study** | **Title** | **Disease** | Treatment/Method | Key Findings | Treatment/Method | Key Findings | Treatment/Method | Key Findings | Treatment/Method | Key Findings | Treatment/Method | Key Findings |
| --- | --- | --- | --- | --- | --- | --- | --- | --- | --- | --- | --- | --- | --- |
| #1 | Shen et al., 2022 | Accumulation of polystyrene microplastics induces liver fibrosis by activating cGAS/STING pathway | Liver Cirrhosis | 1.1mg/L micro-PS/mice | **Down-regulation:** PPARα, PGC-1α, MFN-1, ND1, UQCRC2. **Up-regulation:** DRP-1, mt-Cyb, IL1b, IL6, TNF-α, α-SMA, cGAS, STING, p-NFκB | 2.C176-PS/mice | **Down-regulation:** STING, p-NFκB, α-SMA, Fibronecitin, |  |  |  |  |  |  |
| #2 | Luo et al., 2023 | Activation of cGAS-STING signaling pathway promotes liver fibrosis and hepatic sinusoidal microthrombosis | Liver Cirrhosis | 1.CCL4-cGAS-KO/mice | **Down-regulation:** Ishak score, α-SMA, Collagen area, STING, TBK1, IRF3, IL1b, IL6, CXCL-1, IFN-β | 2.cGAS-si RNA, STING-si RNA-TGF-β1/LX-2 | **Down-regulation:** Col-1a1, α-SMA, cGAS, STING, TBK1, IRF3, IL1b, IL6, CXCL-1, IFN-β |  |  |  |  |  |  |
| #3 | Iracheta-Vellve et al., 2016 | Endoplasmic reticulum stress-induced hepatocellular death pathways mediate liver injury and fibrosis via stimulator of interferon genes | Liver Cirrhosis | 1.CCL4-IRF3 KO/mice | **Down-regulation:** ALT, TUNEL, Sirius-red, α-SMA, Acta2, Col1a2, Ifnb1, ISG15, Cleaved Caspase-3 | 2.CCL4-BX 795/mice | **Down-regulation:** p-IRF3, ALT, Cleaved Caspase-3 | 3.CCL4-Tmem173 gt/mice | **Down-regulation: A**LT, Sirius-red, α-SMA, Acta2, Col1a2, ISG15 | 4.CCL4/HEP | **Up-regulation:** p-IRF3, Caspase-3, caspase-8 |  |  |
| #4 | Wu et al., 2024 | IRF3 activates RB to authorize cGAS-STING-induced senescence and mitigate liver fibrosis | Liver Cirrhosis | 1.CCL4-IRF3(-/-)/mice | **Down-regulation:** SA-β-Gal. **Up-regulation:** Sirius-Red, Col1a1, Col3a1 | 2.BDL-IRF3(-/-)-Palbociclib/mice | **Up-regulation:** SA-β-Gal, P21(HSC). **Down-regulation:** Sirius-Red, ALT, AST | 3.CCL4-IRF3(-/-)/NPCs | **Down-regulation:** p16(INK4a), p21(Cip1/Waf1), IL6, CXCL1. **Up-regulation:** cGAS, STING | 4.CCL4-IRF3 Cre/HSCs | **Down-regulation:** IL6, p21. **Up-regulation:** SA-β-Gal, Sirius-Red, Col1a, Col3a1, α-SMA. **Unchanged indicator:** INF-α, IL1b |  |  |
| #5 | Shan et al., 2023 | Mitochondrial oxidative stress regulates LonP1-TDP-43 pathway and rises mitochondrial damage in carbon tetrachloride-induced liver fibrosis | Liver Cirrhosis | 1.CCL4-MitoQ/mice | **Up-regulation:** DHE, Nrf2, Hsp60, eif2α, ATF5(Nuclear), LonP1. **Down-regulation:** TDP-43, mtDNA, F4/80, AST, ALT, α-SMA, Collagen 1 |  |  |  |  |  |  |  |  |
| #6 | Sun et al., 2023 | Oroxylin A activates ferritinophagy to induce hepatic stellate cell senescence against hepatic fibrosis by regulating cGAS-STING pathway | Liver Cirrhosis | 1.CCL4-40μM OA/mice | **Down-regulation:** α-SMA, Fibronectin, Collagen 1 | 2.40μM OA/HSC-LX2 | **Up-regulation:** NCOA4, Beclin1, ROS, Iron, IL1b, IL6, IFN-β. **Down-regulation:** FTH, p62 | 3.40μM OA-si cGAS/HSC-LX2 | **Down-regulation:** SA-β-Gal, IL1b, IL6, IFN-β, NCOA4, LC3-b, Beclin1, ROS, Iron. **Up-regulation:** p16, p21, Cyclin D1, CyclinE1, CDK4, CDK6, TERT, α-SMA, Collagen1, FTH1 | 4.40μM OA-INF-β/HSC-LX2 | **Up-regulation:** NCOA4. **Down-regulation:** FTH1 |  |  |
| #7 | Zhao et al., 2023 | Oroxylin A regulates cGAS DNA hypermethylation induced by methionine metabolism to promote HSC senescence | Liver Cirrhosis | 1.30μM OA/HSC-LX2 | **Down-regulation:** α-SMA, Collagen1, TERT, TRF1, TRF2, CDK4, CDK6, CyclinD1, CyclinE1. **Up-regulation:** HMGA1, p16, p21, cGAS, STING, p-TBK1, p-IRF3, cGAMP, IFN-β, IL1b, IL6, IL8, CXCL9, CXCL10, CXCL11, ISG15 | 2.30μM OA-si cGAS/HSC-LX2 | **Down-regulation:** STING, p-TBK1, p-IRF3, p16, p21, CDK4, CDK6, CyclinD1, CycinE1, DNMT3A. **Up-regulation:** HMGA1, TRF1, TRF2, TERT | 3.30μM OA-si cGAS/HSC-LX2 | **Down-regulation:** 5-mc, cGAS, STING, p-TBK1, p-IRF3, cGAMP, IFN-β, IL1b, IL6, IL8, ISGs | 4.30μM OA-SAMe/HSC-LX2 | **Up-regulation:** DNMT3A. **Down-regulation:** p-IRF3, p-TBK1, STING, cGAS | 5.30μM VA-Lip-DNMT3A/mice | **Up-regulation:** LV-C, PC-Ⅲ, HA, LN, TBIL, CG, AST, ALT, ALP, α-SMA, Collagen1 |
| #8 | Wang et al., 2022 | Pharmacological targeting of cGAS/STING-YAP axis suppresses pathological angiogenesis and ameliorates organ fibrosis | Liver Cirrhosis | 1.CCL4, UUO-cGAS(-/-)/mice | **Up-regulation:** Sirius-Red, α-SMA, TAZ, YAP, Collagen1, ALT, AST | 2.CCL4-SR-717/mice | **Down-regulation:** α-SMA, Collagen1, ALT, AST, FSP, TAZ | 3.PBS-SR-717/HOVECs | **Down-regulation:** Ki67, YAP, CyclinD1, CyclinD2, CyclinD3, p-YAP, p-TAZ | 4.CCL4, UUO-YAP KO/mice | **Down-regulation:** Collagen1, α-SMA |  |  |
| #9 | Gu et al., 2024 | Senescence of Hepatic Stellate Cells by Specific Delivery of Manganese for Limiting Liver Fibrosis | Liver Cirrhosis | 1.Mncl2/HSC-LX2 | **Up-regulation:** SA-β-Gal, p21, p16, IL6, IL8, IFN-b1, γ-H2AX. **Down-regulation:** α-SMA, Col1 | 2.H-151-Mncl2-TGF-β/LX2 | **Down-regulation:** p21, p16, p-IRF3, p-TBK1, p-STING. **Up-regulation:** MICA | 3.CCL4-HDMn/mice | **Up-regulation:** CD314+, CD107a, granzymeB. **Down-regulation:** α-SMA, ALT, AST | 4.Mn@ALB/HSC-LX2 | **Up-regulation:** SA-β-Gal, p21, p16, IFNB1 |  |  |
| #10 | Wu et al., 2023 | Site-specific ubiquitination of VDAC1 restricts its oligomerization and mitochondrial DNA release in liver fibrosis | Liver Cirrhosis | 1.CCL4-Parkin(-/-)/mice | **Up-regulation:** Col1a1, Acta2, Mmp2, Masson area, Sirius-Red, ALT, AST, p62, TUNEL, BAX, Bcl2, Caspase3, mtDNA, cGAS, p-STING, p-IRF3, p-NFκB, IFN-β, TNF-α, IL6. **Down-regulation:** Atg5, Atg7, LC3b/LC3a | 2.si Endog-Parkin(+/+)/HepG2 | **Unchanged indicator:** Pearson correlation coefficient (dsDNA/mitochondria) | 3.si Endog-Parkin(+/+)-Mdivi-1/HepG2 | **Down-regulation:** mt-Nd1, mt-Nd6 | 4.si Endog-(VDAC1-K53R)/LX2 | **Up-regulation:** p-STING, p-IRF3, IL6, IFNb1 | 5.si Endog-(Parkin-CS)/LX2 | **Up-regulation:** p-STING, p-IRF3, IL6, IFNb1 |
| #11 | Wang et al., 2022 | XBP1-mediated activation of the STING signalling pathway in macrophages contributes to liver fibrosis progression | Liver Cirrhosis | 1.(BDL, CCL4, MCD)-Xbp1 KO/mice | **Down-regulation:** SR, α-SMA, NLRP3, STING, p-IRF3 | 2.LPS-(ATP)-Xbp1 KO/BMDMs | **Down-regulation:** NLRP3, Caspase-1, IL1b, cGAS, STING, p-TBK1, p-IRF3,Tnfa, IL6, CXCL10, mt-Cytb, mt-ND4, D-loop, p62. **Up-regulation:** LC3b, BNIP3 | 3.EtBr-Xbp1 KO/BMDMs | **Down-regulation:** D-loop, cGAS, STING, p-TBK1, p-IRF3, NLRP3 | 4. LPS-BNIP3 siRNA-Xbp1 KO/BMDMs | **Down-regulation:** LC3b. **Up-regulation:** p62, D-loop, α-SMA, SR | 5.DMXAA-Xbp1 KO-TGF-β1/HSC-LX2 | **Up-regulation:** Acta2, Col1a1, Timp1 |
|  |  |  |  | 6.(BDL, CCL4, MCD)-Toyocamycin/mice | **Down-regulation:** Timp1, Col1a1, Acta2, SR, α-SMA |  |  |  |  |  |  |  |  |
| #12 | Xiao et al., 2023 | STING mediates hepatocyte pyroptosis in liver fibrosis by Epigenetically activating the NLRP3 inflammasome | Liver Cirrhosis | 1.CCL4-STING(-/-)/mice | **Down-regulation:** α-SMA, Collagen1, Sirius-red | 2.CCL4-C-176/mice | **Down-regulation:** α-SMA, Collagen1, Sirius-red, MPO, F4/80, CYP2E1 | 3.TNF-α-DMXAA-C-176/mice | **Down-regulation:** NLRP3, STING, cl-Caspase1, cl-GSDMD, p-IRF3, IL-18 | 4.TAA-Nlrp3(△HEP)/mice | **Down-regulation:** Collagen1, α-SMA, AST, ALT, MPO, F4/80, IL1β, TNF-α, IFN-γ, IL-6, CCL-5 | 5.CCL4-MCC950/mice | **Down-regulation:** Collagen1, α-SMA. **Up-regulation:** CYP2E1 |

**Supplementary Table 6.** Summary of key findings from studies investigating the role of cGAS-STING pathway in Liver Neoplasms.

| **Item** | **Study** | **Title** | **Disease** | Treatment/Method | Key Findings | Treatment/Method | Key Findings | Treatment/Method | Key Findings | Treatment/Method | Key Findings | Treatment/Method | Key Findings |
| --- | --- | --- | --- | --- | --- | --- | --- | --- | --- | --- | --- | --- | --- |
| #1 | Chen et al., 2024 | A DNA/DMXAA/Metal-Organic Framework Activator of Innate Immunity for Boosting Anticancer Immunity | Liver Neoplasms | 1.MOF-CpG-DMXAA/RAW264.7, BMDMs | **Up-regulation:** CD86+, CD80+, iNOS, TNF-α, IL6. **Down-regulation:** Arg1 | 2.IL4-MOF-CpG-DMXAA/RAW264.7, BMDMs | **Up-regulation:** CD86+CD-, CD80, IL6. **Down-regulation:** CD86-CD206+ | 3.MOF-CpG-DMXAA/BMDCs | **Up-regulation:** MHC2+CD86+, CD80+, MHC2, IL6, NF-Κb-p65, cGAS, p-STING, STING | 4.Hepa1-6 cells co-cultured with MOF-CpG-DMXAA/BMDCs | **Up-regulation:** CD11c, MHC2 | 5.MOF-CpG-DMXAA/HCC-bearing mice | (Cure rate: 80%), (Tumor recurrence rate: 0%). **Down-regulation:** Liver weight. **Up-regulation:** IL6, TNF-α, iNOS, CD3-CD49+ |
|  |  |  |  | 6.MOF-CpG-DMXAA/F4/80+cells | **Up-regulation:** CD80+CD86+ | 7.MOF-CpG-DMXAA/HCC | **Down-regulation:** CD25+FOXP3+. **Up-regulation:** CD4+CD8+, IL17 | 8.MOF-CpG-DMXAA/MH2+CD11c+cells | **Up-regulation:** CD80+CD86+ |  |  |  |  |
| #2 | Sun et al., 2023 | A recombinant oncolytic influenza virus expressing a PD-L1 antibody induces CD8+ T-cell activation via the cGas-STING pathway in mice with hepatocellular carcinoma | Liver Neoplasms | 1.96h(0.1, 1, 3, 5MOI rgFlu/PD-L1)/HepG2, SMMC-7721, MHCC-97L, Huh-7 | **Down-regulation:** HCC survival rate | 2.rgFlu/PD-L1/PDX mice | **Down-regulation:** Tumor volume, Tumor weight. **Up-regulation:** CD8+, APC co-inhibition, Dcs, T cell co-stimulation, Th1, STING, p-STING, IRF3, TBK1 | 3.rgFlu/PD-L1/HepG2 | **Up-regulation:** Apoptotic cells |  |  |  |  |
| #3 | Sheng et al., 2020 | ATR inhibitor AZD6738 enhances the antitumor activity of radiotherapy and immune checkpoint inhibitors by potentiating the tumor immune microenvironment in hepatocellular carcinoma | Liver Neoplasms | 1.IR-AZD6738/mice(Hepa1-6 cells) | **Up-regulation:** CD8+ T cells(64%, D14, Tumor), CD8+ IFN-γ+(D8, D14, Tumor). **Down-regulation:** PD-1(MFI, D8), Tim3(MFI, D14), Treg(D8, Tumor), TIL PD-1+(D8, Tumor) | 2.IR-AZD6738-Anti-PD-L1/mice(Hepa1-6 cells) | **Up-regulation:** TIL CD8+(D14, Tumor), CD8+/CD3+(D14,Tumor), CD8+/Treg(D14, Tumor and spleen), TIL CD4+Ki67+(D8, Tumor), TIL CD8+Ki67+(D8, Tumor), TIL IFN-γ+(D8, D14, Tumor), TIL IFN-γ+(D14, Spleen), cGAS, p-STING, p-TBK1, TIL CD8+Tcm(D8, D14, Tumor), TIL CD8+ Tem(D14, Tumor). **Down-regulation:** TIL Tregs(D8, Tumor), TIL PD-1+LAG3+(D8, D14, Tumor), PD-1+Tim3+(D8, D14, Tumor), Tumor volume. | 3.IR-AZD6738-Anti-PD-L1-C-176/mice(Hepa1-6 cells) | **Up-regulation:** Tumor volume |  |  |  |  |
| #4 | Zhang et al., 2021 | Cancer cell-intrinsic STING is associated with CD8 + T-cell infiltration and might serve as a potential immunotherapeutic target in hepatocellular carcinoma | Liver Neoplasms | 1.STING-high/HCC | **Up-regulation:** CD8+, IFN-β, CXCL-10 | 2.STING-low/HCC | **Down-regulation:** CD8+(20%) | 3.cAIMP/mice(Hepa1-6) | **Up-regulation:** CD8+. **Down-regulation:** Tumor volume, metastastic nodes of lung |  |  |  |  |
| #5 | Du et al., 2021 | DNA sensing and associated type 1 interferon signaling contributes to progression of radiation-induced liver injury | Liver Neoplasms | 1.IR-cGAS-/-, STING-/-/mice | **Down-regulation:** Steatosis score, Histology score, TUNEL, ALT, AST | 2.IR/Hep | **Up-regulation:** dsDNA, CXCL-10, IFIT1, Viperin, cGAS, STING, TLR9 | 3.IR/NPCs | **Up-regulation:** cGAS, STING, TLR9, IFNα, IFN-β | 4.RT-DNase/mice | **Down-regulation:** cGAS, STING, TLR9, IFNa4, IFNβ, CXCL-10, IFIT1, Viperin | 5.IR-DNase/mice | **Down-regulation:** ALT |
|  |  |  |  | 6.RT-rIFNα/mice | **Down-regulation:** ROS | 7.IR-IRFAR-/-/mice | **Down-regulation:** 8oxoG/Dg, ALT, AST, TUNEL, Steatosis, score, CXCL-10, IFIT1, Viperin, Tumor volume |  |  |  |  |  |  |
| #6 | Xu et al., 2024 | Enhanced NK cell activation via eEF2K-mediated potentiation of the cGAS–STING pathway in hepatocellular carcinoma | Liver Neoplasms | 1.Eef2k KO-DCs(co-culturing)/HepG2 | **Up-regulation:** p-STING, p-IRF3, p-TBK1, CXCL-9, IL2 | 2.C-176-Eef2k KO-DCs(co-culturing)/HepG2 | **Down-regulation:** CXCL-9, IL2 | 3.siRNA Eef2k/NK 92 | **Down-regulation:** NK cells | 4.NH125-PD-1-C-176/mice(Hepa 1-6) | **Down-regulation:** NK cells, NKG2A, Granzyme B, IFN-γ, TNF-α. **Up-regulation:** NKG2AB6 |  |  |
| #7 | Li et al., 2022 | Gut microbiota modulate radiotherapy-associated antitumor immune responses against hepatocellular carcinoma Via STING signaling | Liver Neoplasms | 1.RT-antibiotic-H22/ABX mice | **Down-regulation:** Tumor volume, caspase3, PARP, BAX, CD8+/IFN-γ, CD45/CD3+, IFN-γ, ISG-7, CD11c, p-STING, p-p65, p-TBK1, IRF3 | 2.STING(-/-), Cgas(-/-)-RT-antibiotic-H22/ABX mice | **Unchanged indicators:** Tumor volume, IFN-β, CD8/IFN-γ, IFN-γ | 3.STING(-/-), Cgas(-/-)-NR-IR-c-di-AMP/H22 mice | **Up-regulation:** Tumor volume | 4.IR-c-di-AMP/DCs | **Up-regulation:** IFN-β, ISG, CD86, CCR1, CCR2, p-p65, p-IRF3, p-TBK1, p-STING | 5.cGAS(-/-), STING(-/-)-IR-c-di-AMP/BMDCs | **Unchanged indicators:** IFN-γ |
| #8 | Song et al., 2024 | Harnessing a triphenylphosphine-based AIE nano-platform for triggering incomplete mitophagy to continuously augment anti-tumor immune response in hepatocellular carcinoma | Liver Neoplasms | 1.AIE-Mito-TPP/Hepa 1-6 cells | **Up-regulation:** mtDNA(Dloop, Co1), VDAC1, TOM20, H3, PINK1, Parkin, LC3-b, SQSTM1/p62, p-IRF3, p-STING, p-TBK1, Ifnbb1, CXCL-10 | 2.AIE-Mito-TPP/BMDCs | **Up-regulation:** p-IRF3, p-STING, p-TBK1, Ifnbb1, CXCL-10, F4/80+CD86+, TNF-α, mature DCs, CD80+CD86+, CD40+MHC-b+. **Down-regulation:** F4/80+CD206+ | 3.si-Atg5, 3MA-AIE-Mito-TPP/Hepa1-6 cells | **Down-regulation:** p-IRF3, p-TBK1 | 4.DPPA-1M@AIE-Mito-TPP/(Hepa1-6)mice | **Down-regulation:** Liver weight, CD11b+Cr-1+, CD25+FOXP3+. **Up-regulation:** percent survival, CD86+/CD206+, CD11c+MHC-2+, CD3+CD8+, CD69+CD8+, CD4+CD8+, CD3+CD4+, CD69+CD4+, CD44+CD4+, CD80+CD86+, IFN-γ, IFN-β, STING |  |  |
| #9 | Huang et al., 2020 | Highly efficient and tumor-selective nanoparticles for dual-targeted immunogene therapy against cancer | Liver Neoplasms | 1.TT-LDCP/HCA-1, Hep3B | **Up-regulation:** pDNA, siRNA, IL-2, CD84, p-TBK1, p-IRF3, Ifnb, Ccl5, Cxcl-10. **Down-regulation:** PD-L1, Tumor volume. **Unchanged indicator:** CD4+ | 2.(pDNA/siRNA)-TT-LDCP/mice | **Up-regulation:** CD86+, MHC-2+ |  |  |  |  |  |  |
| #10 | Li et al., 2022 | Hyperbaric oxygen facilitates teniposide-induced cGAS-STING activation to enhance the antitumor efficacy of PD-1 antibody in HCC | Liver Neoplasms | 1.Teniposide/Hep3B, Huh7 cells | **Up-regulation:** IFN-β, IFIT-1, IFIT-2, CCL5, CXCL-10 | 2.Teniposide-sh-cGAS, sh-STING/Hep3B, Huh7 | **Down-regulation:** p-p65, p-IRF3 | 3.1%O2/Hep3B, Huh7 | **Up-regulation:** HIF-1α. **Down-regulation:** cGAS | 4.HT-DNA-1%O2/Hep3B, Huh7 | **Up-regulation:** HIF-1α. **Down-regulation:** p-IRF3 | 5.Teniposide-1%O2/Hep3B, Huh7 | **Down-regulation:** p-p65, p-IRF3, IFN-β, IFIT-1, IFIT-2, CCL5, CXCL-10. **Up-regulation:** HIF-1α |
|  |  |  |  | 6.8h Reoxygenation/Hep3B, Huh7 | **Up-regulation:** cGAS | 7.Teniposide-Re-O2/Hep3B, Huh7 | **Up-regulation:** p-p65, GAS, p-IRF3, CCL5, CXCL-10, IFN-β | 8.shHIF-1A-1%O2/Hep3B, Huh7 | **Up-regulation:** CCL5, CXCL-10, IFN-β | 9.HBO-Teniposide/mice | **Up-regulation:** Ifn-b, CCL5, CXCL-10, Macrophages, M1, CD86, MH-1, MHC-2, CD8+, IFN-γ, TNF-α, GZMB, Ki67, CD11c. **Down-regulation:** M2 | 10..HBO-Teniposide+PD1/(Hepa1-6)mice | **Down-regulation:** Tumor volume |
| #11 | Zhao et al., 2022 | Hypoxia-induced RNASEH2A limits activation of cGAS-STING signaling in HCC and predicts poor prognosis | Liver Neoplasms | 1.Hypoxia, CoCl2/Hep1, HepG2 | **Up-regulation:** RNASEH2A | 2.Hypoxia-siHIF2α, siHIF1α/Hep1, HepG2 | **Down-regulation:** RNASEH2A | 3.Hypoxia-SgRNASEH2A/Hep1, HpG2 | **Up-regulation:** USP18, CXCL-10, IRF7, IFIT1, IFI44, IFIT3, CCL5, ISG15 |  |  |  |  |
| #12 | Wu et al., 2023 | Immune checkpoint therapy-elicited sialylation of IgG antibodies impairs antitumorigenic type I interferon responses in hepatocellular carcinoma | Liver Neoplasms | 1.Is9otype-αPD-L1/(Hepa106)mice | **Up-regulation:** STA6GAL1 | 2.STA6GLA1 KO/(Hepa1-6)mice | **Down-regulation:** IgG | 3.IFNG-αPD-L1/(Hepa1-6)mice | **Up-regulation:** ST6GAL1 | 4.Sia-IgG, sia-FC, ST6GAL1/(Hepa1-6)mice | **Up-regulation:** Tumor size | 5.si IL10RA/(Hepa1-6)mice | **Down-regulation:** IL10RA, CD209B |
|  |  |  |  | 6.si IL10RA-αPD-L1/(Hepa106)mice | **Up-regulation:** Flux(hepatoma growth) | 7.sia IgG-DC-SIGN/Necrotic cell | **Down-regulation:** IFNB1, IFN-β | 8.sia-IgG-si CD209/Necrotic cell | **Up-regulation:** STING, IRF3, IFNB1 | 9.si ATF3-DC-SIGN+/macrophages | **Up-regulation:** STING, IRF3 | 10.si CD209-sia-IgG/macrophage | **Down-regulation:** ATF3, p-Raf-1 |
|  |  |  |  | 11.GW5074-sia-IgG/macrophages | **Down-regulation:** ATF3. **Up-regulation:** STING, IRF3 | 12.αPD-L1-ST6GAL1 KO/(Hepa1-6)mice | **Up-regulation:** CD8+, CTL, IFNB1. **Down-regulation:** IL10, CD209B | 13.αPD-L1-3Fax-PN KO/(Hepa1-6)mice | **Up-regulation:** IFNB1, Flux(tumor growth), NKp46 |  |  |  |  |
| #13 | Chan et al., 2023 | Inhibition of CAF-1 histone chaperone complex triggers cytosolic DNA and dsRNA sensing pathways and induces intrinsic immunity of hepatocellular carcinoma | Liver Neoplasms | 1.DEN-ccl4/mice | **Up-regulation:** Chaf1a, Chaf1b | 2.Tp53 KO, c-Myc OE/(Hepa1-6)mice | **Up-regulation:** Chaf1a, Chaf1b | 3.CAF-1 KO/MHCC97L, Huh7 | **Up-regulation:** Hpoptosis rate, RPA32-pTHr21, Γh2ax, p53, SA-β-Gal, IFNA, IFNB, IFI27, ISG15, IFITM1, TRIF, IRF7, CCL2, IL1A, IL1B, IL6, cGAS, TRIF, p65, p-STING, STING Dinner, IRF3, IRF7, micronuclei, p21, CDKN1A, H3.3, J2 dsRNA. **Down-regulation:** H3.1, HCC | 4.myc-OE, Tp53(-/-)- CAF-1 KO/mice | **Down-regulation:** Liver weight. **Up-regulation:** Percent survival | 5.si STING, si MAVS, siTRIF-CAF-1 KO/(Hepa1-6)mice | **Down-regulation:** CCL2, IFNB |
|  |  |  |  | 6.shChaf1b/(Hepa1-6)mice | **Down-regulation:** Tumor weight, Tumor volumn. **Up-regulation:** CD3+. CD4+, CD8+, NKT cell, B cell, GZM+ | 7.Anti-PD1-shChaf1b/(Hep1-6)mice | **Down-regulation:** Tumor volume. **Up-regulation:** CD8+ |  |  |  |  |  |  |
| #14 | Lasarte-Cia et al., 2021 | Intratumoral STING Agonist Injection Combined with Irreversible Electroporation Delays Tumor Growth in a Model of Hepatocarcinoma | Liver Neoplasms | 1.IRE-c-di-GMP/(PM299L)mice | **Down-regulation:** Tumor area. **Up-regulation:** Percent survival, CD45, CD44high/CD4, CD44high/CD8, TNF-αIFN-γ/CD8CD4high， TNF-αIFN-γ/CD4CD44high, IFN-γ/NKp46 |  |  |  |  |  |  |  |  |
| #15 | Li et al., 2024 | NRF2 mutation enhances the immune escape of hepatocellular carcinoma by reducing STING activation | Liver Neoplasms | 1.Nfe2I2 T80K OE/(Hepa1-6)mice | **Up-regulation:** Tumor volume. **Down-regulation:** survival, immune cells, CD8+, NK cells | 2.Nfe2I2 T80K OE-ADU-S100/Hepa1-6, HepG2 cells | **Down-regulation:** STING, Ifn-β, CXCL-10, ISG15, Das | 3.Nfe2I2 T80K-TMEM173 OE/Hepa1-6 cells | **Up-regulation:** STING, Ifn-β, CXCL-10, ISG15, Das, Survival, immune cells, CD8T, NK cells. **Down-regulation:** Tumor volume | 4.ADU-S100-Brusatol/(Hepa1-6)mice | **Down-regulation:** Tumor volume. **Up-regulation:** Survival, immune cells, CD8+, NK cells |  |  |
| #16 | Chen et al., 2024 | Olaparib enhances radiation-induced systemic anti-tumor effects via activating STING-chemokine signaling in hepatocellular carcinoma | Liver Neoplasms | 1.IR-Olaparib/Huh7, SNU-449 cells | **Down-regulation:** Colony number. **Up-regulation:** γH2AX, Tail moment | 2.IR-Olaparib/Huh7 mice | **Up-regulation:** γH2AX, C-CASP3, ATP, HMGB1, p-IRF3, p-TBK1, p-STING, cGAS, IFN-β, CD3+, CD8+, IFN-γ, CXCL-9, CXCL-10, CXCL-11, CCL5. **Down-regulation:** Tumor volume, Ki67. **Unchanged indicator:** CD4+ | 3.H151-IR-Olaparib/Huh7 mice | **Down-regulation:** CXCL-9, CXCL-10, CXCL-11, CCL5 |  |  |  |  |
| #17 | Du et al., 2022 | Radiation Therapy Promotes Hepatocellular Carcinoma Immune Cloaking via PD-L1 Upregulation Induced by cGAS-STING Activation | Liver Neoplasms | 1.12Gy IR/Huh7, HCCLM3, MHCC97H, H22, Hepa1-6 | **Up-regulation:** PD-L1 | 2.24hRT/H22 cells | **Up-regulation:** TMEM173, TBK1, IRF3 | 3.12Gy RT/H22, Huh7, HCCLM3 | **Up-regulation:** cGAS, p-STING, p-TBK1, p-IRF3 | 4.IR-siSTING, siTBK1, siIRF3/Huh7, HCCLM3 | **Down-regulation:** PD-L1 | 5.IR-si cGAS/H22 mice | **Down-regulation:** PD-L1 |
|  |  |  |  | 6.IR-cGAS WT-Anti-PD-L1/H22 mice | **Down-regulation:** Tumor volume. **Up-regulation:** CD8+, Granzyme+ | 7.IR-Anti-PD-L1/H22 mice | **Down-regulation:** Tumor volume. **Up-regulation:** CD3+, CD8+, CD8+IFNγ+, GranzymeB+, CD69+ | 8.IR-Anti-PD-L1/Hepa1-6 cells | **Up-regulation:** CD44 high CD62L low, CD8+, SINFEKL, INF-γ. **Down-regulation:** Tumor volume, CD44 medium CD62L high |  |  |  |  |
| #18 | Hong et al., 2024 | RECQL4 Inhibits Radiation-Induced Tumor Immune Awakening via Suppressing the cGAS-STING Pathway in Hepatocellular Carcinoma | Liver Neoplasms | 1.6Gy IR-sh-RECQL4/MHCC97H cells | **Up-regulation:** γ-H2AX, dsDNA, **Down-regulation:** Survival and profieration | 2.RT-RECQL4 OE/H22 mice | **Up-regulation:** Tumor volume, p-IRF3, p-STING. **Down-regulation:** CD45CD3, CD45CD3CD8CD69, CD45CD11cCD80, CD45CD3CD11cSINFEKL, IFN-γ, CD80, CD86, CXCL-10, IFN-β | 3.cGAS(-/-), STING(-/-)-RT-RECQL4 OE/H22 mice | **Unchanged indicator:** Primary tumors, Secondary tumors, CD45/CD3/CD8/CD69, CD45/CD3, CD45/CD3/CD11c/SINFEKL, IFN-γ | 4.RT-RECQL4 OE-Dnase-1/BMDCs | **Down-regulation:** p-STING, p-TBK1, p-IRF3 |  |  |
| #19 | Wang et al., 2022 | Sorafenib combined with STAT3 knockdown triggers ER stress-induced HCC apoptosis and cGAS-STING-mediated anti-tumor immunity | Liver Neoplasms | 1.sh-STAB-Sora/Huh7 cells | **Up-regulation:** ATF44, CHOP, BAX, C-Caspase3, Aggresomes, ROS. **Down-regulation:** Bcl-2 | 2.sh-STAT3-Sora/(Hepa1-6)mice | **Down-regulation:** Tumor volume, Tumor weight, Tim3CD8, Tim3NKcells, Tim3DC+. **Up-regulation:** CHOP, C-Caspase3, (IFN-γ, Perforin+, TNF-α+)CD8+, NK+cells, DC+, CD11c+DCs | 3.sh-STAT3-Sora/BMDCs | **Up-regulation:** (CD86, MHC-2, MHC-1)CD103+DC, MHC-2, CD8+DC, CD86CD11b+DC, IFNA1, IFNB1. **Down-regulation:** CD86+CD8+, CD11b+cDC2 | 4.H-151-sh-STAT3-Sora/BMDCs | **Down-regulation:** p-TBK1, p-IRF3 | 5.sh-STAT3+Sora+anti-IFNAR/(Hepa1-6)mice | **Up-regulation:** Tumor volume, Tumor weight, p-IRF3+CD103+DC, CD11B-CD103+. **Down-regulation:** Survival, CD8+T cells, NK cells, IFN-γ+CD8+, CD11b+CD103+DC, CD103+DC, |
| #20 | Ao et al., 2024 | STING agonist-based hydrogel enhances immune activation in synergy with radiofrequency ablation for hepatocellular carcinoma treatment | Liver Neoplasms | 1.iRFA/H22 mice | **Up-regulation:** F4/80+CD206+, Treg. **Unchanged indicators:** CD11b+F4/80+, CD11c+, F4/80+CD86+, CD11c+MHC-2+, CD3+CD8+. **Down-regulation:** CD8+IFN-γ+, CD8+GrzmB+, CD8+TNF-α+ | 2.ALG@MSA-2/(RAW264.7, BMDCs)H22 | **Up-regulation:** F4/80+CD86+, F4/80+CD206+, CD11c+MHC-2+ | 3.iRFA-ALG@MSA-2/H22 mice | **Up-regulation:** CD11B+CD86+, CD11c+MHC-2+, CD11c+CD86+, CD3+CD8+, NK1.1+, CD8+IFN-γ+, CD8+GrzmB+, CD8+TNF-α+. **Down-regulation:** CD11B+CD206+, Treg |  |  |  |  |
| #21 | Su et al., 2023 | TAK1 deficiency promotes liver injury and tumorigenesis via ferroptosis and macrophage cGAS-STING signalling | Liver Neoplasms | 1.TAK1 DHEP/mice | **Up-regulation:** ALT, AST, Sirius Red, α-SMA, Liver weight, F4/80, Arg-1, iNOS, CD206, CD86, CD206, CD163, TNF-α, IL-6, IL1β, IL-10, STING, cGAS, p-TBK1, p-STING, p-NF-ΚB, p-IRF3, IFN-β, Ferritin, 4-HNE, NRF2, NQO1, GSTP1, GCLC. **Down-regulation:** p-STAT1, Hepcidin, SOD | 2.C-176-TAK1 DHEP/mice | **Down-regulation:** AST, ALT, Sirius-Red, α-SMA, TGF-β1, Colla1, Timp1, Liver weight. **Up-regulation:** IFN-β, NF-Κb, IL-6, TNF-α, F4/80, CD206+ | 3.Fer-1-TAK1 HEP/mice | **Down-regulation:** STING, cGAS, p-TBK1, p-STING, p-NF-ΚB, p-IRF3, ALT, AST, α-SMA, Sirius Red, Ki67, Liver weight, 8-OHdG | 4.Anti-8-OHdG-TAK1 Hep/mice | **Down-regulation:** STING, cGAS, p-TBK1, p-STING, p-NF-ΚB, p-IRF3, ALT, AST, IFN-β, IL-6, TNF-α, iNOS |  |  |
| #22 | Thomsen et al., 2019 | The cGAS-STING pathway is a therapeutic target in a preclinical model of hepatocellular carcinoma | Liver Neoplasms | 1.3'3-cAIMP/Kupffer cells | Viperin, ISG15, STING | 2.Lipo-Clodronate-3'3'-cAIMP/DEN mice | **Down-regulation:** IFN-β, Viperin, ISG15, F4/80 | 3.3'3-cAIMP/mice | **Up-regulation:** p-STAT1, Viperin, LC3-2, C-Caspase3, Viperin, CXCL-10, MHC-1, STING. **Down-regulation:** MHC-2 | 4.IFN-α, TNF-α/Huh7 cells | **Up-regulation:** p-STAT1, p-p65, C-Caspase3 | 5.STING gt/DEN mice | **Up-regulation:** p-yH2AX, Ki-67, AFP, Tumor size, IL-6, Ifng. **Down-regulation:** C-Caspase3, Percent survival, p-STAT1, p-p65, C-Caspase3, p-STAT3, LC3-a, LC3-b. **Unchanged indicators:** ISG15, ISG56 |
|  |  |  |  | 6.3'3-cAIMP/DEN mice | **Down-regulation:** number and size of nodules, AFP, Tumours. **Up-regulation:** CD8, C-Caspase3, F4/80 |  |  |  |  |  |  |  |  |
| #23 | Huang et al., 2021 | Wnt/β-catenin inhibitor ICG-001 enhances the antitumor efficacy of radiotherapy by increasing radiation-induced DNA damage and improving tumor immune microenvironment in hepatocellular carcinoma | Liver Neoplasms | 1.IR-ICG-001/Hepa1-6 mice | **Down-regulation:** Tumor volume, CD8+/Treg. **Up-regulation:** Survival, CD8+CD3+, CD8+T cells, IFN-γ+CD8+. IFN-γ+CD4+, CD8+Treg, CD83+DC+, CD86+F4/80+CD11b+, γH2AX, p-STING, p-IRF3, p-TBK1, TIL+CD8+, T EMCD8+, CD8+CD3+, IFN-γ+CD4+ | 2.ICG-001/Hepa1-6， HCC-LM3 cells | **Down-regulation:** Survival fraction. **Up-regulation:** γ-H2AX | 3.C-176, Amlexanox-IR-ICG-001/Hepa1-6 mice | **Up-regulation:** Tumor volume. **Down-regulation:** TIL+CD8+, CD8+CD3+, INF-γ+CD8+ |  |  |  |  |
| #24 | Cen et al., 2021 | ZnS@BSA Nanoclusters Potentiate Efficacy of Cancer Immunotherapy | Liver Neoplasms | 1.ZnS@BSA(PH=6)/LM3, Hepa1-6 | **Down-regulation:** Cell viability, MMP. **Up-regulation:** Apoptosis rate, H2S, ROS, PE/FITC, mtDNA, IFN-β, CXCL-10, p-TBK1, p-STING | 2.ZnS@BSA/Hepa1-6 mice | Up-regulation: Tumor volume, Tumor weight, Survival rate, CD8+ T cells, Ki67, TUNEL | 3.C-176-ZnS@BSA/Hepa1-6 mice | **Up-regulation:** Ifnb1, ISG56, IFNβ, CXCL-10, p-TBK1, p-STING | 4.aPD-L1-ZnS@BSA/Hepa1-6 mice | **Down-regulation:** Tumor volume, Tumor weight, CD8+LAG-3+. **Up-regulation:** DC, CD8+T cells, CD8+CD39+ | 5.ZnS@BSA/(The scondary Hepa1-6 tumors)mice | **Down-regulation:** Tumor volume, Tumor weight. **Up-regulation:** p-STING, p-TBK1 |

**Supplementary Table 7.** Summary of key findings from studies investigating the role of cGAS-STING pathway in Parasitic liver disease.

| **Item** | **Study** | **Title** | **Disease** | Treatment/  Method | Key Findings | Treatment/  Method | Key Findings | Treatment/  Method | Key Findings | Treatment/  Method | Key Findings | Treatment/  Method | Key Findings |
| --- | --- | --- | --- | --- | --- | --- | --- | --- | --- | --- | --- | --- | --- |
| #1 | Liang et al., 2022 | cGAS exacerbates Schistosoma japonicum infection in a STING-type I IFN-dependent and independent manner | Parasitic liver disease | 1.7W Sj-cGAS KO/mice | **Down-regulation:** Liver damage, AST, ALT, Granuloma size, Collagen fiber, Collagen 1, Collagen 3, α-SMA, Ifnb1, IFN-β, p-TBK1 | 2.7W Sj-STING KO/mice | **Down-regulation:** p-TBK1, IFN-β, Ifnb, Liver damage, ALT, AST, Granuloma size. **Up-regulation:** Survival. **Unchanged indicators:** Collagen 1, α-SMA | 3.4W Sj/mice(Liver) | **Up-regulation:** Ifnb1, cGAMP. **Unchanged indicators:** cGAS, STING | 4.Sj-IFN-β/mice | **Up-regulation:** Liver damage, ALT, AST, Granuloma size | 5.Sj-Clodronate liposomes/mice | **Down-regulation:** Ifnb1, IFN-β |
|  |  |  |  | 6.Sj-Clodronat liposomes-cGAS KO/mice | **Down-regulation:** Ifnb1, CXCL-10 | 7.Sj adult DNA-cGAS KO/macrophages | **Down-regulation:** Ifnb1, CXCL-10, p-TBK1, p-IRF3 | 8.ISD-cGAS KO/macrophages | **Down-regulation:** p-TBK1, p-IRF3 |  |  |  |  |
| #2 | Liang et al., 2022 | cGAS exacerbates Schistosoma japonicum infection in a STING-type I IFN-dependent and independent manner | Parasitic liver disease | 1.DNA S.mansoni-cGAS(-/-) KO, STING(-/-) KO/mice | **Down-regulation:** IFN-β | 2.Carcariae-STING(-/-) KO/mice | **Down-regulation:** Worm burden recovery. **Unchanged indicators:** Number of eggs, Granuloma. **Up-regulation:** IL-17, TNF-α, IL-6 | 3.SWAP-STING(-/-) KO/mice | **Up-regulation:** IFN-γ. **Unchanged indicators:** IL-4, IL-10 | 4.100 Schistosomula-STING(-/-)/Neutrophils(mice) | **Down-regulation:** Viability of schistosomula | 5.IFN-g-100 Schistosomula-STING(-/-)/Neutrophils(mice) | **Down-regulation:** Viability of schistosomula. **Up-regulation:** Survival |

**Supplementary Table 8-1** PRISMA 2020 checklist.

| **Section and Topic** | **Item #** | **Checklist item** | **Location where item is reported** |
| --- | --- | --- | --- |
| **TITLE** | | |  |
| Title | 1 | Identify the report as a systematic review. | Page 1 |
| **ABSTRACT** | | |  |
| Abstract | 2 | See the PRISMA 2020 for Abstracts checklist. | Page 2 |
| **INTRODUCTION** | | |  |
| Rationale | 3 | Describe the rationale for the review in the context of existing knowledge. | Page 3-4 |
| Objectives | 4 | Provide an explicit statement of the objective(s) or question(s) the review addresses. | Page 3-4 |
| **METHODS** | | |  |
| Eligibility criteria | 5 | Specify the inclusion and exclusion criteria for the review and how studies were grouped for the syntheses. | Page 5 |
| Information sources | 6 | Specify all databases, registers, websites, organisations, reference lists and other sources searched or consulted to identify studies. Specify the date when each source was last searched or consulted. | Page 5 |
| Search strategy | 7 | Present the full search strategies for all databases, registers and websites, including any filters and limits used. | Supplementary Tables 2-1–2-7 |
| Selection process | 8 | Specify the methods used to decide whether a study met the inclusion criteria of the review, including how many reviewers screened each record and each report retrieved, whether they worked independently, and if applicable, details of automation tools used in the process. | Page 5 |
| Data collection process | 9 | Specify the methods used to collect data from reports, including how many reviewers collected data from each report, whether they worked independently, any processes for obtaining or confirming data from study investigators, and if applicable, details of automation tools used in the process. | Page 5-6 |
| Data items | 10a | List and define all outcomes for which data were sought. Specify whether all results that were compatible with each outcome domain in each study were sought (e.g. for all measures, time points, analyses), and if not, the methods used to decide which results to collect. | Supplementary Tables 6-1-6-7 |
|  | 10b | List and define all other variables for which data were sought (e.g. participant and intervention characteristics, funding sources). Describe any assumptions made about any missing or unclear information. | Supplementary Tables 6-1-6-7 |
| Study risk of bias assessment | 11 | Specify the methods used to assess risk of bias in the included studies, including details of the tool(s) used, how many reviewers assessed each study and whether they worked independently, and if applicable, details of automation tools used in the process. | Page 5-6 |
| Effect measures | 12 | Specify for each outcome the effect measure(s) (e.g. risk ratio, mean difference) used in the synthesis or presentation of results. | - |
| Synthesis methods | 13a | Describe the processes used to decide which studies were eligible for each synthesis (e.g. tabulating the study intervention characteristics and comparing against the planned groups for each synthesis (item #5)). | - |
|  | 13b | Describe any methods required to prepare the data for presentation or synthesis, such as handling of missing summary statistics, or data conversions. | - |
|  | 13c | Describe any methods used to tabulate or visually display results of individual studies and syntheses. | - |
|  | 13d | Describe any methods used to synthesize results and provide a rationale for the choice(s). If meta-analysis was performed, describe the model(s), method(s) to identify the presence and extent of statistical heterogeneity, and software package(s) used. | - |
|  | 13e | Describe any methods used to explore possible causes of heterogeneity among study results (e.g. subgroup analysis, meta-regression). | - |
|  | 13f | Describe any sensitivity analyses conducted to assess robustness of the synthesized results. | - |
| Reporting bias assessment | 14 | Describe any methods used to assess risk of bias due to missing results in a synthesis (arising from reporting biases). | Page 5-6 |
| Certainty assessment | 15 | Describe any methods used to assess certainty (or confidence) in the body of evidence for an outcome. | - |
| **RESULTS** | | |  |
| Study selection | 16a | Describe the results of the search and selection process, from the number of records identified in the search to the number of studies included in the review, ideally using a flow diagram. | Fig.1 |
|  | 16b | Cite studies that might appear to meet the inclusion criteria, but which were excluded, and explain why they were excluded. | Supplementary Tables 4-1-4-7 |
| Study characteristics | 17 | Cite each included study and present its characteristics. | Table 1-4 |
| Risk of bias in studies | 18 | Present assessments of risk of bias for each included study. | Supplementary Fig 1-2 |
| Results of individual studies | 19 | For all outcomes, present, for each study: (a) summary statistics for each group (where appropriate) and (b) an effect estimate and its precision (e.g. confidence/credible interval), ideally using structured tables or plots. | Supplementary Tables 6-1-6-7 |
| Results of syntheses | 20a | For each synthesis, briefly summarise the characteristics and risk of bias among contributing studies. | Table S7 |
|  | 20b | Present results of all statistical syntheses conducted. If meta-analysis was done, present for each the summary estimate and its precision (e.g. confidence/credible interval) and measures of statistical heterogeneity. If comparing groups, describe the direction of the effect. | - |
|  | 20c | Present results of all investigations of possible causes of heterogeneity among study results. | - |
|  | 20d | Present results of all sensitivity analyses conducted to assess the robustness of the synthesized results. | - |
| Reporting biases | 21 | Present assessments of risk of bias due to missing results (arising from reporting biases) for each synthesis assessed. | Not applicable |
| Certainty of evidence | 22 | Present assessments of certainty (or confidence) in the body of evidence for each outcome assessed. | - |
| **DISCUSSION** | | |  |
| Discussion | 23a | Provide a general interpretation of the results in the context of other evidence. | Page 36-38 |
|  | 23b | Discuss any limitations of the evidence included in the review. | Page 36-38 |
|  | 23c | Discuss any limitations of the review processes used. | Page 36-38 |
|  | 23d | Discuss implications of the results for practice, policy, and future research. | Page 36-38 |
| **OTHER INFORMATION** | | |  |
| Registration and protocol | 24a | Provide registration information for the review, including register name and registration number, or state that the review was not registered. | Page 5 |
|  | 24b | Indicate where the review protocol can be accessed, or state that a protocol was not prepared. | Page 5 |
|  | 24c | Describe and explain any amendments to information provided at registration or in the protocol. | Page 5 |
| Support | 25 | Describe sources of financial or non-financial support for the review, and the role of the funders or sponsors in the review. | Page 21 |
| Competing interests | 26 | Declare any competing interests of review authors. | Page 21 |
| Availability of data, code and other materials | 27 | Report which of the following are publicly available and where they can be found: template data collection forms; data extracted from included studies; data used for all analyses; analytic code; any other materials used in the review. | Supplementary Tables 6-1-6-7 |

**Supplementary Table 8-2** RISMA 2020 for Abstracts checklist.

| **Section and Topic** | **Item #** | **Checklist item** | **Reported (Yes/No)** |
| --- | --- | --- | --- |
| **TITLE** | | |  |
| Title | 1 | Identify the report as a systematic review. | Page 1 |
| **BACKGROUND** | | |  |
| Objectives | 2 | Provide an explicit statement of the main objective(s) or question(s) the review addresses. | Page 2 |
| **METHODS** | | |  |
| Eligibility criteria | 3 | Specify the inclusion and exclusion criteria for the review. |  |
| Information sources | 4 | Specify the information sources (e.g. databases, registers) used to identify studies and the date when each was last searched. | Page 2 |
| Risk of bias | 5 | Specify the methods used to assess risk of bias in the included studies. | Page 5 |
| Synthesis of results | 6 | Specify the methods used to present and synthesise results. | Page 5 |
| **RESULTS** | | |  |
| Included studies | 7 | Give the total number of included studies and participants and summarise relevant characteristics of studies. | Page 6 |
| Synthesis of results | 8 | Present results for main outcomes, preferably indicating the number of included studies and participants for each. If meta-analysis was done, report the summary estimate and confidence/credible interval. If comparing groups, indicate the direction of the effect (i.e. which group is favoured). | Page 6 |
| **DISCUSSION** | | |  |
| Limitations of evidence | 9 | Provide a brief summary of the limitations of the evidence included in the review (e.g. study risk of bias, inconsistency and imprecision). | Page 37-38 |
| Interpretation | 10 | Provide a general interpretation of the results and important implications. | Page 37-38 |
| **OTHER** | | |  |
| Funding | 11 | Specify the primary source of funding for the review. | Page 41 |
| Registration | 12 | Provide the register name and registration number. | Page 5 |

| **Supplementary Table 9-1.** Search strategies for Hepatitis and STING in Medical Subject Headings. | | | |
| --- | --- | --- | --- |
| Item | Databases | Advanced Search | Results |
| #1 | Pubmed | (cGAS-STING OR STING) | 33,607 |
| #2 | Pubmed | (Hepatitis OR Hepatitis, Alcoholic OR Hepatitis, Chronic OR Hepatitis B, Chronic OR Hepatitis C, Chronic OR Hepatitis D, Chronic OR Hepatitis, Autoimmune OR Hepatitis, Viral, Human OR Hepatitis A OR Hepatitis B OR Hepatitis C OR Hepatitis D OR Hepatitis E OR Hepatitis Virus OR Virus, Hepatitis OR Viruses, Hepatitis) | 251,317 |
| #3 | Pubmed | (#1 and #2) | 76 |
| #4 | Scopus | TITLE-ABS-KEY (cgas-sting) OR TITLE-ABS-KEY (sting) | 27,151 |
| #5 | Scopus | TITLE-ABS-KEY(Hepatitis) OR TITLE-ABS-KEY(Hepatitis, Alcoholic) OR TITLE-ABS-KEY(Hepatitis, Chronic) OR TITLE-ABS-KEY(Hepatitis B, Chronic) OR TITLE-ABS-KEY(Hepatitis C, Chronic) OR TITLE-ABS-KEY(Hepatitis D, Chronic) OR TITLE-ABS-KEY (Hepatitis, Autoimmune) OR TITLE-ABS-KEY(Hepatitis, Viral, Human) OR TITLE-ABS-KEY (Hepatitis A) OR TITLE-ABS-KEY(Hepatitis B) OR TITLE-ABS-KEY(Hepatitis C) OR TITLE-ABS-KEY(Hepatitis D) OR TITLE-ABS-KEY(Hepatitis E) OR TITLE-ABS-KEY(Hepatitis Virus) OR TITLE-ABS-KEY (Virus, Hepatitis) OR TITLE-ABS-KEY(Viruses, Hepatitis) | 2,752 |
| #6 | Scopus | (#4 and #5) | 302 |
| #7 | Embase | 'cgas sting' OR sting | 15,593 |
| #8 | Embase | Hepatitis OR Hepatitis, Alcoholic OR Hepatitis, Chronic OR Hepatitis B, Chronic OR Hepatitis C, Chronic OR Hepatitis D, Chronic OR Hepatitis, Autoimmune OR Hepatitis, Viral, Human OR Hepatitis A OR Hepatitis B OR Hepatitis C OR Hepatitis D OR Hepatitis E OR Hepatitis Virus OR Virus, Hepatitis OR Viruses, Hepatitis | 298,640 |
| #9 | Embase | #8 and #9 | 170 |
| #10 | Web of Science | TS=(cGAS-STING OR STING) | 33,874 |
| #11 | Web of Science | TS=(Hepatitis OR Hepatitis, Alcoholic OR Hepatitis, Chronic OR Hepatitis B, Chronic OR Hepatitis C, Chronic OR Hepatitis D, Chronic OR Hepatitis, Autoimmune OR Hepatitis, Viral, Human OR Hepatitis A OR Hepatitis B OR Hepatitis C OR Hepatitis D OR Hepatitis E OR Hepatitis Virus OR Virus, Hepatitis OR Viruses, Hepatitis) | 454,112 |
| #12 | Web of Science | #10 and #11 | 268 |

| **Supplementary Table 9-2.** Search strategies for Liver Neoplasms and STING in Medical Subject Headings. | | | |
| --- | --- | --- | --- |
| Item | Databases | Advanced Search | Results |
| #1 | Pubmed | (cGAS-STING OR STING) | 33,607 |
| #2 | Pubmed | (Neoplasms, Hepatic OR Neoplasms, Liver OR Liver Neoplasm OR Neoplasm, Liver OR Hepatic Neoplasms OR Hepatic Neoplasm OR Neoplasm, Hepatic OR Cancer of Liver OR Hepatocellular Cancer OR Cancers, Hepatocellular OR Hepatocellular Cancers OR Hepatic Cancer OR Cancer, Hepatic OR Cancers, Hepatic OR Hepatic Cancers OR Liver Cancer OR Cancer, Liver OR Cancers, Liver OR Liver Cancers OR Cancer of the Liver OR Cancer, Hepatocellular) | 41,309 |
| #3 | Pubmed | (#1 and #2) | 30 |
| #4 | Scopus | TITLE-ABS-KEY (cgas-sting) OR TITLE-ABS-KEY (sting) | 27,151 |
| #5 | Scopus | TITLE-ABS-KEY(Neoplasms, Hepatic) OR TITLE-ABS-KEY(Neoplasms, Liver) OR TITLE-ABS-KEY(Liver Neoplasm) OR TITLE-ABS-KEY(Neoplasm, Liver) OR TITLE-ABS-KEY(Hepatic Neoplasms) OR TITLE-ABS-KEY(Hepatic Neoplasm) OR TITLE-ABS-KEY(Neoplasm, Hepatic) OR TITLE-ABS-KEY(Cancer of Liver) OR TITLE-ABS-KEY(Hepatocellular Cancer) OR TITLE-ABS-KEY(Cancers, Hepatocellular) OR TITLE-ABS-KEY(Hepatocellular Cancers) OR TITLE-ABS-KEY(Hepatic Cancer) OR TITLE-ABS-KEY(Cancer, Hepatic) OR TITLE-ABS-KEY(Cancers, Hepatic) OR TITLE-ABS-KEY(Hepatic Cancers) OR TITLE-ABS-KEY(Liver Cancer) OR TITLE-ABS-KEY(Cancer, Liver) OR TITLE-ABS-KEY(Cancers, Liver) OR TITLE-ABS-KEY(Liver Cancers) OR TITLE-ABS-KEY(Cancer of the Liver) OR TITLE-ABS-KEY(Cancer, Hepatocellular) | 446,298 |
| #6 | Scopus | (#4 and #5) | 201 |
| #7 | Embase | 'cgas sting' OR sting | 15,593 |
| #8 | Embase | (Neoplasms, Hepatic OR Neoplasms, Liver OR Liver Neoplasm OR Neoplasm, Liver OR Hepatic Neoplasms OR Hepatic Neoplasm OR Neoplasm, Hepatic OR Cancer of Liver OR Hepatocellular Cancer OR Cancers, Hepatocellular OR Hepatocellular Cancers OR Hepatic Cancer OR Cancer, Hepatic OR Cancers, Hepatic OR Hepatic Cancers OR Liver Cancer OR Cancer, Liver OR Cancers, Liver OR Liver Cancers OR Cancer of the Liver OR Cancer, Hepatocellular) | 132,262 |
| #9 | Embase | #8 and #9 | 74 |
| #10 | Web of Science | TS=(cGAS-STING OR STING) | 33,874 |
| #11 | Web of Science | TS=(Neoplasms, Hepatic OR Neoplasms, Liver OR Liver Neoplasm OR Neoplasm, Liver OR Hepatic Neoplasms OR Hepatic Neoplasm OR Neoplasm, Hepatic OR Cancer of Liver OR Hepatocellular Cancer OR Cancers, Hepatocellular OR Hepatocellular Cancers OR Hepatic Cancer OR Cancer, Hepatic OR Cancers, Hepatic OR Hepatic Cancers OR Liver Cancer OR Cancer, Liver OR Cancers, Liver OR Liver Cancers OR Cancer of the Liver OR Cancer, Hepatocellular) | 586,051 |
| #12 | Web of Science | #10 and #11 | 281 |

| **Supplementary Table 9-3.** Search strategies for NAFLD/ALD and STING in Medical Subject Headings. | | | |
| --- | --- | --- | --- |
| Item | Databases | Advanced Search | Results |
| #1 | Pubmed | (cGAS-STING OR STING) | 33,607 |
| #2 | Pubmed | (Non alcoholic Fatty Liver Disease OR NAFLD OR Nonalcoholic Fatty Liver Disease OR Fatty Liver, Nonalcoholic OR Fatty Livers, Nonalcoholic OR Liver, Nonalcoholic Fatty OR Livers, Nonalcoholic Fatty OR Nonalcoholic Fatty Liver OR Nonalcoholic Fatty Livers OR Nonalcoholic Steatohepatitis OR Nonalcoholic Steatohepatitides OR Steatohepatitides, Nonalcoholic OR Steatohepatitis, Nonalcoholic OR Alcoholic Liver Diseases OR Alcoholic Liver Disease OR Liver Disease, Alcoholic) | 48,933 |
| #3 | Pubmed | (#1 and #2) | 41 |
| #4 | Scopus | TITLE-ABS-KEY (cgas-sting) OR TITLE-ABS-KEY (sting) | 27,151 |
| #5 | Scopus | TITLE-ABS-KEY(Non alcoholic Fatty Liver Disease) OR TITLE-ABS-KEY(NAFLD) OR TITLE-ABS-KEY(Nonalcoholic Fatty Liver Disease) OR TITLE-ABS-KEY(Fatty Liver, Nonalcoholic) OR TITLE-ABS-KEY(Fatty Livers, Nonalcoholic) OR TITLE-ABS-KEY(Liver, Nonalcoholic Fatty) OR TITLE-ABS-KEY(Livers, Nonalcoholic Fatty) OR TITLE-ABS-KEY(Nonalcoholic Fatty Liver) OR TITLE-ABS-KEY(Nonalcoholic Fatty Livers) OR TITLE-ABS-KEY(Nonalcoholic Steatohepatitis) OR TITLE-ABS-KEY(Nonalcoholic Steatohepatitides) OR TITLE-ABS-KEY(Steatohepatitides, Nonalcoholic) OR TITLE-ABS-KEY(Steatohepatitis, Nonalcoholic) OR TITLE-ABS-KEY(Alcoholic Liver Diseases) OR TITLE-ABS-KEY(Alcoholic Liver Disease) OR TITLE-ABS-KEY(Liver Disease, Alcoholic) | 82,835 |
| #6 | Scopus | (#4 and #5) | 68 |
| #7 | Embase | 'cgas sting' OR sting | 15,593 |
| #8 | Embase | TS=(Non alcoholic Fatty Liver Disease OR NAFLD OR Nonalcoholic Fatty Liver Disease OR Fatty Liver, Nonalcoholic OR Fatty Livers, Nonalcoholic OR Liver, Nonalcoholic Fatty OR Livers, Nonalcoholic Fatty OR Nonalcoholic Fatty Liver OR Nonalcoholic Fatty Livers OR Nonalcoholic Steatohepatitis OR Nonalcoholic Steatohepatitides OR Steatohepatitides, Nonalcoholic OR Steatohepatitis, Nonalcoholic OR Alcoholic Liver Diseases OR Alcoholic Liver Disease OR Liver Disease, Alcoholic) | 134.519 |
| #9 | Embase | #8 and #9 | 91 |
| #10 | Web of Science | TS=(cGAS-STING OR STING) | 33,874 |
| #11 | Web of Science | TS=(Non alcoholic Fatty Liver Disease OR NAFLD OR Nonalcoholic Fatty Liver Disease OR Fatty Liver, Nonalcoholic OR Fatty Livers, Nonalcoholic OR Liver, Nonalcoholic Fatty OR Livers, Nonalcoholic Fatty OR Nonalcoholic Fatty Liver OR Nonalcoholic Fatty Livers OR Nonalcoholic Steatohepatitis OR Nonalcoholic Steatohepatitides OR Steatohepatitides, Nonalcoholic OR Steatohepatitis, Nonalcoholic OR Alcoholic Liver Diseases OR Alcoholic Liver Disease OR Liver Disease, Alcoholic) | 111,574 |
| #12 | Web of Science | #10 and #11 | 93 |

| **Supplementary Table 9-4.** Search strategies for Liver Cirrhosis and STING in Medical Subject Headings. | | | |
| --- | --- | --- | --- |
| Item | Databases | Advanced Search | Results |
| #1 | Pubmed | (cGAS-STING OR STING) | 33,607 |
| #2 | Pubmed | (Hepatic Cirrhosis OR Cirrhosis, Hepatic OR Cirrhosis, Liver OR Fibrosis, Liver OR Liver Fibrosis) | 28,874 |
| #3 | Pubmed | (#1 and #2) | 23 |
| #4 | Scopus | TITLE-ABS-KEY (cgas-sting) OR TITLE-ABS-KEY (sting) | 27,151 |
| #5 | Scopus | TITLE-ABS-KEY ( hepatic AND cirrhosis ) OR TITLE-ABS-KEY ( cirrhosis, AND hepatic ) OR TITLE-ABS-KEY ( cirrhosis, AND liver ) OR TITLE-ABS-KEY ( fibrosis, AND liver ) OR TITLE-ABS-KEY ( liver AND fibrosis ) ) AND ( TITLE-ABS-KEY ( cgas-sting ) OR TITLE-ABS-KEY ( sting ) | 2,752 |
| #6 | Scopus | (#4 and #5) | 89 |
| #7 | Embase | 'cgas sting' OR sting | 15,593 |
| #8 | Embase | (Hepatic Cirrhosis OR Cirrhosis, Hepatic OR Cirrhosis, Liver OR Fibrosis, Liver OR Liver Fibrosis) | 331,229 |
| #9 | Embase | #8 and #9 | 105 |
| #10 | Web of Science | TS=(cGAS-STING OR STING) | 33,874 |
| #11 | Web of Science | TS=(Hepatic Cirrhosis OR Cirrhosis, Hepatic OR Cirrhosis, Liver OR Fibrosis, Liver OR Liver Fibrosis) | 20,745 |
| #12 | Web of Science | #10 and #11 | 101 |

| **Supplementary Table 9-5.** Search strategies for hepatic Reperfusion Injury and STING in Medical Subject Headings. | | | |
| --- | --- | --- | --- |
| Item | Databases | Advanced Search | Results |
| #1 | Pubmed | (cGAS-STING OR STING) | 33,607 |
| #2 | Pubmed | (hepatic Reperfusion Injuries OR hepatic Reperfusion Damage OR hepatic Damage, Reperfusion OR hepatic Reperfusion Damages OR hepatic Ischemia-Reperfusion Injury OR hepatic Ischemia Reperfusion Injury OR hepatic Injury, Ischemia-Reperfusion OR hepatic Injury, Ischemia Reperfusion OR hepatic Ischemia-Reperfusion Injuries OR hepatic Injury, Reperfusion) | 1645 |
| #3 | Pubmed | (#1 and #2) | 6 |
| #4 | Scopus | TITLE-ABS-KEY (cgas-sting) OR TITLE-ABS-KEY (sting) | 27,151 |
| #5 | Scopus | TITLE-ABS-KEY(hepatic Reperfusion Injuries) OR TITLE-ABS-KEY-AUTH(hepatic Reperfusion Damage) OR TITLE-ABS-KEY(hepatic Damage, Reperfusion) OR TITLE-ABS-KEY(hepatic Reperfusion Damages) OR TITLE-ABS-KEY(hepatic Ischemia-Reperfusion Injury) OR TITLE-ABS-KEY(hepatic Ischemia Reperfusion Injury) OR TITLE-ABS-KEY(hepatic Injury, Ischemia-Reperfusion) OR TITLE-ABS-KEY(hepatic Injury, Ischemia Reperfusion) OR TITLE-ABS-KEY(hepatic Ischemia-Reperfusion Injuries) OR TITLE-ABS-KEY(hepatic Injury, Reperfusion) | 6735 |
| #6 | Scopus | (#4 and #5) | 302 |
| #7 | Embase | 'cgas sting' OR sting | 15,593 |
| #8 | Embase | (hepatic Reperfusion Injuries OR hepatic Reperfusion Damage OR hepatic Damage, Reperfusion OR hepatic Reperfusion Damages OR hepatic Ischemia-Reperfusion Injury OR hepatic Ischemia Reperfusion Injury OR hepatic Injury, Ischemia-Reperfusion OR hepatic Injury, Ischemia Reperfusion OR hepatic Ischemia-Reperfusion Injuries OR hepatic Injury, Reperfusion) | 8308 |
| #9 | Embase | #8 and #9 | 17 |
| #10 | Web of Science | TS=(cGAS-STING OR STING) | 33,874 |
| #11 | Web of Science | TS=(hepatic Reperfusion Injuries OR hepatic Reperfusion Damage OR hepatic Damage, Reperfusion OR hepatic Reperfusion Damages OR hepatic Ischemia-Reperfusion Injury OR hepatic Ischemia Reperfusion Injury OR hepatic Injury, Ischemia-Reperfusion OR hepatic Injury, Ischemia Reperfusion OR hepatic Ischemia-Reperfusion Injuries OR hepatic Injury, Reperfusion) | 9912 |
| #12 | Web of Science | #10 and #11 | 15 |

| **Supplementary Table 9-6.** Search strategies for Chemical and Drug Induced Liver Injury and STING in Medical Subject Headings. | | | |
| --- | --- | --- | --- |
| Item | Databases | Advanced Search | Results |
| #1 | Pubmed | (cGAS-STING OR STING) | 33,607 |
| #2 | Pubmed | (Chemically-Induced Liver Toxicity OR Chemically Induced Liver Toxicity OR Chemically-Induced Liver Toxicities OR Liver Toxicities, Chemically-Induced OR Liver Toxicity, Chemically-Induced OR Toxicities, Chemically-Induced Liver OR Toxicity, Chemically-Induced Liver OR Drug-Induced Acute Liver Injury OR Drug Induced Acute Liver Injury OR Liver Injury, Drug-Induced, Acute OR Acute Liver Injury, Drug-Induced OR Acute Liver Injury, Drug Induced OR Hepatitis, Toxic OR Toxic Hepatitis OR Hepatitides, Toxic OR Toxic Hepatitides OR Drug-Induced Liver Disease OR Disease, Drug-Induced Liver OR Diseases, Drug-Induced Liver OR Drug Induced Liver Disease OR Drug-Induced Liver Diseases OR Liver Disease, Drug-Induced OR Liver Diseases, Drug-Induced OR Drug-Induced Liver Injury OR Drug Induced Liver Injury OR Drug-Induced Liver Injuries OR Injuries, Drug-Induced Liver OR Injury, Drug-Induced Liver OR Liver Injuries, Drug-Induced OR Liver Injury, Drug-Induced OR Liver Injury, Drug Induced OR Hepatitis, Drug-Induced OR Drug-Induced Hepatitides OR Drug-Induced Hepatitis OR Hepatitides, Drug-Induced OR Hepatitis, Drug Induced) | 12,143 |
| #3 | Pubmed | (#1 and #2) | 4 |
| #4 | Scopus | TITLE-ABS-KEY (cgas-sting) OR TITLE-ABS-KEY (sting) | 27,151 |
| #5 | Scopus | TITLE-ABS-KEY(Chemically-Induced Liver Toxicity) OR TITLE-ABS-KEY(Chemically Induced Liver Toxicity) OR TITLE-ABS-KEY(Chemically-Induced Liver Toxicities) OR TITLE-ABS-KEY(Liver Toxicities, Chemically-Induced) OR TITLE-ABS-KEY(Liver Toxicity, Chemically-Induced) OR TITLE-ABS-KEY(Toxicities, Chemically-Induced Liver) OR TITLE-ABS-KEY(Toxicity, Chemically-Induced Liver) OR TITLE-ABS-KEY(Drug-Induced Acute Liver Injury) OR TITLE-ABS-KEY(Drug Induced Acute Liver Injury) OR TITLE-ABS-KEY(Liver Injury, Drug-Induced, Acute) OR TITLE-ABS-KEY(Acute Liver Injury, Drug-Induced) OR TITLE-ABS-KEY(Acute Liver Injury, Drug Induced) OR TITLE-ABS-KEY(Hepatitis, Toxic) OR TITLE-ABS-KEY(Toxic Hepatitis) OR TITLE-ABS-KEY(Hepatitides, Toxic) OR TITLE-ABS-KEY(Toxic Hepatitides) OR TITLE-ABS-KEY(Drug-Induced Liver Disease) OR TITLE-ABS-KEY(Disease, Drug-Induced Liver) OR TITLE-ABS-KEY(Diseases, Drug-Induced Liver) OR TITLE-ABS-KEY(Drug Induced Liver Disease) OR TITLE-ABS-KEY(Drug-Induced Liver Diseases) OR TITLE-ABS-KEY(Liver Disease, Drug-Induced) OR TITLE-ABS-KEY(Liver Diseases, Drug-Induced) OR TITLE-ABS-KEY(Drug-Induced Liver Injury) OR TITLE-ABS-KEY(Drug Induced Liver Injury) OR TITLE-ABS-KEY(Drug-Induced Liver Injuries) OR TITLE-ABS-KEY(Injuries, Drug-Induced Liver) OR TITLE-ABS-KEY(Injury, Drug-Induced Liver) OR TITLE-ABS-KEY(Liver Injuries, Drug-Induced) OR TITLE-ABS-KEY(Liver Injury, Drug-Induced) OR TITLE-ABS-KEY(Liver Injury, Drug Induced) OR TITLE-ABS-KEY(Hepatitis, Drug-Induced) OR TITLE-ABS-KEY(Drug-Induced Hepatitides) OR TITLE-ABS-KEY(Drug-Induced Hepatitis) OR TITLE-ABS-KEY(Hepatitides, Drug-Induced) OR TITLE-ABS-KEY(Hepatitis, Drug Induced) | 118,259 |
| #6 | Scopus | (#4 and #5) | 161 |
| #7 | Embase | 'cgas sting' OR sting | 15,593 |
| #8 | Embase | (Chemically-Induced Liver Toxicity OR Chemically Induced Liver Toxicity OR Chemically-Induced Liver Toxicities OR Liver Toxicities, Chemically-Induced OR Liver Toxicity, Chemically-Induced OR Toxicities, Chemically-Induced Liver OR Toxicity, Chemically-Induced Liver OR Drug-Induced Acute Liver Injury OR Drug Induced Acute Liver Injury OR Liver Injury, Drug-Induced, Acute OR Acute Liver Injury, Drug-Induced OR Acute Liver Injury, Drug Induced OR Hepatitis, Toxic OR Toxic Hepatitis OR Hepatitides, Toxic OR Toxic Hepatitides OR Drug-Induced Liver Disease OR Disease, Drug-Induced Liver OR Diseases, Drug-Induced Liver OR Drug Induced Liver Disease OR Drug-Induced Liver Diseases OR Liver Disease, Drug-Induced OR Liver Diseases, Drug-Induced OR Drug-Induced Liver Injury OR Drug Induced Liver Injury OR Drug-Induced Liver Injuries OR Injuries, Drug-Induced Liver OR Injury, Drug-Induced Liver OR Liver Injuries, Drug-Induced OR Liver Injury, Drug-Induced OR Liver Injury, Drug Induced OR Hepatitis, Drug-Induced OR Drug-Induced Hepatitides OR Drug-Induced Hepatitis OR Hepatitides, Drug-Induced OR Hepatitis, Drug Induced) | 40,961 |
| #9 | Embase | #8 and #9 | 76 |
| #10 | Web of Science | TS=(cGAS-STING OR STING) | 33,874 |
| #11 | Web of Science | TS=(Chemically-Induced Liver Toxicity OR Chemically Induced Liver Toxicity OR Chemically-Induced Liver Toxicities OR Liver Toxicities, Chemically-Induced OR Liver Toxicity, Chemically-Induced OR Toxicities, Chemically-Induced Liver OR Toxicity, Chemically-Induced Liver OR Drug-Induced Acute Liver Injury OR Drug Induced Acute Liver Injury OR Liver Injury, Drug-Induced, Acute OR Acute Liver Injury, Drug-Induced OR Acute Liver Injury, Drug Induced OR Hepatitis, Toxic OR Toxic Hepatitis OR Hepatitides, Toxic OR Toxic Hepatitides OR Drug-Induced Liver Disease OR Disease, Drug-Induced Liver OR Diseases, Drug-Induced Liver OR Drug Induced Liver Disease OR Drug-Induced Liver Diseases OR Liver Disease, Drug-Induced OR Liver Diseases, Drug-Induced OR Drug-Induced Liver Injury OR Drug Induced Liver Injury OR Drug-Induced Liver Injuries OR Injuries, Drug-Induced Liver OR Injury, Drug-Induced Liver OR Liver Injuries, Drug-Induced OR Liver Injury, Drug-Induced OR Liver Injury, Drug Induced OR Hepatitis, Drug-Induced OR Drug-Induced Hepatitides OR Drug-Induced Hepatitis OR Hepatitides, Drug-Induced OR Hepatitis, Drug Induced) | 189,865 |
| #12 | Web of Science | #10 and #11 | 159 |

| **Supplementary Table 9-7.** Search strategies for Parasitic Liver Disease and STING in Medical Subject Headings. | | | |
| --- | --- | --- | --- |
| Item | Databases | Advanced Search | Results |
| #1 | Pubmed | (cGAS-STING OR STING) | 33,607 |
| #2 | Pubmed | (Parasitic Liver Diseases OR Parasitic Liver OR Diseases, Parasitic Liver OR Liver, Parasitic OR Parasitic Liver Disease) | 160 |
| #3 | Pubmed | (#1 and #2) | 0 |
| #4 | Scopus | TITLE-ABS-KEY (cgas-sting) OR TITLE-ABS-KEY (sting) | 27,151 |
| #5 | Scopus | TITLE-ABS-KEY(Parasitic Liver Diseases) OR TITLE-ABS-KEY(Parasitic Liver) OR TITLE-ABS-KEY(Diseases, Parasitic Liver) OR TITLE-ABS-KEY(Liver, Parasitic) OR TITLE-ABS-KEY(Parasitic Liver Disease) | 8718 |
| #6 | Scopus | (#4 and #5) | 7 |
| #7 | Embase | 'cgas sting' OR sting | 15,593 |
| #8 | Embase | (Parasitic Liver Diseases OR Parasitic Liver OR Diseases, Parasitic Liver OR Liver, Parasitic OR Parasitic Liver Disease) | 6128 |
| #9 | Embase | #8 and #9 | 3 |
| #10 | Web of Science | TS=(cGAS-STING OR STING) | 33,874 |
| #11 | Web of Science | TS=(Parasitic Liver Diseases OR Parasitic Liver OR Diseases, Parasitic Liver OR Liver, Parasitic OR Parasitic Liver Disease) | 40,206 |
| #12 | Web of Science | #10 and #11 | 42 |

| **Supplementary Table 10.** Eligibility criteria. | | |
| --- | --- | --- |
| Criteria | Inclusion | Exclusion |
| Population | • Mice models and cell models: NAFLD/ALD model, viral hepatitis model, particularly those involving hepatitis B and C virus infections, liver Neoplasms model, HIRI model, liver injury model, models of parasitic liver disease, liver Cirrhosis model | • Research unrelated to liver diseases • Animal and cellular studies have not been performed for Sting pathway-related testing or analysis |
| Intervention | • Knockdown or overexpression of sting or its related proteins • STING agonists or antagonists were used • RNA interference (RNAi) was used to silence the expression of STING or its related proteins | • There were no interventions around STING or STING-related impact factors • There are no STING-related interventions around liver disease • Interventions with unclear or unchanged outcome measures • Interventions that were not outcome measures of the primary observation |
| Comparison | • Vehicle control: Empty vector was used to transfuse cells or injection animals • Solvent control: Treatment was performed using a solvent that dissolved the drug or compound • Gene knockout or knockdown control: In gene editing experiments, the control group may be wild-type or cells/animals treated with gene editing vectors only • Normal control: In pathological model studies, normal control refers to normal cells or animals without induced disease models | • Studies lacking an appropriate control group: for example, no control group was set up, or the control group was not designed properly • There were significant differences between the control and experimental groups, resulting in non-comparable results |
| Outcomes | • Liver function indexes were alanine aminotransferase (ALT) and aspartate aminotransferase (AST) • Inflammatory response indicators: p-IRF3, IFN-β, p-p65/p65, IL6, IL1b, TNF-α, p-p62, IL10, F4/80 • Apoptosis markers: Bax/Bcl2, C-Casp3, clv-PARP/PARP • Cell signaling pathway indicators: p-AKT/t-AKT, p-GSK3β, TBK1 • Glucose metabolism indicators: GCK, PFK, PK, G-6-pase, PEPCK, PC • Fibrosis markers: α-SMA, Col1a1, Col3a1, TGF-β, GFAP, Fn • The degree of liver fibrosis, changes in liver fat content and so on  • lipid droplets: ROS, SOD, CAT, and caspase-3 • tumor cell proliferation, Tregs, MDSCs, CD8+ T cell count, activation status, cytotoxic function (e.g., expression of Granzyme B and Perforin) | • Unclear reporting of results: The description of study results is not clear enough to accurately understand the measurement methods and results of outcome measures • Incomplete reporting of results: Incomplete reporting of study results, such as missing key data, statistical analysis results, etc • Qualitative but not quantitative results: For outcome indicators that required statistical analysis, studies that provided only qualitative descriptions without quantitative data were excluded |
| Type of design | • In vitro cell experiments • Animal studies (eg, mouse, rat models) • Genetic manipulation studies (eg, gene knockouts, overexpression) • Pharmacological studies (eg, using STING pathway agonists or inhibitors) • Mechanistic studies (eg, exploring the molecular mechanisms of the STING pathway in liver diseases) | • Meta-analyses, case reports, and review articles • Non-experimental studies, such as observational studies and epidemiological studies • Studies using only computer simulation or bioinformatics methods • Meeting abstracts with full methodological details or outcome data were not provided |
| Years of publication | • From inception to April 15, 2024 |  |
| Publication type | • Published and unpublished articles | • Abstract only  • Book chapter review • Letters • Editorials • Systematic review/Review |
| Language | • English | • Non-English |

| **Supplementary Table 11-1.** List of excluded studies in NFLD/ALD. | | | | |
| --- | --- | --- | --- | --- |
| Item | Year | Title | DOI | Reason for exclusion |
| #1 | 2014 | A cross-cultural analysis of Jammu, Kashmir and Ladakh (India) medicinal plant use | 10.1016/j.jep.2014.06.029 | Not focused on STING pathway mechanisms |
| #2 | 2020 | A Potential Role for Mitochondrial DNA in the Activation of Oxidative Stress and Inflammation in Liver Disease | 10.1155/2020/5835910 | Not focused on STING pathway mechanisms |
| #3 | 2022 | Activation of cGAS/STING signaling pathway and its immunological role in the progression of nonalcoholic fatty liver disease | 10.3760/cma.j.cn501113-20211011-00503 | Non-experimental or observational study |
| #4 | 2023 | Acute extensive and advanced stage Marjolin's ulcer arising within a below knee amputation stump ulcer in a middle-aged man, a case report and review of literature | 10.1007/s00428-023-03602-w | A case report |
| #5 | 2021 | Addressing the liver progenitor cell response and hepatic oxidative stress in experimental non-alcoholic fatty liver disease/non-alcoholic steatohepatitis using amniotic epithelial cells | 10.1186/s13287-021-02476-6 | Not focused on STING pathway mechanisms |
| #6 | 2023 | Beyond DNA sensing: expanding the role of cGAS/STING in immunity and diseases | 10.1007/s12272-023-01452-3 | Review of literature |
| #7 | 2022 | Can the cGAS-STING Pathway Play a Role in the Dry Eye? | 10.3389/fimmu.2022.929230 | Unrelated to liver disease study |
| #8 | 2021 | cGAS-STING Signaling Pathway and Liver Disease: From Basic Research to Clinical Practice | 10.3389/fphar.2021.719644 | Unrelated to liver disease study |
| #9 | 2021 | cGAS–STING signaling and function in metabolism and kidney diseases | 10.1093/jmcb/mjab066 | Unrelated to liver disease study |
| #10 | 2021 | Circulatory endothelin 1-regulating rnas panel: Promising biomarkers for non-invasive nafld/nash diagnosis and stratification: Clinical and molecular pilot study | 10.3390/genes12111813 | Clinical and molecular pilot study |
| #11 | 2020 | COVID-19 as a STING disorder with delayed over-secretion of interferon-beta | 10.1016/j.ebiom.2020.102801 | Unrelated to liver disease study |
| #12 | 2017 | De novo mutation in ACACB in childhood onset SLE highlights a novel role as modulator of nucleic acid sensor-driven type I interferon responses |  | Not focused on STING pathway mechanisms |
| #13 | 2022 | Expanding role of deoxyribonucleic acid-sensing mechanism in the development of lifestyle-related diseases | 10.3389/fcvm.2022.881181 | Not focused on STING pathway mechanisms |
| #14 | 2023 | Expression of STING in Women with Morbid Obesity and Nonalcoholic Fatty Liver Disease | 10.3390/metabo13040496 | Not focused on STING pathway mechanisms |
| #15 | 2016 | Foreca sting lifetime health outcomes and costs of treatment for non-alcoholic fatty liver disease |  | Not focused on STING pathway mechanisms |
| #16 | 2022 | Function and regulation of ULK1: From physiology to pathology | 10.1016/j.gene.2022.146772 | Not focused on STING pathway mechanisms |
| #17 | 2021 | Hepatic stellate cell senescence in liver fibrosis: Characteristics, mechanisms and perspectives | 10.1016/j.mad.2021.111572 | Not focused on STING pathway mechanisms |
| #18 | 2022 | Increased serum cystatin C levels and responses of pancreatic α- and β-cells in type 2 diabetes |  | Unrelated to liver disease study |
| #19 | 2021 | Innate Immunity in Diabetic Wound Healing: Focus on the Mastermind Hidden in Chronic Inflammatory | 10.3389/fphar.2021.653940 | Not focused on STING pathway mechanisms |
| #20 | 2014 | Innate signaling in the inflammatory immune disorders | 10.1016/j.cytogfr.2014.06.003 | Review of literature |
| #21 | 2021 | Involvement of STING signaling pathway in non-alcoholic fatty liver disease | 10.11569/wcjd.v29.i24.1396 | Non-experimental or observational study |
| #22 | 2023 | Kounis Syndrome: A Sting to the Heart | 10.14503/THIJ-21-7788 | Review of literature |
| #23 | 2023 | LOSS OF OVARIAN HORMONE EXACERBATES DIETINDUCED NAFLD | 10.1097/HEP.0000000000000580 | Not focused on STING pathway mechanisms |
| #24 | 2018 | Macrophages Steal STING From the Infectious Disease Playbook to Promote Nonalcoholic Fatty Liver Disease | 10.1053/j.gastro.2018.11.009 | Review or commentary |
| #25 | 2024 | Mechanistic Study of Glycyrrhizic Acid Improving Alcoholic Fatty Liver Disease by Modulating the SHP1/SYK Signaling Pathway in Macrophages |  | Not focused on STING pathway mechanisms |
| #26 | 2019 | Mitochondrial DNA in liver inflammation and oxidative stress | 10.1016/j.lfs.2019.05.020 | Not focused on STING pathway mechanisms |
| #27 | 2023 | Mitochondrial DNA-triggered innate immune response: mechanisms and diseases | 10.1038/s41423-023-01086-x | Review of literature |
| #28 | 2021 | Molecular mechanisms of mtdna-mediated inflammation | 10.3390/cells10112898 | Review of literature |
| #29 | 2022 | Multifaceted functions of STING in human health and disease: from molecular mechanism to targeted strategy | 10.1038/s41392-022-01252-z | Review of literature |
| #30 | 2023 | Normalization of hepatic ChREBP activity does not protect against liver disease progression in a mouse model for Glycogen Storage Disease type Ia | 10.1186/s40170-023-00305-3 | Not focused on STING pathway mechanisms |
| #31 | 2023 | P62, a multifunctional regulator in chronic liver diseases: a review | 10.3867/j.issn.1000-3002.2023.02.009 | Review of literature |
| #32 | 2022 | Polyploidy, DNA damage response driver or gatekeeper of chronic liver diseases | 10.1158/1557-3265.LIVERCA22-IA03 | Review of literature |
| #33 | 2023 | Potential Therapeutic Value of the STING Inhibitors | 10.3390/molecules28073127 | Review of literature |
| #34 | 2023 | REDUCED INFLAMMATORY POTENTIAL OF TUMOR ASSOCIATED MACROPHAGES PROMOTES T CELL TOLERANCE AND EXHAUSTION IN HUMAN HEPATOCELLULAR CARCINOMA | 10.1097/HEP.0000000000000580 | Not focused on STING pathway mechanisms |
| #35 | 2023 | Research progress in cGAS/STING pathway in metabolic inflammation | 10.3867/j.issn.1000-3002.2023.10.010 | Review of literature |
| #36 | 2022 | Role of the cGAS–STING pathway in systemic and organ-specific diseases | 10.1038/s41581-022-00589-6 | Review of literature |
| #37 | 2021 | Sars-cov-2 induces strong inflammation in organoids derived directly from cirrhotic nash patient liver but not healthy donor liver. | 10.1002/hep.32187 | Not focused on STING pathway mechanisms |
| #38 | 2021 | STING and liver disease | 10.1007/s00535-021-01803-1 | Review of literature |
| #39 | 2021 | STING-dependent induction of lipid peroxidation mediates intestinal ischemia-reperfusion injury | 10.1016/j.freeradbiomed.2020.12.010 | Unrelated to liver disease study |
| #40 | 2019 | The cGAS-cGAMP-STING pathway: A molecular link between immunity and metabolism |  | Review of literature |
| #41 | 2021 | The cGAS-STING Pathway: Novel Perspectives in Liver Diseases | 10.3389/fimmu.2021.682736 | Review of literature |
| #42 | 2021 | The cGAS–STING pathway as a therapeutic target in inflammatory diseases | 10.1038/s41577-021-00524-z | Review of literature |
| #43 | 2022 | The cGAS–STING signaling in cardiovascular and metabolic diseases: Future novel target option for pharmacotherapy | 10.1016/j.apsb.2021.05.011 | Review of literature |
| #44 | 2022 | The contribution of sterile inflammation to the fatty liver disease and the potential therapies | 10.1016/j.biopha.2022.112789 | Review of literature |
| #45 | 2021 | The Cytosolic DNA-Sensing cGAS-STING Pathway in Liver Diseases | 10.3389/fcell.2021.717610 | Review of literature |
| #46 | 2024 | The dual function of cGAS-STING signaling axis in liver diseases | 10.1038/s41401-023-01220-5 | Review of literature |
| #47 | 2021 | The role of cGAS-STING signalling in liver diseases | 10.1016/j.jhepr.2021.100324 | Review of literature |
| #48 | 2024 | The Role of cGAS-STING Signalling in Metabolic Diseases: from Signalling Networks to Targeted Intervention | 10.7150/ijbs.84890 | Review of literature |
| #49 | 2022 | The STING in Non-Alcoholic Fatty Liver Diseases: Potential Therapeutic Targets in Inflammation-Carcinogenesis Pathway | 10.3390/ph15101241 | Review of literature |
| #50 | 2020 | Traditional Use of Medicinal Plants in South-Eastern Serbia (Pčinja District): Ethnopharmacological Investigation on the Current Status and Comparison With Half a Century Old Data | 10.3389/fphar.2020.01020 | Not focused on STING pathway mechanisms |
| #51 | 2023 | Updated roles of cGAS-STING signaling in autoimmune diseases | 10.3389/fimmu.2023.1254915 | Review of literature |

| **Supplementary Table 11-2.** List of excluded studies in Hepatitis. | | | | |
| --- | --- | --- | --- | --- |
| Item | Year | Title | DOI | Reason for exclusion |
| #1 | 2014 | 97% sustained virologic response in Japanese patients with chronic genotype 2 hepatitis C virus infection receiving sofosbuvir in combination with ribavirin for 12 weeks: Results from a phase 3 multicenter study | 10.1002/hep.27516 | Non-experimental or observational study |
| #2 | 2019 | A case of novel identified proteasome-related autoinflammation and immunodeficiency syndrome caused by PSMB9 mutation | 10.1186/s12969-019-0313-x | A case report |
| #3 | 2020 | A four-chemokine signature is associated with a T-cell- inflamed phenotype in primary and metastatic pancreatic cancer | 10.1158/1078-0432.CCR-19-2803 | Non-experimental or observational study |
| #4 | 2019 | A new hepatoma cell line exhibiting high susceptibility to hepatitis B virus infection | 10.1016/j.bbrc.2019.05.126 | Not focused on STING pathway mechanisms |
| #5 | 2020 | A Potential Role for Mitochondrial DNA in the Activation of Oxidative Stress and Inflammation in Liver Disease | 10.1155/2020/5835910 | Review of literature |
| #6 | 2021 | A STING-related prognostic score predicts high-risk patients of colorectal cancer and provides insights into immunotherapy | 10.21037/atm-20-2430 | Non-experimental or observational study |
| #7 | 2023 | Activation of CD4 T cells during prime immunization determines the success of a therapeutic hepatitis B vaccine in HBV-carrier mouse models | 10.1016/j.jhep.2022.12.013 | Not focused on STING pathway mechanisms |
| #8 | 2015 | Advax™, a novel microcrystalline polysaccharide particle engineered from delta inulin, provides robust adjuvant potency together with tolerability and safety | 10.1016/j.vaccine.2015.09.030 | Not focused on STING pathway mechanisms |
| #9 | 2013 | Alternative splicing variants of STING present a differential ability inhibiting human wild type STING | 10.1089/hum.2013.2513 | Non-experimental or observational study |
| #10 | 2020 | APOL1-Associated Collapsing Focal Segmental Glomerulosclerosis in a Patient With Stimulator of Interferon Genes (STING)-Associated Vasculopathy With Onset in Infancy (SAVI) | 10.1053/j.ajkd.2019.07.010 | Unrelated to liver disease study |
| #11 | 2020 | Balancing STING in antimicrobial defense and autoinflammation | 10.1016/j.cytogfr.2020.06.004 | Review of literature |
| #12 | 2023 | Blocking Tim-3 enhances the anti-tumor immunity of STING agonist ADU-S100 by unleashing CD4+ T cells through regulating type 2 conventional dendritic cells | 10.7150/thno.86792 | Not focused on STING pathway mechanisms |
| #13 | 2023 | Carbon ion irradiation induces DNA damage in melanoma and optimizes the tumor microenvironment based on the cGAS–STING pathway | 10.1007/s00432-023-04577-6 | Unrelated to liver disease study |
| #14 | 2015 | Casein kinase II controls TBK1/IRF3 activation in IFN response against viral infection | 10.4049/jimmunol.1402777 | Not focused on STING pathway mechanisms |
| #15 | 2013 | Cell type-specific subcellular localization of phospho- TBK1 in response to cytoplasmic viral DNA | 10.1371/journal.pone.0083639 | Non-experimental or observational study |
| #16 | 2021 | cGAS-STING Signaling Pathway and Liver Disease: From Basic Research to Clinical Practice | 10.3389/fphar.2021.719644 | Review of literature |
| #17 | 2020 | Comparison of mRNA Levels of Stimulator of Interferon Genes (STING) in Individuals with Natural Immunity to Hepatitis B Virus (HBV), and in those with Chronic Hepatitis B Infection and without HBV | 10.5578/mb.68787 | Non-experimental or observational study |
| #18 | 2013 | Conference Scene: Novelties in immunotherapy | 10.2217/imt.13.109 | Conference |
| #19 | 2022 | Congenital tremor and splay leg in piglets – insights into the virome, local cytokine response, and histology | 10.1186/s12917-022-03443-w | Unrelated to liver disease study |
| #20 | 2018 | Correction: "Hepatitis C virus NS4B can suppress STING accumulation to evade innate immune responses" [Journal of Virology, 90, 1, (2016) (254-265)] doi 10.1128/JVI.01720-15 | 10.21037/atm-21-491 | Secondary literature or commentary |
| #21 | 2021 | Current status of intralesional agents in treatment of malignant melanoma | 10.21037/atm-21-491 | Unrelated to liver disease |
| #22 | 2021 | Cyclic Guanosine Monophosphate–Adenosine Monophosphate Synthase (cGAS), a Multifaceted Platform of Intracellular DNA Sensing | 10.3389/fimmu.2021.637399 | Review of literature |
| #23 | 2015 | Decreased expressions of STING but not IRF3 molecules in chronic HBV infected patients |  | Non-experimental or observational study |
| #24 | 2021 | Defective clearance of nucleic acids exacerbates AKI |  | Unrelated to liver disease |
| #25 | 2021 | Delivery of STING agonists for adjuvanting subunit vaccines | 10.1016/j.addr.2021.114020 | Unrelated to liver disease |
| #26 | 2010 | Diagnostic protocol of pruritus | 10.1016/S0304-5412(10)70007-X | Unrelated to liver disease |
| #27 | 2013 | Different Genetic Associations of the IgE Production among Fetus, Infancy and Childhood | 10.1371/journal.pone.0070362 | Unrelated to liver disease |
| #28 | 2017 | Disease-associated mutations identify a novel region in human STING necessary for the control of type I interferon signaling | 10.1016/j.jaci.2016.10.031 | Non-experimental or observational study |
| #29 | 2018 | DNA damaging agents and immunotherapy in NSCLC: Is there a STING in the tale? | 10.1093/annonc/mdy269.140 | Non-experimental or observational study |
| #30 | 2013 | DNA vaccines: A simple DNA sensing matter? | 10.4161/hv.25893 | Review of literature |
| #31 | 2023 | DUSP1 protects against ischemic acute kidney injury through stabilizing mtDNA via interaction with JNK | 10.1038/s41419-023-06247-4 | Unrelated to liver disease |
| #32 | 2022 | Editorial: The Regulation of Autophagy Activity in Pathogenic Microorganism Infection | 10.3389/fcell.2022.889283 | Secondary literature or commentary |
| #33 | 2023 | Editorial: Viruses, innate immunity, and antiviral strategies: from basic research to clinical applications | 10.3389/fcimb.2023.1268363 | Secondary literature or commentary |
| #34 | 2019 | Edwardsiella tarda Bacteremia in Untreated Hepatitis C: Alterations in Antimicrobial Therapy for a Pan-Susceptible Pathogen in a Critically Ill Patient | 10.1097/MJT.0000000000000958 | Not focused on STING pathway mechanisms |
| #35 | 2021 | Effect of HBsAg on the production of interferon - α in peripheral blood plasmacytoid dendritic cells induced by the stimulator of interferon genes signaling pathway | 10.3969/j.issn.1001-5256.2021.06.016 | Non-experimental or observational study |
| #36 | 2021 | Endoplasmic reticulum & mitochondrial calcium homeostasis: The interplay with viruses | 10.1016/j.mito.2021.03.008 | Review of literature |
| #37 | 2018 | Exploration of the cGAS-STING pathway in prostate cancer | 10.1200/jco.2018.36.15_suppl.5075 | Unrelated to liver disease |
| #38 | 2022 | Floats Like a Butterfly, Stings Like a Bee: Lupus Nephritis |  | Unrelated to liver disease |
| #39 | 2023 | Function and regulation of cGAS-STING signaling in infectious diseases | 10.3389/fimmu.2023.1130423 | Review of literature |
| #40 | 2022 | Function and regulation of ULK1: From physiology to pathology | 10.1016/j.gene.2022.146772 | Review of literature |
| #41 | 2013 | GeoSentinel surveillance of illness in returned travelers, 2007-2011 | 10.7326/0003-4819-158-6-201303190-00005 | Non-experimental or observational study |
| #42 | 2015 | HBV induce cytosolic DNA sensor cGAS but suppress IRF3 to prevent generation of antiviral defense mechanism in infected cell | 10.1016/j.jceh.2015.07.008 | Non-experimental or observational study |
| #43 | 2014 | HBV life cycle is restricted in mouse hepatocytes expressing human NTCP | 10.1038/cmi.2013.66 | Non-experimental or observational study |
| #44 | 2016 | HBV reduces IRF3 gene expression despite increased expression of cellular cytosolic DNA sensor cGAS | 10.1007/s12072-016-9707-8 | Non-experimental or observational study |
| #45 | 2024 | HCV-induced autophagy and innate immunity | 10.3389/fimmu.2024.1305157 | Review of literature |
| #46 | 2011 | Hepatitis B reactivation complicated with nephrotic syndrome in association with venom immunotherapy:Need for preemptive treatment? | 10.1007/s12072-010-9241-z | A case report |
| #47 | 2020 | Hepatitis B Virus Might Be Sensed by STING-Dependent DNA Sensors and Attenuates the Response of STING-Dependent DNA Sensing Pathway in Humans with Acute and Chronic Hepatitis B Virus Infection | 10.1089/vim.2020.0096 | Non-experimental or observational study |
| #48 | 2021 | Hepatitis B virus nucleocapsid uncoating: biological consequences and regulation by cellular nucleases | 10.1080/22221751.2021.1919034 | Not focused on STING pathway mechanisms |
| #49 | 2022 | How SARS-CoV-2 dodges immune surveillance and facilitates infection: an analytical review | 10.1080/14787210.2022.2078307 | Review of literature |
| #50 | 2022 | HSP Triggered by Wasp String Presents as Acute GI Bleeding | 10.14309/01.ajg.0000867076.95974.1b | Not focused on STING pathway mechanisms |
| #51 | 2015 | Human endogenous retrovirus expression is inversely related with the up-regulation of interferon-inducible genes in the skin of patients with lichen planus | 10.1007/s00403-014-1524-0 | Unrelated to liver disease |
| #52 | 2004 | Humanopathogenic parasites - An unwelcome import |  | Not focused on STING pathway mechanisms |
| #53 | 2024 | Hypermethylation of the glutathione peroxidase 4 gene promoter is associated with the occurrence of immune tolerance phase in chronic hepatitis B | 10.1186/s12985-024-02346-6 | Non-experimental or observational study |
| #54 | 2023 | ICI-based therapies: A new strategy for oral potentially malignant disorders | 10.1016/j.oraloncology.2023.106388 | Review of literature |
| #55 | 2018 | Identification of a high-risk subgroup in primary prostate cancers presenting with targetable immune biology | 10.1158/1538-7445.AM2018-283 | Non-experimental or observational study |
| #56 | 2021 | Immunity and viral infections: Modulating antiviral response via crispr–cas systems | 10.3390/v13071373 | Review of literature |
| #57 | 2018 | Immunotherapy, an evolving approach for the management of triple negative breast cancer: Converting non-responders to responders | 10.1016/j.critrevonc.2018.01.005 | Review of literature |
| #58 | 2024 | In situ bio-mineralized Mn nanoadjuvant enhances anti-influenza immunity of recombinant virus-like particle vaccines | 10.1016/j.jconrel.2024.02.027 | Unrelated to liver disease |
| #59 | 2019 | Induction of humoral and cellular immune response to HBV vaccine can be up-regulated by STING ligand | 10.1016/j.virol.2019.03.013 | Non-experimental or observational study |
| #60 | 2019 | Interferon signaling is diminished with age and is associated with immune checkpoint blockade efficacy in triple-negative breast cancer | 10.1158/2159-8290.CD-18-1454 | Not focused on STING pathway mechanisms |
| #61 | 2023 | Interplay between RNA viruses and cGAS/STING axis in innate immunity | 10.3389/fcimb.2023.1172739 | Unrelated to liver disease |
| #62 | 2019 | Intralesional Cancer Immunotherapies | 10.1016/j.hoc.2018.12.009 | Non-experimental or observational study |
| #63 | 2017 | Investigation of intracellular innate immune responses to AAV gene therapy in the retina |  | Unrelated to liver disease |
| #64 | 2018 | UnreKidney Week 2018lated to liver disease |  | Review of literature |
| #65 | 2022 | Macrophage-Specific Deletion of Cytosolic Nucleotide Sensors Ameliorate Kidney Fibrosis and Inflammation |  | Unrelated to liver disease |
| #66 | 2021 | Mechanisms of immune escape and resistance to checkpoint inhibitor therapies in mismatch repair deficient metastatic colorectal cancers | 10.3390/cancers13112638 | Unrelated to liver disease |
| #67 | 2014 | Message in a bottle: lessons learned from antagonism of STING signalling during RNA virus infection | 10.1016/j.cytogfr.2014.08.004 | Secondary literature or commentary |
| #68 | 2022 | Metabolic reprogramming and immune regulation in viral diseases | 10.1002/rmv.2268 | Review of literature |
| #69 | 2018 | Methylation status of the stimulator of interferon genes promoter in patients with chronic hepatitis B | 10.1097/MD.0000000000013904 | Non-experimental or observational study |
| #70 | 2017 | MITA/STING and its alternative splicing isoform MRP restrict hepatitis B virus replication | 10.1371/journal.pone.0169701 | Not focused on STING pathway mechanisms |
| #71 | 2019 | Mitochondrial DNA in liver inflammation and oxidative stress | 10.1016/j.lfs.2019.05.020 | Review of literature |
| #72 | 2022 | Mobilizing phospholipids on tumor plasma membrane implicates phosphatidylserine externalization blockade for cancer immunotherapy | 10.1016/j.celrep.2022.111582 | Unrelated to liver disease |
| #73 | 2020 | Molecular Underpinnings of Severe Coronavirus Disease 2019 | 10.1001/jama.2020.14015 | Review of literature |
| #74 | 2017 | Morbidity among Israeli paediatric travellers | 10.1093/jtm/tax062 | Review of literature |
| #75 | 2022 | Multiplex immunohistochemistry defines the tumor immune microenvironment and immunotherapeutic outcome in CLDN18.2-positive gastric cancer | 10.1186/s12916-022-02421-1 | Unrelated to liver disease |
| #76 | 2021 | NAD metabolism modulates inflammation and mitochondria function in diabetic kidney disease | 10.1101/2021.12.05.471273 | Unrelated to liver disease |
| #77 | 2005 | Narrative review: Diseases that masquerade as infectious cellulitis | 10.7326/0003-4819-142-1-200501040-00011 | Review of literature |
| #78 | 2022 | Neoantigen Immunotherapeutic-Gel Combined with TIM-3 Blockade Effectively Restrains Orthotopic Hepatocellular Carcinoma Progression | 10.1021/acs.nanolett.1c04977 | Not focused on STING pathway mechanisms |
| #79 | 2020 | New approaches to the treatment of chronic hepatitis B | 10.3390/jcm9103187 | Review of literature |
| #80 | 2023 | Non-small cell lung cancers (NSCLCs) oncolysis using coxsackievirus B5 and synergistic DNA-damage response inhibitors | 10.1038/s41392-023-01603-4 | Unrelated to liver disease |
| #81 | 2020 | Nucleic Acid Sensors as Therapeutic Targets for Human Disease | 10.1016/j.immuni.2020.04.004 | Non-experimental or observational study |
| #82 | 2011 | Ocimum sanctum linn (TULSI) - an overview |  | Review of literature |
| #83 | 2021 | PCV2 targets cGAS to inhibit type I interferon induction to promote other DNA virus infection | 10.1371/journal.ppat.1009940 | Not focused on STING pathway mechanisms |
| #84 | 2010 | Pegylated interferon and ribavirin treatment for children with hepatitis C | 10.1097/01.mpg.0000383075.98243.67 | Non-experimental or observational study |
| #85 | 2019 | Pharmacological modulation of nucleic acid sensors — therapeutic potential and persisting obstacles | 10.1038/s41573-019-0043-2 | Review of literature |
| #86 | 2003 | Population-based study of non-Hodgkin lymphoma, histology, and medical history among human immunodeficiency virus-negative participants in San Francisco | 10.1093/aje/kwg145 | Non-experimental or observational study |
| #87 | 2022 | POS-134 NEW ONSET NEPHROTIC SYNDROME FOLLOWING INACTIVATED SARS-CoV-2 VACCINE | 10.1016/j.ekir.2022.01.146 | A case report |
| #88 | 2022 | Post-Translational Modifications of cGAS-STING: A Critical Switch for Immune Regulation | 10.3390/cells11193043 | Non-experimental or observational study |
| #89 | 2021 | Potential of E3 ubiquitin ligases in cancer immunity: Opportunities and challenges | 10.3390/cells10123309 | Review of literature |
| #90 | 2020 | Precision medicine for squamous cell cancer of the head and neck | 10.2340/00015555-3586 | Unrelated to liver disease |
| #91 | 2022 | Predicting response to immunotherapy in gastric cancer via multi-dimensional analyses of the tumour immune microenvironment | 10.1038/s41467-022-32570-z | Unrelated to liver disease |
| #92 | 2023 | PTEN-induced kinase 1 is associated with renal aging, via the cGAS-STING pathway | 10.1111/acel.13865 | Unrelated to liver disease |
| #93 | 2010 | Quartan malaria-associated childhood nephrotic syndrome: Now a rare clinical entity in malaria endemic Nigeria | 10.1093/ndt/gfp536 | Unrelated to liver disease |
| #94 | 2023 | REDUCED INFLAMMATORY POTENTIAL OF TUMOR ASSOCIATED MACROPHAGES PROMOTES T CELL TOLERANCE AND EXHAUSTION IN HUMAN HEPATOCELLULAR CARCINOMA | 10.1097/HEP.0000000000000580 | Not focused on STING pathway mechanisms |
| #95 | 2011 | Retransplantation for recurrent hepatitis A virus infection following liver transplantation for fulminant hepatitis A infection | 10.1002/lt.22457 | A case report |
| #96 | 2022 | RNA viruses and the cGAS-STING pathway: reframing our understanding of innate immune sensing | 10.1016/j.coviro.2022.101206 | Review of literature |
| #97 | 2019 | rt269I Type of Hepatitis B Virus (HBV) Leads to HBV e Antigen Negative Infections and Liver Disease Progression via Mitochondrial Stress Mediated Type I Interferon Production in Chronic Patients With Genotype C Infections | 10.3389/fimmu.2019.01735 | Non-experimental or observational study |
| #98 | 2023 | Senotherapeutics: An emerging approach to the treatment of viral infectious diseases in the elderly | 10.3389/fcimb.2023.1098712 | Unrelated to liver disease |
| #99 | 2023 | Sex Differences in Genomic Features of Hepatitis B–Associated Hepatocellular Carcinoma With Distinct Antitumor Immunity | 10.1016/j.jcmgh.2022.10.009 | Not focused on STING pathway mechanisms |
| #100 | 2017 | Skin diseases and sexually transmitted infection in a Hungarian prison | 10.1097/MRM.0000000000000087 | Unrelated to liver disease |
| #101 | 2019 | Small-molecule immuno-oncolog therapy: Advances, challenges and new directions | 10.2174/1568026619666190308131805 | Unrelated to liver disease |
| #102 | 2023 | Spatial proteomics identifies a spectrum of immune dysregulation in acquired bone marrow failure syndromes | 10.3389/fimmu.2023.1213560 | Unrelated to liver disease |
| #103 | 2023 | Species-specific cleavage of cGAS by picornavirus protease 3C disrupts mitochondria DNA-mediated immune sensing | 10.1371/journal.ppat.1011641 | Unrelated to liver disease |
| #104 | 2019 | STING activation reprograms tumor vasculatures and synergizes with VEGFR2 blockade | 10.1172/JCI125413 | Unrelated to liver disease |
| #105 | 2021 | STING and liver disease | 10.1007/s00535-021-01803-1 | Review of literature |
| #106 | 2019 | STING signaling and host defense against microbial infection | 10.1038/s12276-019-0333-0 | Review of literature |
| #107 | 2018 | STING signaling: A key to therapeutic tumor immunity | 10.2217/imt-2018-0064 | Review of literature |
| #108 | 2017 | Superior immunogenicity of HCV envelope glycoproteins when adjuvanted with cyclic-di-AMP, a STING activator or archaeosomes | 10.1016/j.vaccine.2017.10.072 | Not focused on STING pathway mechanisms |
| #109 | 2023 | Swertia chirayita: A comprehensive review on traditional uses, phytochemistry, quality assessment and pharmacology | 10.1016/j.jep.2022.115714 | Review of literature |
| #110 | 2019 | Targeting DNA damage response promotes antitumor immunity through STING-mediated T-cell activation in small cell lung cancer | 10.1158/2159-8290.CD-18-1020 | Unrelated to liver disease |
| #111 | 2021 | The adjuvanticity of manganese for microbial vaccines via activating the IRF5 signaling pathway | 10.1016/j.bcp.2021.114720 | Non-experimental or observational study |
| #112 | 2006 | The body of knowledge for the practice of travel medicine - 2006 | 10.1111/j.1708-8305.2006.00054.x | Review of literature |
| #113 | 2024 | The cGAS-STING pathway in viral infections: a promising link between inflammation, oxidative stress and autophagy | 10.3389/fimmu.2024.1352479 | Review of literature |
| #114 | 2021 | The cGAS-STING Pathway: Novel Perspectives in Liver Diseases | 10.3389/fimmu.2021.682736 | Review of literature |
| #115 | 2019 | The common costimulatory and coinhibitory signaling molecules in head and neck squamous cell carcinoma | 10.3389/fimmu.2019.02457 | Unrelated to liver disease |
| #116 | 2021 | The complex role of AIM2 in autoimmune diseases and cancers | 10.1002/iid3.443 | Unrelated to liver disease |
| #117 | 2021 | The Cytosolic DNA-Sensing cGAS-STING Pathway in Liver Diseases | 10.3389/fcell.2021.717610 | Review of literature |
| #118 | 2022 | The Evolutionary Dance between Innate Host Antiviral Pathways and SARS-CoV-2 | 10.3390/pathogens11050538 | Unrelated to liver disease |
| #119 | 2014 | The global burden of myocarditis: Part 1: A systematic literature review for the global burden of diseases, injuries, and risk factors 2010 study | 10.1016/j.gheart.2014.01.007 | Review of literature |
| #120 | 2021 | The inhibitory receptor TIM-3 limits activation of the cGAS-STING pathway in intra-tumoral dendritic cells by suppressing extracellular DNA uptake | 10.1016/j.immuni.2021.04.019 | Unrelated to liver disease |
| #121 | 2023 | THE IODINATED FLUORESCEIN DERIVATIVE PV-10 ENHANCES THE ANTIVIRAL ACTIVITY OF CD8+ T-CELLS BY INDUCING STING DIMERIZATION: IMPLICATIONS FOR ENHANCED VACCINE APPLICATIONS | 10.1136/jitc-2023-SITC2023.1118 | Unrelated to liver disease |
| #122 | 2021 | The multiple potential biomarkers for predicting immunotherapy response-finding the needle in the haystack | 10.1136/jitc-2023-SITC2023.1118 | Non-experimental or observational study |
| #123 | 2009 | The regulation of innate immune signaling by HTLV-1 tax | 10.1089/aid.2009.9993 | Unrelated to liver disease |
| #124 | 2019 | The Role of Deubiquitinases in Oncovirus and Host Interactions | 10.1155/2019/2128410 | Review of literature |
| #125 | 2019 | The Role of Nucleic Acid Sensing in Controlling Microbial and Autoimmune Disorders | 10.1016/bs.ircmb.2018.08.002 | Not focused on STING |
| #126 | 2022 | Therapeutic Advances in Viral Hepatitis A–E | 10.1007/s12325-022-02070-z | Review of literature |
| #127 | 2018 | Transcriptome profiling of hepatitis B virus-infected human hepatocyte derived from chimeric mice with humanized liver |  | Non-experimental or observational study |
| #128 | 2017 | Transmissible Gastroenteritis Virus Papain-Like Protease 1 Antagonizes Production of Interferon- β through Its Deubiquitinase Activity | 10.1155/2017/7089091 | Unrelated to liver disease |
| #129 | 2002 | Traveling to New Zealand | 10.2310/7060.2002.24587 | Unrelated to liver disease |
| #130 | 2015 | Treatment of chronic hepatitis B with pattern recognition receptor agonists: Current status and potential for a cure | 10.1016/j.antiviral.2015.07.006 | Review of literature |
| #131 | 2021 | UNC93B1 curbs cytosolic DNA signaling by promoting STING degradation | 10.1002/eji.202048901 | Unrelated to liver disease |
| #132 | 2022 | Urolithin A Attenuates Hyperuricemic Nephropathy in Fructose-Fed Mice by Impairing STING-NLRP3 Axis-Mediated Inflammatory Response via Restoration of Parkin-Dependent Mitophagy | 10.3389/fphar.2022.907209 | Unrelated to liver disease |
| #133 | 2021 | Viperin binds STING and enhances the type-I interferon response following dsDNA detection | 10.1111/imcb.12420 | Unrelated to liver disease |
| #134 | 2016 | Viral hepatitis: A lack of hepatocyte STING favours HBV infection | 10.1038/nrgastro.2016.111 | Review of literature |
| #135 | 2012 | Wedelia chinenis (Asteraceae) - An overview | 10.1016/S2221-1691(12)60380-3 | Unrelated to liver disease |
| #136 | 2023 | Yi-Shen-Xie-Zhuo formula alleviates cisplatin-induced AKI by regulating inflammation and apoptosis via the cGAS/STING pathway | 10.1016/j.jep.2023.116327 | Unrelated to liver disease |

| **Supplementary Table 11-3.** List of excluded studies in Liver Neoplasms. | | | | |
| --- | --- | --- | --- | --- |
| Item | Year | Title | DOI | Reason for exclusion |
| #1 | 2023 | 704/DNA vaccines leverage cytoplasmic DNA stimulation to promote anti-HIV neutralizing antibody production in mice and strong immune response against alpha-fetoprotein in non-human primates | 10.1016/j.omtn.2023.04.029 | Not focused on STING pathway mechanisms |
| #2 | 2023 | A Booster for Radiofrequency Ablation: Advanced Adjuvant Therapy via In Situ Nanovaccine Synergized with Anti-programmed Death Ligand 1 Immunotherapy for Systemically Constraining Hepatocellular Carcinoma | 10.1021/acsnano.3c08064 | Non-experimental or observational study |
| #3 | 2023 | A LISTERIOLYSIN O-CD47 PROTEIN-ANTIBODY CONJUGATE TRIGGERS CGAS-STING ACTIVATION IN SOLID TUMOR MALIGNANCIES | 10.1136/jitc-2023-SITC2023.1148 | Not focused on STING pathway mechanisms |
| #4 | 2023 | A systemically administered killed bacteriabased multiple immune receptor agonist for pulsed antitumor immunotherapy | 10.1158/1538-7445.AM2023-4165 | Not focused on STING pathway mechanisms |
| #5 | 2022 | Blocking CD47 promotes antitumour immunity through CD103+ dendritic cell–NK cell axis in murine hepatocellular carcinoma model | 10.1016/j.jhep.2022.03.011 | Not focused on STING pathway mechanisms |
| #6 | 2024 | Blocking MARCO+ tumor-associated macrophages improves anti-PD-L1 therapy of hepatocellular carcinoma by promoting the activation of STING-IFN type I pathway | 10.1016/j.canlet.2023.216568 | Not focused on STING pathway mechanisms |
| #7 | 2022 | CFI-402257, a TTK inhibitor, effectively suppresses hepatocellular carcinoma | 10.1073/pnas.2119514119 | Not focused on STING pathway mechanisms |
| #8 | 2021 | cGAS-STING Signaling Pathway and Liver Disease: From Basic Research to Clinical Practice | 10.3389/fphar.2021.719644 | Review of literature |
| #9 | 2004 | Clinical Significance of Combination Study of Apoptotic Factors and Proliferating Cell Nuclear Antigen in Estimating the Prognosis of Hepatocellular Carcinoma | 10.1002/jso.20006 | Non-experimental or observational study |
| #10 | 2008 | Clinical significance of Serum HGF and c-Met expression in tumor tissue for evaluation of properties and treatment of hepatocellular carcinoma |  | Not focused on STING pathway mechanisms |
| #11 | 2007 | Clinicopathological features of hepatocellular carcinoma evaluated by vascular endothelial growth factor expression | 10.1111/j.1440-1746.2006.04790.x | Not focused on STING pathway mechanisms |
| #12 | 2021 | Envisioning the immune system to determine its role in pancreatic ductal adenocarcinoma: Culprit or victim? | 10.1016/j.imlet.2021.02.009 | Not focused on STING pathway mechanisms |
| #13 | 2008 | Hepatic fibrosis influences the growth of hepatocellular carcinoma |  | Non-experimental or observational study |
| #14 | 2020 | Identification of prognostic biomarkers and correlations with immune infiltrates among cGAS-STING in hepatocellular carcinoma | 10.1042/BSR20202603 | Non-experimental or observational study |
| #15 | 2022 | Identification of Therapeutic Targets and Prognostic Biomarkers among Genes from the Mediator Complex Family in the Hepatocellular Carcinoma Tumour-Immune Microenvironment | 10.1155/2022/2021613 | Non-experimental or observational study |
| #16 | 2023 | Injectable hydrogel loaded with lysed OK-432 and doxorubicin for residual liver cancer after incomplete radiofrequency ablation | 10.1186/s12951-023-02170-0 | Not focused on STING pathway mechanisms |
| #17 | 2022 | Irradiation Induced Activation of cGAS/STING Signaling Promotes Macrophage Anti-Tumor Activity via CXCL9, CXCL10-CXCR3 Axis | 10.1016/j.ijrobp.2022.07.643 | Unrelated to liver disease |
| #18 | 2022 | Neoantigen Immunotherapeutic-Gel Combined with TIM-3 Blockade Effectively Restrains Orthotopic Hepatocellular Carcinoma Progression | 10.1021/acs.nanolett.1c04977 | Not focused on STING pathway mechanisms |
| #19 | 2022 | Phase 1a/1b study design of the novel STING agonist, immune-stimulating antibodyconjugate (ISAC) TAK-500, with or without pembrolizumab in patients with advanced solid tumors | 10.1200/JCO.2022.40.16_suppl.TPS2690 | Non-experimental or observational study |
| #20 | 2021 | Platinum-based chemotherapy in combination with PD-1/PD-L1 inhibitors: preclinical and clinical studies and mechanism of action | 10.1080/17425247.2021.1825376 | Not focused on STING pathway mechanisms |
| #21 | 2022 | PP039 Inhibition of CAF-1 histone chaperone complex triggers cytosolic DNA and dsRNA sensing pathways and induces intrinsic immunity of hepatocellular carcinoma | 10.1016/j.esmoop.2022.100725 | Not focused on STING pathway mechanisms |
| #22 | 2023 | PRELIMINARY RESULTS OF AN IN PROGRESS, FIRST-INHUMAN PHASE 1 STUDY OF DECOY20, AN INTRAVENOUS, KILLED, MULTIPLE IMMUNE RECEPTOR AGONIST BACTERIAL PRODUCT IN PATIENTS WITH ADVANCED SOLID TUMORS | 10.1136/jitc-2023-SITC2023.782-E | Non-experimental or observational study |
| #23 | 2022 | Proteomics analysis of HCC tumors treated with Sorafenib | 10.1007/s12072-022-10337-4 | Non-experimental or observational study |
| #24 | 2023 | REDUCED INFLAMMATORY POTENTIAL OF TUMOR ASSOCIATED MACROPHAGES PROMOTES T CELL TOLERANCE AND EXHAUSTION IN HUMAN HEPATOCELLULAR CARCINOMA | 10.1097/HEP.0000000000000580 | Not focused on STING pathway mechanisms |
| #25 | 2023 | Resistance to tyrosine kinase inhibitor confers an immunosuppressive microenvironment and crossresistance to immunotherapy through AXL/PDPK1 axis in liver cancer | 10.1158/1538-7445.AM2023-395 | Not focused on STING pathway mechanisms |
| #26 | 2022 | Role of hypofractionated radiotherapy in multidisciplinary approach for HCC: practical results and mechanism analysis | 10.1159/000528570 | Not focused on STING pathway mechanisms |
| #27 | 2023 | Roles of cGAS-STING pathway in radiotherapy combined with immunotherapy for hepatocellular carcinoma | 10.1158/1535-7163.MCT-23-0373 | Review of literature |
| #28 | 2022 | STING pathway contributes to the prognosis of hepatocellular carcinoma and identification of prognostic gene signatures correlated to tumor microenvironment | 10.1186/s12935-022-02734-4 | Non-experimental or observational study |
| #29 | 2009 | Successful surgical resection for peritoneal implantation of hepatocellular carcinoma at the paracardial portion | 10.1155/2009/231854 | Not focused on STING pathway mechanisms |
| #30 | 2024 | Targeting AXL induces tumor-intrinsic immunogenic response in tyrosine kinase inhibitor-resistant liver cancer | 10.1038/s41419-024-06493-0 | Not focused on STING pathway mechanisms |
| #31 | 2021 | The cGAS-STING Pathway: Novel Perspectives in Liver Diseases | 10.3389/fimmu.2021.682736 | Review of literature |
| #32 | 2021 | The complex role of AIM2 in autoimmune diseases and cancers | 10.1002/iid3.443 | Unrelated to liver disease |
| #33 | 2018 | The mechanism of obesity-associated liver cancer through Toll-like receptor signaling and DNA sensing machinery | 10.1111/cas.13904 | Not focused on STING pathway mechanisms |
| #34 | 2022 | THE NOVEL TELOMERASE-DIRECTED TELOMERETARGETED ANTICANCER AGENT 6-THIO-DG (THIO) DEMONSTRATES POTENT ACTIVITY AND INDUCES ANTITUMOR IMMUNITY IN HEPATOCELLULAR CARCINOMA (HCC) MODELS | 10.1136/jitc-2022-SITC2022.0892 | Not focused on STING pathway mechanisms |
| #35 | 2020 | Tumor regression in a mouse model of hepatocellular carcinoma upon treatment with the sting agonist ALG-031048 | 10.1002/hep.31579 | Not focused on STING pathway mechanisms |
| #36 | 2019 | Vaccinia virus-mediated cancer immunotherapy: Cancer vaccines and oncolytics | 10.1186/s40425-018-0495-7 | Review of literature |
| #37 | 2021 | The cGAS–STING pathway: more than fighting against viruses and cancer | 10.1186/s13578-021-00724-z | Review of literature |
| #38 | 2014 | Insect bite-like reaction in a patient with chronic lymphocytic leukemia; [Insektenstichartige Reaktion bei einer Patientin mit chronisch lymphatischer Leukämie] | 10.1111/ddg.12326 | Not focused on STING pathway mechanisms |
| #39 | 2023 | Letter to the editor: Disrupted BRCA1-PALB2 interaction induces tumor immunosuppression and T-lymphocyte infiltration in HCC through cGAS-STING pathway | 10.1002/hep.32690 | Secondary literature or commentary |
| #40 | 2023 | Injectable hydrogel loaded with lysed OK-432 and doxorubicin for residual liver cancer after incomplete radiofrequency ablation | 10.1186/s12951-023-02170-0 | Not focused on STING pathway mechanisms |
| #41 | 2022 | Activation of STING in the pancreatic tumor microenvironment: A novel therapeutic opportunity | 10.1016/j.canlet.2022.215694 | Unrelated to liver disease |
| #42 | 2022 | CFI-402257, a TTK inhibitor, effectively suppresses hepatocellular carcinoma | 10.1073/pnas.2119514119 | Not focused on STING pathway mechanisms |
| #43 | 2019 | cGAMP inhibits tumor growth in colorectal cancer metastasis through the STING/STAT3 axis in a zebrafish xenograft model | 10.1016/j.fsi.2019.09.075 | Unrelated to liver disease |
| #44 | 2021 | cGAS-STING Signaling Pathway and Liver Disease: From Basic Research to Clinical Practice | 10.3389/fphar.2021.719644 | Review of literature |
| #45 | 2023 | cGAS-STING signaling pathway in intestinal homeostasis and diseases | 10.3389/fimmu.2023.1239142 | Review of literature |
| #46 | 2024 | cGAS-STING, inflammasomes and pyroptosis: an overview of crosstalk mechanism of activation and regulation | 10.1186/s12964-023-01466-w | Review of literature |
| #47 | 2009 | Co-localization of constituents of the dengue virus translation and replication machinery with amphisomes | 10.1099/vir.0.005355-0 | Not focused on STING pathway mechanisms |
| #48 | 2022 | Cocktail strategy based on a dual function nanoparticle and immune activator for effective tumor suppressive | 10.1186/s12951-022-01241-y | Not focused on STING pathway mechanisms |
| #49 | 2021 | Combination immunotherapies to overcome intrinsic resistance to checkpoint blockade in microsatellite stable colorectal cancer | 10.3390/cancers13194906 | Not focused on STING pathway mechanisms |
| #50 | 2023 | Comparative Efficacy and Safety of Chinese Patent Medicines for Cervical High-risk Human Papillomavirus Infection: A Bayesian Network Meta-Analysis | 10.7150/jca.86043 | Unrelated to liver disease |
| #51 | 2021 | Cuban blue scorpion venom and lung carcinoma: Is it always true what does not kill me makes me stronger? | 10.5152/turkthoracj.2021.20202 | Not focused on STING pathway mechanisms |
| #52 | 2020 | Cyclic GMP-AMP synthase is essential for cytosolic double-stranded DNA and fowl adenovirus serotype 4 triggered innate immune responses in chickens | 10.1016/j.ijbiomac.2020.01.015 | Unrelated to liver disease |
| #53 | 2022 | Design, synthesis and biological evaluation studies of novel small molecule ENPP1 inhibitors for cancer immunotherapy | 10.1016/j.bioorg.2021.105549 | Not focused on STING pathway mechanisms |
| #54 | 2023 | Development of Orally Bioavailable Amidobenzimidazole Analogues Targeting Stimulator of Interferon Gene (STING) Receptor | 10.1021/acs.jmedchem.2c02046 | Non-experimental or observational study |
| #55 | 2023 | Disrupted BRCA1-PALB2 interaction induces tumor immunosuppression and T-lymphocyte infiltration in HCC through cGAS-STING pathway | 10.1002/hep.32335 | Unrelated to liver disease |
| #56 | 2018 | DNA damage predicts prognosis and treatment response in colorectal liver metastases superior to immunogenic cell death and T cells | 10.7150/thno.24699 | Not focused on STING pathway mechanisms |
| #57 | 2021 | DNA sensing and associated type 1 interferon signaling contributes to progression of radiation-induced liver injury | 10.1038/s41423-020-0395-x | Not focused on STING pathway mechanisms |
| #58 | 2016 | Does the mosquito have more of a role in certain cancers than is currently appreciated? - The mosquito cocktail hypothesis | 10.1016/j.mehy.2015.12.005 | Not focused on STING pathway mechanisms |
| #59 | 2021 | E7766, a Macrocycle-Bridged Stimulator of Interferon Genes (STING) Agonist with Potent Pan-Genotypic Activity | 10.1002/cmdc.202100068 | Unrelated to liver disease |
| #60 | 2022 | Engaging Pattern Recognition Receptors in Solid Tumors to Generate Systemic Antitumor Immunity | 10.1007/978-3-030-96376-7_3 | Not focused on STING pathway mechanisms |
| #61 | 2021 | Envisioning the immune system to determine its role in pancreatic ductal adenocarcinoma: Culprit or victim? | 10.1016/j.imlet.2021.02.009 | Review of literature |
| #62 | 2004 | Epstein - Barr virus-associated extranodal NK/T-cell lymphoma following mosquito bites in an elderly patient without prior hypersensitivity | 10.1080/10428190410001697403 | A case report |
| #63 | 2022 | Extracellular matrix-degrading STING nanoagonists for mild NIR-II photothermal-augmented chemodynamic-immunotherapy | 10.1186/s12951-021-01226-3 | Unrelated to liver disease |
| #64 | 2017 | Fish TRIM32 functions as a critical antiviral molecule against iridovirus and nodavirus | 10.1016/j.fsi.2016.11.036 | Unrelated to liver disease |
| #65 | 2022 | Fluorophore-Conjugated Anti-ICOS Antibody Enables Precise Prediction of Therapeutic Response of the STING Agonist in Colorectal Cancer via NIRF Imaging | 10.1021/acs.molpharmaceut.2c00369 | Unrelated to liver disease |
| #66 | 2012 | Folk remedies of medicinal plants for snake bites, scorpion stings and dog bites in Eastern Ghats of Kolli Hills, Tamil Nadu, India | 10.7897/2277-4343.03523 | Not focused on STING pathway mechanisms |
| #67 | 2021 | Function and Regulation of Nuclear DNA Sensors During Viral Infection and Tumorigenesis | 10.3389/fimmu.2020.624556 | Not focused on STING pathway mechanisms |
| #68 | 2022 | Function and regulation of ULK1: From physiology to pathology | 10.1016/j.gene.2022.146772 | Review of literature |
| #69 | 2024 | Harnessing a triphenylphosphine-based AIE nano-platform for triggering incomplete mitophagy to continuously augment anti-tumor immune response in hepatocellular carcinoma | 10.1016/j.nantod.2023.102090 | Not focused on STING pathway mechanisms |
| #70 | 2014 | HBV life cycle is restricted in mouse hepatocytes expressing human NTCP | 10.1038/cmi.2013.66 | Not focused on STING pathway mechanisms |
| #71 | 2020 | Hemoperfusion plus continuous veno-venous hemofiltration in the treatment of patients with multiple organ failure after wasp stings | 10.1177/0391398819881459 | Not focused on STING pathway mechanisms |
| #72 | 2020 | Highly efficient and tumor-selective nanoparticles for dual-targeted immunogene therapy against cancer | 10.1126/sciadv.aax5032 | Not focused on STING pathway mechanisms |
| #73 | 2021 | Human Cancer Cells Sense Cytosolic Nucleic Acids Through the RIG-I–MAVS Pathway and cGAS–STING Pathway | 10.3389/fcell.2020.606001 | Unrelated to liver disease |
| #74 | 1968 | Hunter's dermatoses; [Les dermatoses du chasseur.] |  | Not focused on STING pathway mechanisms |
| #75 | 2023 | Immune Checkpoint Inhibitors in pMMR/MSS Colorectal Cancer | 10.1007/s12029-023-00927-2 | Not focused on STING pathway mechanisms |
| #76 | 2021 | Inflammation and tumor progression: signaling pathways and targeted intervention | 10.1038/s41392-021-00658-5 | Not focused on STING pathway mechanisms |
| #77 | 2014 | Insect bite-like reaction in a patient with chronic lymphocytic leukemia | 10.1111/ddg.12326 | Not focused on STING pathway mechanisms |
| #78 | 2023 | Injectable hydrogel loaded with lysed OK-432 and doxorubicin for residual liver cancer after incomplete radiofrequency ablation | 10.1186/s12951-023-02170-0 | Not focused on STING pathway mechanisms |
| #79 | 2017 | Investigation of phosphorylated adjuvants co-encapsulated with a model cancer peptide antigen for the treatment of colorectal cancer and liver metastasis | 10.1016/j.vaccine.2017.03.067 | Not focused on STING pathway mechanisms |
| #80 | 2023 | Letter to the editor: Disrupted BRCA1-PALB2 interaction induces tumor immunosuppression and T-lymphocyte infiltration in HCC through cGAS-STING pathway | 10.1002/hep.32690 | Secondary literature or commentary |
| #81 | 2024 | LicochalconeB inhibits cGAS-STING signaling pathway and prevents autoimmunity diseases | 10.1016/j.intimp.2024.111550 | Unrelated to liver disease |
| #82 | 2018 | Lipotoxicity induces hepatic protein inclusions through TANK binding kinase 1–mediated p62/sequestosome 1 phosphorylation | 10.1002/hep.29742 | Not focused on STING pathway mechanisms |
| #83 | 2023 | Mitochondrial DNA-triggered innate immune response: mechanisms and diseases | 10.1038/s41423-023-01086-x | Review of literature |
| #84 | 2021 | Mitophagy in tumorigenesis and metastasis | 10.1007/s00018-021-03774-1 | Review of literature |
| #85 | 2021 | Molecular and radiological features of microsatellite stable colorectal cancer cases with dramatic responses to immunotherapy | 10.21873/anticanres.15080 | Review of literature |
| #86 | 2023 | Monocytes reprogrammed by tumor microparticle vaccine inhibit tumorigenesis and tumor development | 10.1186/s12645-023-00190-x | Not focused on STING pathway mechanisms |
| #87 | 2024 | Nanomaterial-encapsulated STING agonists for immune modulation in cancer therapy | 10.1186/s40364-023-00551-z | Unrelated to liver disease |
| #88 | 2021 | Nanoparticle delivery improves the pharmacokinetic properties of cyclic dinucleotide STING agonists to open a therapeutic window for intravenous administration | 10.1016/j.jconrel.2020.11.017 | Unrelated to liver disease |
| #89 | 2022 | Neoantigen Immunotherapeutic-Gel Combined with TIM-3 Blockade Effectively Restrains Orthotopic Hepatocellular Carcinoma Progression | 10.1021/acs.nanolett.1c04977 | Not focused on STING pathway mechanisms |
| #90 | 2023 | P62, a multifunctional regulator in chronic liver diseases: a review | 10.3867/j.issn.1000-3002.2023.02.009 | Review of literature |
| #91 | 2022 | PARP inhibitor plus radiotherapy reshapes an inflamed tumor microenvironment that sensitizes small cell lung cancer to the anti-PD-1 immunotherapy | 10.1016/j.canlet.2022.215852 | Unrelated to liver disease |
| #92 | 2023 | Peptide nanotube loaded with a STING agonist, c-di-GMP, enhance cancer immunotherapy against melanoma | 10.1007/s12274-022-5102-z | Unrelated to liver disease |
| #93 | 2021 | Platinum-based chemotherapy in combination with PD-1/PD-L1 inhibitors: preclinical and clinical studies and mechanism of action | 10.1080/17425247.2021.1825376 | Not focused on STING pathway mechanisms |
| #94 | 2023 | Potentiating the Systemic Immunity by Bacteria-Delivered Sting Activation in a Tumor Microenvironment | 10.1002/adfm.202307001 | Not focused on STING pathway mechanisms |
| #95 | 2023 | PRMT1 mediated methylation of cGAS suppresses anti-tumor immunity | 10.1038/s41467-023-38443-3 | Unrelated to liver disease |
| #96 | 2023 | Remodeling of Tumor Microenvironment by Nanozyme Combined cGAS–STING Signaling Pathway Agonist for Enhancing Cancer Immunotherapy | 10.3390/ijms241813935 | Unrelated to liver disease |
| #97 | 2023 | Response to 'Hyperbaric oxygen facilitates teniposide-induced cGAS-STING activation to enhance the antitumor efficacy of PD-1 antibody in HCC' by Yang et al | 10.1136/jitc-2022-006648 | Secondary literature or commentary |
| #98 | 2014 | Review of alternanthera sessilis with reference to traditional siddha medicine |  | Review of literature |
| #99 | 2022 | Role of the cGAS–STING pathway in systemic and organ-specific diseases | 10.1038/s41581-022-00589-6 | Review of literature |
| #100 | 2022 | SGLT2 inhibitor activates the STING/IRF3/IFN-β pathway and induces immune infiltration in osteosarcoma | 10.1038/s41419-022-04980-w | Unrelated to liver disease |
| #101 | 2022 | STING agonist enhances the efficacy of programmed death-ligand 1 monoclonal antibody in breast cancer immunotherapy by activating the interferon-β signalling pathway | 10.1080/15384101.2022.2029996 | Unrelated to liver disease |
| #102 | 2017 | STING signaling in tumorigenesis and cancer therapy: A friend or foe? | 10.1016/j.canlet.2017.05.026 | Review of literature |
| #103 | 2021 | STING-dependent induction of lipid peroxidation mediates intestinal ischemia-reperfusion injury | 10.1016/j.freeradbiomed.2020.12.010 | Unrelated to liver disease |
| #104 | 2021 | Systems Biology to Understand and Regulate Human Retroviral Proinflammatory Response | 10.3389/fimmu.2021.736349 | Not focused on STING pathway mechanisms |
| #105 | 2022 | Systemic benefit of radiation therapy via abscopal effect | 10.3389/fonc.2022.987142 | Review of literature |
| #106 | 2024 | Targeting AXL induces tumor-intrinsic immunogenic response in tyrosine kinase inhibitor-resistant liver cancer | 10.1038/s41419-024-06493-0 | Not focused on STING pathway mechanisms |
| #107 | 2021 | Targeting innate immunity in cancer therapy | 10.3390/vaccines9020138 | Review of literature |
| #108 | 2023 | Targeting STING in cancer: Challenges and emerging opportunities | 10.1016/j.bbcan.2023.188983 | Review of literature |
| #109 | 2021 | The abscopal effect of radiation therapy | 10.2217/fon-2020-0994 | Review of literature |
| #110 | 2021 | The Alternatively Spliced Isoforms of Key Molecules in the cGAS-STING Signaling Pathway | 10.3389/fimmu.2021.771744 | Unrelated to liver disease |
| #111 | 2023 | The battle between the innate immune cGAS-STING signaling pathway and human herpesvirus infection | 10.3389/fimmu.2023.1235590 | Unrelated to liver disease |
| #112 | 2020 | The cGAS-STING pathway is a therapeutic target in a preclinical model of hepatocellular carcinoma | 10.1038/s41388-019-1108-8 | Review of literature |
| #113 | 2021 | The cGAS–STING pathway as a therapeutic target in inflammatory diseases | 10.1038/s41577-021-00524-z | Review of literature |
| #114 | 2021 | The complex role of AIM2 in autoimmune diseases and cancers | 10.1002/iid3.443 | Not focused on STING pathway mechanisms |
| #115 | 2021 | The Cytosolic DNA-Sensing cGAS-STING Pathway in Liver Diseases | 10.3389/fcell.2021.717610 | Review of literature |
| #116 | 2019 | The genus Echinops: Phytochemistry and biological activities: A review | 10.3389/fphar.2019.01234 | Review of literature |
| #117 | 2019 | The innate antiviral response in animals: An evolutionary perspective from flagellates to humans | 10.3390/v11080758 | Not focused on STING pathway mechanisms |
| #118 | 2023 | Therapeutic Effects of ADU-S100 as STING Agonist and CpG ODN1826 as TLR9 Agonist in CT-26 Model of Colon Carcinoma | 10.22067/ijvst.2023.80505.1223 | Unrelated to liver disease |
| #119 | 2021 | THERAPEUTIC POTENTIAL OF SCORPION VENOM IN CANCER TREATMENT AS ANTICANCER AGENT: A REVIEW | 10.5958/0974-4568.2021.00044.2 | Review of literature |
| #120 | 2023 | Tissue fibrosis induced by radiotherapy: current understanding of the molecular mechanisms, diagnosis and therapeutic advances | 10.1186/s12967-023-04554-0 | Review of literature |
| #121 | 2023 | Tumor Regression upon Intratumoral and Subcutaneous Dosing of the STING Agonist ALG-031048 in Mouse Efficacy Models | 10.3390/ijms242216274 | Unrelated to liver disease |
| #122 | 2022 | Type I interferon-mediated tumor immunity and its role in immunotherapy | 10.1007/s00018-022-04219-z | Not focused on STING pathway mechanisms |
| #123 | 2020 | Type I Interferons and Malaria: A Double-Edge Sword Against a Complex Parasitic Disease | 10.3389/fcimb.2020.594621 | Review of literature |
| #124 | 2014 | Visceral leishmaniasis-associated hemophagocytic lymphohistiocytosis in a traveler returning from a pilgrimage to the Camino de Santiago | 10.1111/jtm.12145 | Not focused on STING pathway mechanisms |
| #125 | 2019 | Vaccinia virus-mediated cancer immunotherapy: Cancer vaccines and oncolytics | 10.1186/s40425-018-0495-7 | Not focused on STING pathway mechanisms |
| #126 | 2022 | Zinc cyclic di-AMP nanoparticles target and suppress tumours via endothelial STING activation and tumour-associated macrophage reinvigoration | 10.1038/s41565-022-01225-x | Unrelated to liver disease |

| **Supplementary Table 11-4.** List of excluded studies in C-DILI. | | | | |
| --- | --- | --- | --- | --- |
| Item | Year | Title | DOI | Reason for exclusion |
| #1 | 2017 | A cell-based high throughput screening assay for the discovery of cGAS-STING pathway agonists | 10.1016/j.antiviral.2017.10.001 | Non-experimental or observational study |
| #2 | 2023 | A new NRF2 activator for the treatment of human metabolic dysfunction-associated fatty liver disease | 10.1016/j.jhepr.2023.100845 | Not focused on STING pathway mechanisms |
| #3 | 2020 | A Potential Role for Mitochondrial DNA in the Activation of Oxidative Stress and Inflammation in Liver Disease | 10.1155/2020/5835910 | Review of literature |
| #4 | 2023 | Activation of CD4 T cells during prime immunization determines the success of a therapeutic hepatitis B vaccine in HBV-carrier mouse models | 10.1016/j.jhep.2022.12.013 | Not focused on STING pathway mechanisms |
| #5 | 2017 | Acute pancreatitis due to cephalexin: A case report and review of literature | 10.1038/ajg.2017.311 | Review of literature |
| #6 | 2018 | Aetiology of anaphylaxis in patients referred to an immunology clinic in Colombo, Sri Lanka | 10.1186/s13223-018-0295-0 | Not focused on STING pathway mechanisms |
| #7 | 2018 | Assessment of pharmacology and toxicology of anti-CTLA-4 antibody (ADU-1604) in non-human primates and evaluation of local anti-CTLA-4 application | 10.1158/1538-7445.AM2018-1702 | Not focused on STING pathway mechanisms |
| #8 | 2013 | Cell type-specific subcellular localization of phospho- TBK1 in response to cytoplasmic viral DNA | 10.1371/journal.pone.0083639 | Not focused on STING pathway mechanisms |
| #9 | 2019 | CHAPTER 14: Synthetic Agonists of Toll-like Receptors and Therapeutic Applications | 10.1039/9781788015714-00306 | Review of literature |
| #10 | 2021 | Current status of intralesional agents in treatment of malignant melanoma | 10.21037/atm-21-491 | Not focused on STING pathway mechanisms |
| #11 | 2007 | Cutaneous hemorrhage or necrosis findings after Vespa mandarinia (wasp) stings may predict the occurrence of multiple organ injury: A case report and review of literature | 10.1080/15563650701664871 | Not focused on STING pathway mechanisms |
| #12 | 2019 | Cytosolic Nucleic Acid Sensors in Inflammatory and Autoimmune Disorders | 10.1016/bs.ircmb.2018.10.002 | Review of literature |
| #13 | 2018 | Development of a Validated Interferon Score Using NanoString Technology | 10.1089/jir.2017.0127 | Not focused on STING pathway mechanisms |
| #14 | 2023 | DUSP1 protects against ischemic acute kidney injury through stabilizing mtDNA via interaction with JNK | 10.1038/s41419-023-06247-4 | Unrelated to liver disease |
| #15 | 2024 | HCV-induced autophagy and innate immunity | 10.3389/fimmu.2024.1305157 | Not focused on STING pathway mechanisms |
| #16 | 2018 | HEPATIC GAP JUNCTIONS AMPLIFY ALCOHOL-INDUCED LIVER INJURY BY PROPAGATING CGAMP-MEDIATED IRF3 ACTIVATION | 10.1016/S0016-5085(18)33721-1 | Not focused on STING pathway mechanisms |
| #17 | 2022 | How SARS-CoV-2 dodges immune surveillance and facilitates infection: an analytical review | 10.1080/14787210.2022.2078307 | Review of literature |
| #18 | 2014 | Innate immune response induced by baculovirus attenuates transgene expression in mammalian cells | 10.1128/JVI.03055-13 | Unrelated to liver disease |
| #19 | 2014 | Innate signaling in the inflammatory immune disorders | 10.1016/j.cytogfr.2014.06.003 | Review of literature |
| #20 | 2017 | Investigation of intracellular innate immune responses to AAV gene therapy in the retina |  | Not focused on STING pathway mechanisms |
| #21 | 2018 | Liver immune cells release type 1 interferon due to DNA sensing and amplify liver injury from acetaminophen overdose | 10.3390/cells7080088 | Not focused on STING pathway mechanisms |
| #22 | 2022 | Macrophage-Specific Deletion of Cytosolic Nucleotide Sensors Ameliorate Kidney Fibrosis and Inflammation |  | Unrelated to liver disease |
| #23 | 2020 | Molecular Underpinnings of Severe Coronavirus Disease 2019 | 10.1001/jama.2020.14015 | Review of literature |
| #24 | 2023 | Non-small cell lung cancers (NSCLCs) oncolysis using coxsackievirus B5 and synergistic DNA-damage response inhibitors | 10.1038/s41392-023-01603-4 | Not focused on STING pathway mechanisms |
| #25 | 2023 | Normalization of hepatic ChREBP activity does not protect against liver disease progression in a mouse model for Glycogen Storage Disease type Ia | 10.1186/s40170-023-00305-3 | Not focused on STING pathway mechanisms |
| #26 | 2020 | Nucleic Acid Sensors as Therapeutic Targets for Human Disease | 10.1016/j.immuni.2020.04.004 | Non-experimental or observational study |
| #27 | 2021 | PCV2 targets cGAS to inhibit type I interferon induction to promote other DNA virus infection | 10.1371/journal.ppat.1009940 | Unrelated to liver disease |
| #28 | 2023 | Protective role of cGAS in NASH is related to the maintenance of intestinal homeostasis | 10.1111/liv.15610 | Unrelated to liver disease |
| #29 | 2023 | PTEN-induced kinase 1 is associated with renal aging, via the cGAS-STING pathway | 10.1111/acel.13865 | Unrelated to liver disease |
| #30 | 2023 | Senotherapeutics: An emerging approach to the treatment of viral infectious diseases in the elderly | 10.3389/fcimb.2023.1098712 | Review of literature |
| #31 | 2019 | STING activation reprograms tumor vasculatures and synergizes with VEGFR2 blockade | 10.1172/JCI125413 | Unrelated to liver disease |
| #32 | 2018 | STING signaling: A key to therapeutic tumor immunity | 10.2217/imt-2018-0064 | Review of literature |
| #33 | 2017 | Superior immunogenicity of HCV envelope glycoproteins when adjuvanted with cyclic-di-AMP, a STING activator or archaeosomes | 10.1016/j.vaccine.2017.10.072 | Not focused on STING pathway mechanisms |
| #34 | 2019 | Suppression of complex protumorigenic phenotypes in chronic injury-associated hepatocarcinogenesis is dependent on IL-6/STAT3 signaling | 10.1016/S0618-8278(19)30052-0 | Not focused on STING pathway mechanisms |
| #35 | 2019 | Targeting DNA damage response promotes antitumor immunity through STING-mediated T-cell activation in small cell lung cancer | 10.1158/2159-8290.CD-18-1020 | Unrelated to liver disease |
| #36 | 2021 | The adjuvanticity of manganese for microbial vaccines via activating the IRF5 signaling pathway | 10.1016/j.bcp.2021.114720 | Unrelated to liver disease |
| #37 | 2022 | The Evolutionary Dance between Innate Host Antiviral Pathways and SARS-CoV-2 | 10.3390/pathogens11050538 | Review of literature |
| #38 | 2021 | The inhibitory receptor TIM-3 limits activation of the cGAS-STING pathway in intra-tumoral dendritic cells by suppressing extracellular DNA uptake | 10.1016/j.immuni.2021.04.019 | Unrelated to liver disease |
| #39 | 2014 | Traditional uses, phytochemistry and pharmacology of Ficus carica: A review | 10.3109/13880209.2014.892515 | Review of literature |
| #40 | 2019 | The Role of Nucleic Acid Sensing in Controlling Microbial and Autoimmune Disorders | 10.1016/bs.ircmb.2018.08.002 | Review of literature |
| #41 | 2014 | Traditional uses, phytochemistry and pharmacology of Ficus carica: A review | 10.3109/13880209.2014.892515 | Review of literature |
| #42 | 2017 | Transmissible Gastroenteritis Virus Papain-Like Protease 1 Antagonizes Production of Interferon- β through Its Deubiquitinase Activity | 10.1155/2017/7089091 | Not focused on STING pathway mechanisms |
| #43 | 2017 | Unusual onset of adult still’s disease due to a systemic reaction to artificial breast implants | 10.1007/s00393-017-0277-5 | A case report |
| #44 | 2022 | Urolithin A Attenuates Hyperuricemic Nephropathy in Fructose-Fed Mice by Impairing STING-NLRP3 Axis-Mediated Inflammatory Response via Restoration of Parkin-Dependent Mitophagy | 10.3389/fphar.2022.907209 | Unrelated to liver disease |
| #45 | 2017 | Wasp venom–induced acute kidney injury: a serious health hazard | 10.1016/j.kint.2017.05.035 | Unrelated to liver disease |
| #46 | 2023 | Yi-Shen-Xie-Zhuo formula alleviates cisplatin-induced AKI by regulating inflammation and apoptosis via the cGAS/STING pathway | 10.1016/j.jep.2023.116327 | Review of literature |
| #47 | 2023 | A new NRF2 activator for the treatment of human metabolic dysfunction-associated fatty liver disease | 10.1016/j.jhepr.2023.100845 | Not focused on STING pathway mechanisms |
| #48 | 2010 | A rare case of severe intoxication from multiple bee stings with a favorable outcome. |  | Not focused on STING pathway mechanisms |
| #49 | 2020 | A Potential Role for Mitochondrial DNA in the Activation of Oxidative Stress and Inflammation in Liver Disease | 10.1155/2020/5835910 | Not focused on STING pathway mechanisms |
| #50 | 2023 | Activation of CD4 T cells during prime immunization determines the success of a therapeutic hepatitis B vaccine in HBV-carrier mouse models | 10.1016/j.jhep.2022.12.013 | Not focused on STING pathway mechanisms |
| #51 | 2016 | Acute interstitial nephritis, toxic hepatitis and toxic myocarditis following multiple Asian giant hornet stings in Shaanxi Province, China | 10.1007/s12199-016-0516-4 | Not focused on STING pathway mechanisms |
| #52 | 2013 | Acute Lonomia obliqua caterpillar envenomation-induced physiopathological alterations in rats: Evidence of new toxic venom activities and the efficacy of serum therapy to counteract systemic tissue damage | 10.1016/j.toxicon.2013.08.061 | Not focused on STING pathway mechanisms |
| #53 | 2010 | Acute physiopathological effects of honeybee (Apis mellifera) envenoming by subcutaneous route in a mouse model | 10.1016/j.toxicon.2010.07.005 | Not focused on STING pathway mechanisms |
| #54 | 2007 | Acute renal insufficiency and toxic hepatitis following scorpions sting. |  | Not focused on STING pathway mechanisms |
| #55 | 1988 | Acute systemic toxic reactions caused by Hornet stings |  | Not focused on STING pathway mechanisms |
| #56 | 2021 | Aedes aegypti mosquito saliva ameliorates acetaminophen-induced liver injury in mice | 10.1371/journal.pone.0245788 | Not focused on STING pathway mechanisms |
| #57 | 2018 | Aetiology of anaphylaxis in patients referred to an immunology clinic in Colombo, Sri Lanka | 10.1186/s13223-018-0295-0 | Not focused on STING pathway mechanisms |
| #58 | 2020 | An updated review on Couroupita guianensis Aubl: A sacred plant of India with myriad medicinal properties | 10.15171/JHP.2020.01 | Not focused on STING pathway mechanisms |
| #59 | 2014 | Anaphylactic shock caused by sting of crown-of-thorns starfish (Acanthaster planci) | 10.1016/j.forsciint.2014.01.001 | Not focused on STING pathway mechanisms |
| #60 | 2010 | Asthma that is unresponsive to usual care | 10.1503/cmaj.090089 | Not focused on STING pathway mechanisms |
| #61 | 2021 | Balasubramide derivative 3C attenuates atherosclerosis in apolipoprotein E-deficient mice: role of AMPK-STAT1-STING signaling pathway | 10.18632/aging.202929 | Unrelated to liver disease |
| #62 | 2018 | Bee venom therapy: Potential mechanisms and therapeutic applications | 10.1016/j.toxicon.2018.04.012 | Not focused on STING pathway mechanisms |
| #63 | 2010 | Beneficial effects of green tea: A literature review | 10.1186/1749-8546-5-13 | Review of literature |
| #64 | 2013 | Cell type-specific subcellular localization of phospho- TBK1 in response to cytoplasmic viral DNA | 10.1371/journal.pone.0083639 | Not focused on STING pathway mechanisms |
| #65 | 2019 | cGAMP inhibits tumor growth in colorectal cancer metastasis through the STING/STAT3 axis in a zebrafish xenograft model | 10.1016/j.fsi.2019.09.075 | Unrelated to liver disease |
| #66 | 2020 | cGAS/STING Pathway Activation Contributes to Delayed Neurodegeneration in Neonatal Hypoxia-Ischemia Rat Model: Possible Involvement of LINE-1 | 10.1007/s12035-020-01904-7 | Unrelated to liver disease |
| #67 | 2019 | CHAPTER 14: Synthetic Agonists of Toll-like Receptors and Therapeutic Applications | 10.1039/9781788015714-00306 | Review of literature |
| #68 | 2018 | Combined Proteome and Toxicology Approach Reveals the Lethality of Venom Toxins from Jellyfish Cyanea nozakii | 10.1021/acs.jproteome.8b00568 | Not focused on STING pathway mechanisms |
| #69 | 2022 | Curcumol Suppresses CCF-Mediated Hepatocyte Senescence Through Blocking LC3B–Lamin B1 Interaction in Alcoholic Fatty Liver Disease | 10.3389/fphar.2022.912825 | Not focused on STING pathway mechanisms |
| #70 | 2021 | CRIg+ Macrophages Prevent Gut Microbial DNA–Containing Extracellular Vesicle–Induced Tissue Inflammation and Insulin Resistance | 10.1053/j.gastro.2020.10.042 | Not focused on STING pathway mechanisms |
| #71 | 2017 | Cyclic GMP-AMP Ameliorates Diet-induced Metabolic Dysregulation and Regulates Proinflammatory Responses Distinctly from STING Activation | 10.1038/s41598-017-05884-y | Unrelated to liver disease |
| #72 | 2021 | Cyclic GMP-AMP promotes the acute phase response and protects against Escherichia coli infection in mice | 10.1016/j.bcp.2021.114541 | Unrelated to liver disease |
| #73 | 2019 | Cytosolic Nucleic Acid Sensors in Inflammatory and Autoimmune Disorders | 10.1016/bs.ircmb.2018.10.002 | Review of literature |
| #74 | 2020 | Dengue virus induces PCSK9 expression to alter antiviral responses and disease outcomes | 10.1172/JCI137536 | Not focused on STING pathway mechanisms |
| #75 | 2018 | Development of a Validated Interferon Score Using NanoString Technology | 10.1089/jir.2017.0127 | Not focused on STING pathway mechanisms |
| #76 | 2022 | Discovery of fusidic acid derivatives as novel STING inhibitors for treatment of sepsis | 10.1016/j.ejmech.2022.114814 | Unrelated to liver disease |
| #77 | 2021 | DNA sensing and associated type 1 interferon signaling contributes to progression of radiation-induced liver injury | 10.1038/s41423-020-0395-x | Not focused on STING pathway mechanisms |
| #78 | 2023 | DUSP1 protects against ischemic acute kidney injury through stabilizing mtDNA via interaction with JNK | 10.1038/s41419-023-06247-4 | Not focused on STING pathway mechanisms |
| #79 | 2015 | Effect of cytokine antibodies in the immunomodulation of inflammatory response and metabolic disorders induced by scorpion venom | 10.1016/j.intimp.2015.05.002 | Not focused on STING pathway mechanisms |
| #80 | 2016 | Efficacy of Two Combinations of Blood Purification Techniques for the Treatment of Multiple Organ Failure Induced by Wasp Stings | 10.1159/000442740 | Not focused on STING pathway mechanisms |
| #81 | 2016 | Endoplasmic reticulum stress-induced hepatocellular death pathways mediate liver injury and fibrosis via stimulator of interferon genes | 10.1074/jbc.M116.736991 | Not focused on STING pathway mechanisms |
| #82 | 2022 | Engaging Pattern Recognition Receptors in Solid Tumors to Generate Systemic Antitumor Immunity | 10.1007/978-3-030-96376-7_3 | Review of literature |
| #83 | 2023 | Ethnomedicinal uses, phytochemistry, pharmacological activities and toxicological effects of Mimosa pudica- A review | 10.1016/j.prmcm.2023.100241 | Review of literature |
| #84 | 2023 | Flavonoid derivative DMXAA attenuates cisplatin-induced acute kidney injury independent of STING signaling | 10.1042/CS20220728 | Unrelated to liver disease |
| #85 | 2024 | Formation of hepatocyte cytoplasmic inclusions and their contribution to methylcarbamate-induced hepatocarcinogenesis in F344 rats | 10.1093/toxsci/kfad131 | Unrelated to liver disease |
| #86 | 2022 | GRP75 Regulates Mitochondrial-Supercomplex Turnover to Modulate Insulin Sensitivity | 10.2337/db21-0173 | Not focused on STING pathway mechanisms |
| #87 | 2024 | HCV-induced autophagy and innate immunity | 10.3389/fimmu.2024.1305157 | Review of literature |
| #88 | 2005 | Hornet sting-induced toxic hepatitis | 10.1081/CLT-200050386 | Review of literature |
| #89 | 2005 | Hospital management of animal and human bites | 10.1016/j.jhin.2005.02.007 | Review of literature |
| #90 | 2021 | How the innate immune DNA sensing cGAS–STING pathway is involved in autophagy | 10.3390/ijms222413232 | Review of literature |
| #91 | 2017 | Hyperbaric oxygen therapy in the treatment of acute intoxications |  | Review of literature |
| #92 | 2023 | IL-33/ST2 antagonizes STING signal transduction via autophagy in response to acetaminophen-mediated toxicological immunity | 10.1186/s12964-023-01114-3 | Unrelated to liver disease |
| #93 | 2007 | Immunizations, neonatal hyperbilirubinemia and animal-induced injuries | 10.1097/MOP.0b013e32823a3c77 | Review of literature |
| #94 | 2019 | Induction of humoral and cellular immune response to HBV vaccine can be up-regulated by STING ligand | 10.1016/j.virol.2019.03.013 | Non-experimental or observational study |
| #95 | 2023 | Injectable hydrogel loaded with lysed OK-432 and doxorubicin for residual liver cancer after incomplete radiofrequency ablation | 10.1186/s12951-023-02170-0 | Not focused on STING pathway mechanisms |
| #96 | 2014 | Innate signaling in the inflammatory immune disorders | 10.1016/j.cytogfr.2014.06.003 | Review of literature |
| #97 | 2018 | JAK1/2 inhibition with baricitinib in the treatment of autoinflammatory interferonopathies |  | Not focused on STING pathway mechanisms |
| #98 | 2022 | Lethal Waterhouse–Friderichsen syndrome caused by Capnocytophaga canimorsus in an asplenic patient | 10.1186/s12879-022-07590-1 | A case report |
| #99 | 2024 | LicochalconeB inhibits cGAS-STING signaling pathway and prevents autoimmunity diseases | 10.1016/j.intimp.2024.111550 | Unrelated to liver disease |
| #100 | 2012 | Malaria Prevention in Travelers | 10.1016/j.idc.2012.05.003 | Review of literature |
| #101 | 2024 | Manganese-based microcrystals equipped with Ythdf1-targeted biomimetic nanovaccines for reinforced dendritic cell spatiotemporal orchestration | 10.1016/j.nantod.2023.102112 | Not focused on STING pathway mechanisms |
| #102 | 2022 | Mitochondrial cristae architecture protects against mtDNA release and inflammation | 10.1016/j.celrep.2022.111774 | Not focused on STING pathway mechanisms |
| #103 | 2023 | Mitochondrial DNA-triggered innate immune response: mechanisms and diseases | 10.1038/s41423-023-01086-x | Review of literature |
| #104 | 2023 | Mitochondrial oxidative stress regulates LonP1-TDP-43 pathway and rises mitochondrial damage in carbon tetrachloride-induced liver fibrosis | 10.1016/j.ecoenv.2023.115409 | Not focused on STING pathway mechanisms |
| #105 | 2021 | Mitophagy in tumorigenesis and metastasis | 10.1007/s00018-021-03774-1 | Review of literature |
| #106 | 2022 | Molecular and functional characterization of zinc ﬁnger aspartate-histidine-histidine-cysteine (DHHC)-type containing 1, ZDHHC1 in Chinese perch Siniperca chuatsi | 10.1016/j.fsi.2022.09.023 | Not focused on STING pathway mechanisms |
| #107 | 2020 | mtDNA-STING pathway promotes necroptosis-dependent enterocyte injury in intestinal ischemia reperfusion | 10.1038/s41419-020-03239-6 | Unrelated to liver disease |
| #108 | 2018 | Multiple bee stings, multiple organs involved: A case report | 10.1590/0037-8682-0341-2017 | A case report |
| #109 | 2022 | Multiple Wasp Stings Leading to Rhabdomyolysis Induced Acute Kidney Injury with Incidental Ectopic Kidney: A Case Report | 10.31729/jnma.7866 | A case report |
| #110 | 2023 | Nanodroplet-enhanced sonodynamic therapy potentiates immune checkpoint blockade for systemic suppression of triple-negative breast cancer | 10.1016/j.actbio.2022.12.023 | Unrelated to liver disease |
| #111 | 2017 | NLRX1 promotes immediate IRF1-directed antiviral responses by limiting dsRNA-activated translational inhibition mediated by PKR | 10.1038/ni.3853 | Not focused on STING pathway mechanisms |
| #112 | 2010 | Patients Presenting with Acute Toxin Ingestion | 10.1016/j.anclin.2010.01.002 | Not focused on STING pathway mechanisms |
| #113 | 2021 | PCV2 targets cGAS to inhibit type I interferon induction to promote other DNA virus infection | 10.1371/journal.ppat.1009940 | Unrelated to liver disease |
| #114 | 2014 | Pelagia noctiluca (Scyphozoa) crude venom injection elicits oxidative stress and inflammatory response in rats | 10.3390/md12042182 | Not focused on STING pathway mechanisms |
| #115 | 2006 | Problem pathogens: Prevention of malaria in travellers | 10.1016/S1473-3099(06)70410-8 | Review of literature |
| #116 | 2015 | Protective effects of batimastat against hemorrhagic injuries in delayed jellyfish envenomation syndrome models |  | Not focused on STING pathway mechanisms |
| #117 | 2020 | Role of Interferon-γ–Producing Th1 Cells in a Murine Model of Type I Interferon–Independent Autoinflammation Resulting From DNase II Deficiency | 10.1002/art.41090 | Not focused on STING pathway mechanisms |
| #118 | 2022 | Role of Mitochondrial Nucleic Acid Sensing Pathways in Health and Patho-Physiology | 10.3389/fcell.2022.796066 | Review of literature |
| #119 | 2023 | Senotherapeutics: An emerging approach to the treatment of viral infectious diseases in the elderly | 10.3389/fcimb.2023.1098712 | Review of literature |
| #120 | 2023 | Stimulator of interferon genes from Asian swamp eel (MaSTING) is involved in host defense against bacterial infection | 10.1016/j.fsi.2023.108788 | Unrelated to liver disease |
| #121 | 2019 | STING activation reprograms tumor vasculatures and synergizes with VEGFR2 blockade | 10.1172/JCI125413 | Unrelated to liver disease |
| #122 | 2015 | Sting agonists induce an innate antiviral immune response against hepatitis B virus | 10.1128/AAC.04321-14 | Not focused on STING pathway mechanisms |
| #123 | 2018 | STING signaling: A key to therapeutic tumor immunity | 10.2217/imt-2018-0064 | Review of literature |
| #124 | 2005 | Survival after treatment of rabies with induction of coma | 10.1056/NEJMoa050382 | Unrelated to liver disease |
| #125 | 2017 | Superior immunogenicity of HCV envelope glycoproteins when adjuvanted with cyclic-di-AMP, a STING activator or archaeosomes | 10.1016/j.vaccine.2017.10.072 | Non-experimental or observational study |
| #126 | 2005 | Survival after treatment of rabies with induction of coma | 10.1056/NEJMoa050382 | Unrelated to liver disease |
| #127 | 2019 | Targeting DNA damage response promotes antitumor immunity through STING-mediated T-cell activation in small cell lung cancer | 10.1158/2159-8290.CD-18-1020 | Unrelated to liver disease |
| #128 | 2021 | The adjuvanticity of manganese for microbial vaccines via activating the IRF5 signaling pathway | 10.1016/j.bcp.2021.114720 | Not focused on STING pathway mechanisms |
| #129 | 2019 | The cGAS-cGAMP-STING pathway: A molecular link between immunity and metabolism | 10.2337/dbi18-0052 | Review of literature |
| #130 | 2021 | The cGAS–STING pathway: more than fighting against viruses and cancer | 10.1186/s13578-021-00724-z | Review of literature |
| #131 | 2000 | The diagnosis and management of urticaria: A practice parameter |  | Review of literature |
| #132 | 2022 | The Evolutionary Dance between Innate Host Antiviral Pathways and SARS-CoV-2 | 10.3390/pathogens11050538 | Review of literature |
| #133 | 2019 | The Role of Nucleic Acid Sensing in Controlling Microbial and Autoimmune Disorders | 10.1016/bs.ircmb.2018.08.002 | Book chapter |
| #134 | 2023 | Therapeutic Effects of ADU-S100 as STING Agonist and CpG ODN1826 as TLR9 Agonist in CT-26 Model of Colon Carcinoma | 10.22067/ijvst.2023.80505.1223 | Unrelated to liver disease |
| #135 | 2019 | Thymoquinone ameliorates Pachycondyla sennaarensis venom-induced acute toxic shock in male rats | 10.1186/s40360-019-0375-x | Not focused on STING pathway mechanisms |
| #136 | 2023 | Tissue fibrosis induced by radiotherapy: current understanding of the molecular mechanisms, diagnosis and therapeutic advances | 10.1186/s12967-023-04554-0 | Review of literature |
| #137 | 2014 | Traditional uses, phytochemistry and pharmacology of Ficus carica: A review | 10.3109/13880209.2014.892515 | Review of literature |
| #138 | 2017 | Transmissible Gastroenteritis Virus Papain-Like Protease 1 Antagonizes Production of Interferon- β through Its Deubiquitinase Activity | 10.1155/2017/7089091 | Unrelated to liver disease |
| #139 | 2017 | Unusual onset of adult still’s disease due to a systemic reaction to artificial breast implants; | 10.1007/s00393-017-0277-5 | Unrelated to liver disease |
| #140 | 2022 | Urolithin A Attenuates Hyperuricemic Nephropathy in Fructose-Fed Mice by Impairing STING-NLRP3 Axis-Mediated Inflammatory Response via Restoration of Parkin-Dependent Mitophagy | 10.3389/fphar.2022.907209 | Unrelated to liver disease |
| #141 | 2019 | Vaccinia virus-mediated cancer immunotherapy: Cancer vaccines and oncolytics | 10.1186/s40425-018-0495-7 | Review of literature |
| #142 | 2023 | Yi-Shen-Xie-Zhuo formula alleviates cisplatin-induced AKI by regulating inflammation and apoptosis via the cGAS/STING pathway | 10.1016/j.jep.2023.116327 | Unrelated to liver disease |

| **Supplementary Table 11-5.** List of excluded studies in Liver Cirrhosis. | | | | |
| --- | --- | --- | --- | --- |
| Item | Year | Title | DOI | Reason for exclusion |
| #1 | 2014 | 97% sustained virologic response in Japanese patients with chronic genotype 2 hepatitis C virus infection receiving sofosbuvir in combination with ribavirin for 12 weeks: Results from a phase 3 multicenter study | 10.1002/hep.27516 | Not focused on STING pathway mechanism |
| #2 | 2023 | A HETEROGENEOUS SUBTYPE OF BILIARY EPITHELIAL SENESCENCE MAY BE INVOLVED IN THE PATHOGENESIS OF PRIMARY BILIARY CHOLANGITIS | 10.1097/HEP.0000000000000580 | Unrelated to liver disease |
| #3 | 2019 | A new hepatoma cell line exhibiting high susceptibility to hepatitis B virus infection | 10.1016/j.bbrc.2019.05.126 | Not focused on STING pathway mechanism |
| #4 | 2023 | A new NRF2 activator for the treatment of human metabolic dysfunction-associated fatty liver disease | 10.1016/j.jhepr.2023.100845 | Not focused on STING pathway mechanism |
| #5 | 2023 | A new nuclear-erythroid-2-related factor 2 activator for the treatment of non-alcoholic steatohepatitis: evidence of metabolic and anti-fibroinflammatory effects in human precision cut liver slices | 10.1016/S0168-8278(23)00435-X | Not focused on STING pathway mechanism |
| #6 | 2022 | A non-canonical cGAS-STING-PERK pathway may be involved in the pathogenesis of liver fibrosis | 10.1111/sji.13210 | Review of literature |
| #7 | 2010 | Asthma that is unresponsive to usual care | 10.1503/cmaj.090089 | Not focused on STING pathway mechanism |
| #8 | 2015 | Avian mite dermatitis: An Italian case indicating the establishment and spread of Ornithonyssus bursa (Acari: Gamasida: Macronyssidae) (Berlese, 1888) in Europe | 10.1111/ijd.12739 | A case report |
| #9 | 2018 | Can innate immune system targets turn up the heat on 'cold' tumours? | 10.1038/nrd.2017.264 | Review of literature |
| #10 | 2022 | Can the cGAS-STING Pathway Play a Role in the Dry Eye? | 10.3389/fimmu.2022.929230 | Unrelated to liver disease |
| #11 | 2011 | Cardiovascular collapse following pulmonary thromboembolism during orthotopic liver transplantation | 10.1002/lt.22457 | Unrelated to liver disease |
| #12 | 2023 | CHOLANGIOCYTEMEDIATED INFILTRATION OF NEUTROPHILS IN THE PERI-PORTAL REGION INDUCES OXIDATIVE STRESS IN PRIMARY SCLEROSING CHOLANGITIS | 10.1097/HEP.0000000000000580 | Unrelated to liver disease |
| #13 | 2021 | Circulatory endothelin 1-regulating rnas panel: Promising biomarkers for non-invasive nafld/nash diagnosis and stratification: Clinical and molecular pilot study | 10.3390/genes12111813 | Non-experimental or observational study |
| #14 | 2014 | Clinical audit of the patients: A retrospective study in a govt medical college, Hassan | 10.5958/j.0976-5506.5.2.080 | Non-experimental or observational study |
| #15 | 2019 | Delayed Admission and Management of Pediatric Acute Kidney Injury and Multiple Organ Dysfunction Syndrome in Children with Multiple Wasp Stings: A Case Series | 10.1159/000504043 | A case report |
| #16 | 2010 | Diagnostic protocol of pruritus | 10.1016/S0304-5412(10)70007-X | Review of literature |
| #17 | 2017 | Disease-associated mutations identify a novel region in human STING necessary for the control of type I interferon signaling | 10.1016/j.jaci.2016.10.031 | Non-experimental or observational study |
| #18 | 2023 | Editorial: Viruses, innate immunity, and antiviral strategies: from basic research to clinical applications | 10.3389/fcimb.2023.1268363 | Secondary literature or commentary |
| #19 | 2018 | Edwardsiella tarda bacteremia in untreated hepatitis C: A fatal case report | 10.1002/jac5.1059 | A case report |
| #20 | 2023 | Effect of the STING pathway on inflammatory cytokine secretion and phagocytosis by peripheral blood monocytes in patients with hepatitis B cirrhosis | 10.3969/j.issn.1001-5256.2023.09.013 | Non-experimental or observational study |
| #21 | 2020 | Emerging views of mitophagy in immunity and autoimmune diseases | 10.1080/15548627.2019.1603547 | Review of literature |
| #22 | 2022 | Expanding role of deoxyribonucleic acid-sensing mechanism in the development of lifestyle-related diseases | 10.3389/fcvm.2022.881181 | Review of literature |
| #23 | 2021 | Family case of SAVI-syndrome in the practice of a rheumatologist and pulmonologist | 10.1186/s12969-021-00632-z | A case report |
| #24 | 2016 | Foreca sting lifetime health outcomes and costs of treatment for non-alcoholic fatty liver disease |  | Non-experimental or observational study |
| #25 | 2024 | HCV-induced autophagy and innate immunity | 10.3389/fimmu.2024.1305157 | Review of literature |
| #26 | 2008 | Hepatic fibrosis influences the growth of hepatocellular carcinoma |  | Review of literature |
| #27 | 2021 | Hepatic stellate cell senescence in liver fibrosis: Characteristics, mechanisms and perspectives | 10.1016/j.mad.2021.111572 | Review of literature |
| #28 | 2014 | Innate signaling in the inflammatory immune disorders | 10.1016/j.cytogfr.2014.06.003 | Review of literature |
| #29 | 2007 | Medico ethno botany; a study on the Amhara ethnic group of Gondar district of North Gondar zone Ethiopia |  | Not focused on STING pathway mechanism |
| #30 | 2022 | Multiplexed Digital Spatial Protein Profiling Reveals Distinct Phenotypes of Mononuclear Phagocytes in Livers with Advanced Fibrosis | 10.3390/cells11213387 | Non-experimental or observational study |
| #31 | 2022 | Multiplexed digital spatial protein profiling reveals distinct phenotypes of portal mononuclear phagocytes in livers with advanced fibrosis | 10.1007/s12072-022-10337-4 | Non-experimental or observational study |
| #32 | 2020 | Non-small-cell lung cancer signaling pathways, metabolism, and PD-1/PD-L1 antibodies | 10.3390/cancers12061475 | Unrelated to liver disease |
| #33 | 2023 | Novel role of macrophage Foxo1-mediated YAP-Notch axis in NASH progression | 10.1016/S0168-8278(23)02126-8 | Not focused on STING pathway mechanism |
| #34 | 2010 | Orchids: A review of uses in traditional medicine, its phytochemistry and pharmacology |  | Review of literature |
| #35 | 2023 | P62, a multifunctional regulator in chronic liver diseases: a review |  | Review of literature |
| #36 | 2018 | Predictors of mortality in acute kidney injury patients admitted to medicine intensive care unit in a Rural Tertiary Care Hospital | 10.4103/ijccm.IJCCM_462_17 | Non-experimental or observational study |
| #37 | 2015 | Regulating against the dysregulation: New treatment options in autoinflammation | 10.1007/s00281-015-0501-9 | Review of literature |
| #38 | 2020 | Role of Interferon-γ–Producing Th1 Cells in a Murine Model of Type I Interferon–Independent Autoinflammation Resulting From DNase II Deficiency | 10.1002/art.41090 | Not focused on STING pathway mechanism |
| #39 | 2021 | Sars-cov-2 induces strong inflammation in organoids derived directly from cirrhotic nash patient liver but not healthy donor liver. | 10.1002/hep.32187 | Not focused on STING pathway mechanism |
| #40 | 2016 | Scorpion envenoming caused by Tityus cf. silvestris evolving with severe muscle spasms in the Brazilian Amazon | 10.1016/j.toxicon.2016.06.015 | Not focused on STING pathway mechanism |
| #41 | 2021 | STING and liver disease | 10.1007/s00535-021-01803-1 | Review of literature |
| #42 | 2021 | Sting deficiency fails to protect mice against nonalcoholic steatohepatitis induced by a fructosepalmitate- cholesterol diet. | 10.1002/hep.32188 | Not focused on STING pathway mechanism |
| #43 | 2019 | Sting expression in monocyte-derived macrophages is associated with the progression of liver inflammation and fibrosis in patients with nonalcoholic fatty liver disease |  | Non-experimental or observational study |
| #44 | 2020 | STING expression in monocyte-derived macrophages is associated with the progression of liver inflammation and fibrosis in patients with nonalcoholic fatty liver disease | 10.1038/s41374-019-0342-6 | Non-experimental or observational study |
| #45 | 2023 | STING mediates hepatocyte pyroptosis in liver fibrosis by Epigenetically activating the NLRP3 inflammasome | 10.1016/j.redox.2023.102691 | Non-experimental or observational study |
| #46 | 2015 | STING-associated vasculopathy with onset in infancy: New clinical findings and mutation in three Turkish children | 10.1159/000381120 | A case report |
| #47 | 2009 | Successful surgical resection for peritoneal implantation of hepatocellular carcinoma at the paracardial portion | 10.1155/2009/231854 | Non-experimental or observational study |
| #48 | 2019 | Suppression of complex protumorigenic phenotypes in chronic injury-associated hepatocarcinogenesis is dependent on IL-6/STAT3 signaling | 10.1016/S0618-8278(19)30052-0 | Not focused on STING pathway mechanism |
| #49 | 2023 | TAK1 deficiency promotes liver injury and tumorigenesis via ferroptosis and macrophage cGAS-STING signaling | 10.1016/S0168-8278(23)00435-X | Secondary literature or commentary |
| #50 | 2023 | The anti-HIV drug Rilpivirine downregulates migration and proliferation of activated hepatic stellate cells: relevance for the purpose of drug repurposing in liver fibrosis | 10.1016/S0168-8278(23)00957-1 | Not focused on STING pathway mechanism |
| #51 | 2023 | The battle between the innate immune cGAS-STING signaling pathway and human herpesvirus infection | 10.3389/fimmu.2023.1235590 | Review of literature |
| #52 | 2022 | The cGAS–STING signaling in cardiovascular and metabolic diseases: Future novel target option for pharmacotherapy | 10.1016/j.apsb.2021.05.011 | Unrelated to liver disease |
| #53 | 2021 | The Cytosolic DNA-Sensing cGAS-STING Pathway in Liver Diseases | 10.3389/fcell.2021.717610 | Review of literature |
| #54 | 2022 | The effect of the cyclic GMP-AMP synthase-stimulator of interferon genes signaling pathway on organ inflammatory injury and fibrosis | 10.3389/fphar.2022.1033982 | Review of literature |
| #55 | 2022 | Therapeutic Advances in Viral Hepatitis A–E | 10.1007/s12325-022-02070-z | Review of literature |
| #56 | 2023 | Tissue fibrosis induced by radiotherapy: current understanding of the molecular mechanisms, diagnosis and therapeutic advances | 10.1186/s12967-023-04554-0 | Review of literature |
| #57 | 2022 | UBC13-MEDIATED P62 UBIQUITINATION PROTECTS AGAINST NONALCOHOLIC STEATOHEPATITIS LINKED TO DEFECT IN MITOPHAGY | 10.1016/S0016-5085(22)62682-9 | Not focused on STING pathway mechanism |
| #58 | 2021 | Vibrio vulnificus infection attributed to bee sting: a case report | 10.1080/22221751.2021.1977589 | A case report |

| **Supplementary Table 11-6.** List of excluded studies in HIRI. | | | | |
| --- | --- | --- | --- | --- |
| Item | Year | Title | DOI | Reason for exclusion |
| #1 | 2020 | Aging aggravated liver ischemia and reperfusion injury by promoting STING-mediated NLRP3 activation in macrophages | 10.1111/acel.13186 | Secondary literature or commentary |
| #2 | 2024 | Blockade of Hepatocyte PCSK9 Ameliorates Hepatic Ischemia-Reperfusion Injury by Promoting Pink1-Parkin–Mediated Mitophagy | 10.1016/j.jcmgh.2023.09.004 | Not focused on STING pathway mechanism |
| #3 | 2019 | Mitochondrial DNA in liver inflammation and oxidative stress | 10.1016/j.lfs.2019.05.020 | Review of literature |
| #4 | 2023 | Stimulator of interferon genes (STING): Key therapeutic targets in ischemia/reperfusion injury | 10.1016/j.biopha.2023.115458 | Review of literature |
| #5 | 2021 | The cGAS-STING Pathway: Novel Perspectives in Liver Diseases | 10.3389/fimmu.2021.682736 | Review of literature |
| #6 | 2021 | The Cytosolic DNA-Sensing cGAS-STING Pathway in Liver Diseases | 10.3389/fcell.2021.717610 | Review of literature |
| #7 | 2024 | The dual function of cGAS-STING signaling axis in liver diseases | 10.1038/s41401-023-01220-5 | Review of literature |

| **Supplementary Table 11-7.** List of excluded studies in Parasitic Liver Disease. | | | | | |
| --- | --- | --- | --- | --- | --- |
| Item | | Year | Title | DOI | Reason for exclusion |
| #1 | 2020 | | Type I Interferons and Malaria: A Double-Edge Sword Against a Complex Parasitic Disease | 10.3389/fcimb.2020.594621 | Review of literature |
| #2 | 2007 | | A new view of malaria provided by parasite imaging | 10.1016/S0001-4079(19)32945-0 | Review of literature |
| #3 | 2017 | | A novel stimulator of interferon gene (STING) from Larimichthys crocea and their involvement in immune response to ectoparasite Cryptocaryon irritans infection | 10.1016/j.fsi.2017.09.056 | Non-experimental or observational study |
| #4 | 2020 | | An Activation-Clearance Model for Plasmodium vivax Malaria | 10.1007/s11538-020-00706-1 | Review of literature |
| #5 | 2002 | | Cellular and molecular requirements for the recall of IL-4-producing memory CD4(+)CD45RO(+)CD27(-) T cells during protection induced by attenuated Plasmodium falciparum sporozoites. | 10.1002/1521-4141(200203)32:3<652::AID-IMMU652>3.0.CO;2-9 | Not focused on STING pathway mechanism |
| #6 | 2021 | | Clustering and Erratic Movement Patterns of Syringe-Injected versus Mosquito-Inoculated Malaria Sporozoites Underlie Decreased Infectivity | 10.1128/mSphere.00218-21 | Not focused on STING pathway mechanism |
| #7 | 2016 | | Effect of bee venom or proplis on molecular and parasitological aspects of Schistosoma mansoni infected mice. | 10.1007/s12639-014-0516-5 | Not focused on STING pathway mechanism |
| #8 | 1970 | | Entomology of the gastrointestinal tract: a brief review. | 10.1093/milmed/135.3.165 | Review of literature |
| #9 | 1977 | | Exotic diseases. Ounce of prevention or pound of cure? | 10.1080/00325481.1977.11714584 | Review of literature |
| #10 | 2007 | | Experimental transmission of Hepatozoon gracilis (Wenyon, 1909) com. nov., in its natural host the bean skink lizard (Mabuya quinquetaeniata quinquetaeniata) and vector Culex (C.) pipiens (Diptera: Culicidae). | 10.1080/00325481.1977.11714585 | Not focused on STING pathway mechanism |
| #11 | 1990 | | Experimental transmission of Leishmania infantum by the bite of Phlebotomus perniciosus from Switzerland. | 10.1016/0001-706X(90)90011-N | Not focused on STING pathway mechanism |
| #12 | 2012 | | Extrahepatic Exoerythrocytic Forms of Rodent Malaria Parasites at the Site of Inoculation: Clearance after Immunization, Susceptibility to Primaquine, and Contribution to Blood-Stage Infection | 10.1128/IAI.00246-12 | Not focused on STING pathway mechanism |
| #13 | 2023 | | High endemicity of <i>Opisthorchis viverrini</i> infection among people in northern Cambodia confirmed by adult worm expulsion | 10.1038/s41598-023-36544-z | Not focused on STING pathway mechanism |
| #14 | 2008 | | Immunity to a salivary protein of a sand fly vector protects against the fatal outcome of visceral leishmaniasis in a hamster model | 10.1073/pnas.0712153105 | Not focused on STING pathway mechanism |
| #15 | 2020 | | IMRAS-A clinical trial of mosquito-bite immunization with live, radiation-attenuated <i>P. falciparum</i> sporozoites: Impact of immunization parameters on protective efficacy and generation of a repository of immunologic reagents | 10.1371/journal.pone.0233840 | Not focused on STING pathway mechanism |
| #16 | 2021 | | IMRAS-Immunization with radiation-attenuated <i>Plasmodium falciparum</i> sporozoites by mosquito bite: Cellular immunity to sporozoites, CSP, AMA1, TRAP and CelTOS | 10.1371/journal.pone.0256396 | Not focused on STING pathway mechanism |
| #17 | 2011 | | Induction of Antimalaria Immunity by Pyrimethamine Prophylaxis during Exposure to Sporozoites Is Curtailed by Parasite Resistance | 10.1128/AAC.01717-10 | Not focused on STING pathway mechanism |
| #18 | 2015 | | Laser mimicking mosquito bites for skin delivery of malaria sporozoite vaccines | 10.1016/j.jconrel.2015.02.031 | Not focused on STING pathway mechanism |
| #19 | 1962 | | Man to man transfer of two strains of Plasmodium cynomolgi by mosquito bite. | 10.4269/ajtmh.1962.11.186 | Not focused on STING pathway mechanism |
| #20 | 2014 | | Multi-Strain Infections and 'Relapse' of <i>Leucocytozoon sabrazesi</i> Gametocytes in Domestic Chickens in Southern China | 10.1371/journal.pone.0094877 | Not focused on STING pathway mechanism |
| #21 | 2010 | | Multiple hymenoptera stings in children: clinical and laboratory manifestations | 10.1007/s00431-010-1209-4 | Not focused on STING pathway mechanism |
| #22 | 2014 | | Outcome of acute East African trypanosomiasis in a Polish traveller treated with pentamidine | 10.1186/1471-2334-14-111 | Not focused on STING pathway mechanism |
| #23 | 1994 | | Predicting outcome in malaria: correlation between rate of exposure to infected mosquitoes and level of Plasmodium falciparum parasitemia. | 10.4269/ajtmh.1994.51.523 | Not focused on STING pathway mechanism |
| #24 | 2011 | | Protective immunity against malaria by 'natural immunization': a question of dose, parasite diversity, or both? | 10.1016/j.coi.2011.05.009 | Not focused on STING pathway mechanism |
| #25 | 2000 | | Queensland tick typhus infection acquired whilst on holiday in Queensland. |  | Not focused on STING pathway mechanism |
| #26 | 2006 | | Re-ingestion of <i>Plasmodium berghei</i> sporozoites after delivery into the host by mosquitoes | 10.4269/ajtmh.2006.75.1200 | Not focused on STING pathway mechanism |
| #27 | 2014 | | Safety and Comparability of Controlled Human <i>Plasmodium falciparum</i> Infection by Mosquito Bite in Malaria-Naive Subjects at a New Facility for Sporozoite Challenge | 10.1371/journal.pone.0109654 | Not focused on STING pathway mechanism |
| #28 | 1990 | | [Schistosoma species in Senegal with special reference to the biology, epidemiology and pathology of Schistosoma curassoni Brumpt, 1931]. |  | Review of literature |
| #29 | 2020 | | STING-Dependent Pathways Restraining Severe Schistosome Immunopathology |  | Review of literature |
| #30 | 1972 | | The itching patient. A review of the causes of pruritus. | 10.1177/003693307201701005 | Review of literature |
| #31 | 2020 | | The role of the adaptor molecule STING during <i>Schistosoma mansoni</i> infection | 10.1038/s41598-020-64788-6 | Review of literature |
| #32 | 2011 | | Therapeutic orchids: traditional uses and recent advances - An overview | 10.1016/j.fitote.2010.09.007 | Review of literature |
| #33 | 2022 | | Toxic Systemic Reaction after Bee Stings in a Bitch | 10.22456/1679-9216.121867 | Not focused on STING pathway mechanism |
| #34 | 2012 | | Tropical Diseases Definition, Geographic Distribution, Transmission, and Classification | 10.1016/j.idc.2012.02.007 | Review of literature |
| #35 | 2018 | | TRSP is dispensable for the <i>Plasmodium</i> pre-erythrocytic phase | 10.1038/s41598-018-33398-8 | Not focused on STING pathway mechanism |
| #36 | 2007 | | Uninfected mosquito bites confer protection against infection with malaria parasites | 10.1128/IAI.01928-06 | Review of literature |
| #37 | 1994 | | Visceral leishmaniasis in Teresina, State of Piaui, Brazil: preliminary observations on the detection and transmissibility of canine and sandfly infections. | 10.1590/S0074-02761994000200001 | Review of literature |
| #38 | 2023 | | Whole sporozoite immunization with <i>Plasmodium falciparum</i> strain NF135 in a randomized trial |  | Not focused on STING pathway mechanism |

| **Supplementary Table 12.** List of secondary exclusion. | | | | | |
| --- | --- | --- | --- | --- | --- |
| Item | Year | Title | Disease | DOI | Reason for exclusion |
| #1 | 2019 | KNOCKDOWN OF STIMULATOR OF INTERFERON GENES (STING) REDUCES BILIARY SENESCENCE AND LIVER INFLAMMATION AND FIBROSIS IN THE MDR2-/- MOUSE MODEL OF PRIMARY SCLEROSING CHOLANGITIS (PSC) | NFALD/ALD | 10.1016/S0016-5085(19)40070-X | Secondary literature or commentary |
| #2 | 2020 | mTDNA released by cholangiocytes promotes sting activation and fibrotic reaction | NFALD/ALD | 10.1002/hep.31579 | Meeting Abstract |
| #3 | 2022 | MSC EXOSOMES ALLEVIATE NONALCOHOLIC STEATOHEPATITIS BY REGULATING MACROPHAGE POLARIZATION THROUGH THE STING SIGNALING PATHWAY | NFALD/ALD | 10.1002/hep.32697 | Meeting Abstract |
| #4 | 2023 | MYELOID STING DISRUPTION ALLEVIATES THE DELETERIOUS EFFECT OF MYELOID CELL-SPECIFIC PFKFB3 DEFICIENCY ON DIET-INDUCED NAFLD IN MICE | NFALD/ALD | 10.1016/S0016-5085(23)03937-9 | Meeting Abstract |
| #5 | 2023 | Notch1 inhibits the mechanistic role of STING signaling to regulate hepatocyte lipophagy in nonalcoholic steatohepatitis | NFALD/ALD | 10.3760/cma.j.cn501113-20230208-00042 | Non-English |
| #6 | 2021 | Sting deficiency fails to protect mice against nonalcoholic steatohepatitis induced by a fructosepalmitate- cholesterol diet. | NFALD/ALD | 10.1002/hep.32188 | Meeting Abstract |
| #7 | 2023 | TGF-β induced mitochondrial DNA release activates the hepatic stellate cells via sting-IRF3 pathway | NFALD/ALD | 10.1111/acer.5071 | Meeting Abstract |
| #8 | 2023 | TXNIP contributes to NAFLD and related fibrosis by activating the cGAS-STING-PERK pathway | NFALD/ALD | 10.1007/s00125-023-05969-6 | Meeting Abstract |
| #9 | 2022 | UBC13-MEDIATED P62 UBIQUITINATION PROTECTS AGAINST NONALCOHOLIC STEATOHEPATITIS LINKED TO DEFECT IN MITOPHAGY | NFALD/ALD | 10.1016/S0016-5085(22)62682-9 | Meeting Abstract |
| #10 | 2017 | A cell-based high throughput screening assay for the discovery of cGAS-STING pathway agonists | Hepatitis | 10.1016/j.antiviral.2017.10.001 | Not focusing on hepatitis |
| #11 | 2019 | AdrA as a potential immunomodulatory candidate for STING-mediated anti-viral therapy | Hepatitis | 10.1089/hum.2019.29095.abstracts | Meeting Abstract |
| #12 | 2023 | Enhanced anti-HBV function by dendritic cells through activation of the STING pathway | Hepatitis | 10.1007/s12072-023-10501-4 | Meeting Abstract |
| #13 | 2020 | mTDNA released by cholangiocytes promotes sting activation and fibrotic reaction | Hepatitis | 10.1002/hep.31579 | Meeting Abstract |
| #14 | 2021 | Sting agonist mediates antiviral effects in aav-hbv mouse model | Hepatitis | 10.1002/hep.32188 | Meeting Abstract |
| #15 | 2019 | Study on the relationship between cgas and AIM2 in HBV induced hepatocyte innate immune pathway | Hepatitis |  | Meeting Abstract |
| #16 | 2016 | The cGAS-sting pathway exhibits antiviral activity against hepatitis B virus infection | Hepatitis |  | Meeting Abstract |
| #17 | 2015 | STING deficiency protects from acetaminophen-induced acute liver failure in mice | Liver injury | 10.1002/hep.28195 | Meeting Abstract |
| #18 | 2020 | MELATONIN MODULATES STIMULATOR OF INTERFERON GENES (STING) ACTIVATION IN THE MDR2-/-MOUSE MODEL OF PRIMARY SCLEROSING CHOLANGITIS (PSC) | Liver Cirrhosis | 10.1016/S0016-5085(20)33811-7 | Meeting Abstract |
| #19 | 2020 | CROSSTALK SIGNALING BETWEEN MAST CELLS, HISTAMINE AND STIMULATOR OF INTERFERON GENE (STING/TMEM173) PROMOTES CHOLANGIOCARCINOMA (CCA) TUMORIGENESIS | Liver Cirrhosis | 10.1016/S0016-5085(20)33881-6 | Meeting Abstract |
| #20 | 2021 | Activation of cGAS/STING pathway upon TDP-43-mediated mitochondrial injury may be involved in the pathogenesis of liver fibrosis | Liver Cirrhosis | 10.1111/liv.14895 | Letter |
| #21 | 2019 | Downregulation of stimulator of interferon genes (STING) decreases biliary senescence and liver fibrosis in cholestatic mice through a decrease expression of TmiR24-3p/melatonin/aanat/PER1 signaling | Liver Cirrhosis |  | Meeting Abstract |
| #22 | 2023 | Hexafluoropropylene oxide trimer acid causes fibrosis in mice liver via mitochondrial ROS/cGAS-STING/NLRP3-mediated pyroptosis | Liver Cirrhosis | 10.1016/j.fct.2023.113706 | Meeting Abstract |
| #23 | 2013 | Innate immune signaling couples endoplasmic reticulum stress to hepatocyte apoptosis via STING and mitochondrial IRF3 interactions | Liver Cirrhosis | 10.1002/hep.26808 | Meeting Abstract |
| #24 | 2014 | Liver fibrogenesis is controlled by innate immune activation pathways in hepatocyte apoptosis | Liver Cirrhosis | 10.1002/hep.27518 | Meeting Abstract |
| #25 | 2022 | MITOCHONDRIAL DYNAMICS AND STASIS ARE CRITICAL FOR HEPATOCYTE FUNCTION AND LIVER TUMORIGENESIS | Liver Cirrhosis | 10.1002/hep.32697 | Meeting Abstract |
| #26 | 2020 | mTDNA released by cholangiocytes promotes sting activation and fibrotic reaction | Liver Cirrhosis | 10.1002/hep.31579 | Meeting Abstract |
| #27 | 2023 | TGF-β MEDIATED HSC TRANSDIFFERENTIATION REQUIRES RELEASE OF MITOCHONDRIAL DNA AND ACTIVATION OF THE CYTOSOLIC CGASSTING-IRF3 PATHWAY | Liver Cirrhosis | 10.1016/S0016-5085(23)01273-8 | Meeting Abstract |
| #28 | 2022 | Adoptive Transfer of Mesenchymal Stem Cells Activates Hedgehog/SMO/Gli1 Signaling and Inhibits STING-Mediated Innate Immune Response in Liver Transplant Inflammatory Injury. | HIRI | 10.1111/ajt.17073 | Meeting Abstract |
| #29 | 2022 | TXNIP-MEDIATED CYLD/NRF2/OASL1 SIGNALING IS ESSENTIAL FOR CONTROLLING STING/TBK1 FUNCTION AND CELL DEATH PATHWAY IN LIVER INFLAMMATORY INJURY | HIRI | 10.1002/hep.32697 | Meeting Abstract |
| #30 | 2022 | AS2O3 DRIVES MACROPHAGE TO INDUCE ANTITUMOR IMMUNITY IN HEPATOCELLULAR CARCINOMA THROUGH THE UPREGULATION OF CGAS-STING SIGNALING | Liver Neoplasms | 10.1136/gutjnl-2022-IDDF.16 | Meeting Abstract |
| #31 | 2022 | Conjugation of Listeriolysin O to Anti-CD47 mAb: Harnessing Listeria Toxin to Weaponize Macrophages against Tumor Cells | Liver Neoplasms | 10.1016/j.ijrobp.2022.07.408 | Meeting Abstract |
| #32 | 2022 | First-in-human study of TAK-500, a novel STING agonist immune stimulating antibody conjugate (ISAC), alone and in combination with pembrolizumab in patients with select advanced solid tumors | Liver Neoplasms | 10.1158/1538-7445.AM2022-CT249 | Meeting Abstract |
| #33 | 2020 | Irradiation promotes hepatocellular carcinoma immune cloaking via PD-L1 upregulation induced by cGAS-STING activation | Liver Neoplasms | 10.1158/1538-7445.AM2020-6510 | Meeting Abstract |
| #34 | 2023 | Mitochondria-localized cGAS suppresses ferroptosis to promote cancer progression | Liver Neoplasms | 10.1038/s41422-023-00788-1 | The experimental results are only related to cGAS, not to the cGAS-STING pathway |
| #35 | 2022 | RECQL4 Remodels the Tumor Immune Microenvironment via the cGAS-STING Pathway in Hepatocellular Carcinoma | Liver Neoplasms | 10.1016/j.ijrobp.2022.07.2081 | Meeting Abstract |


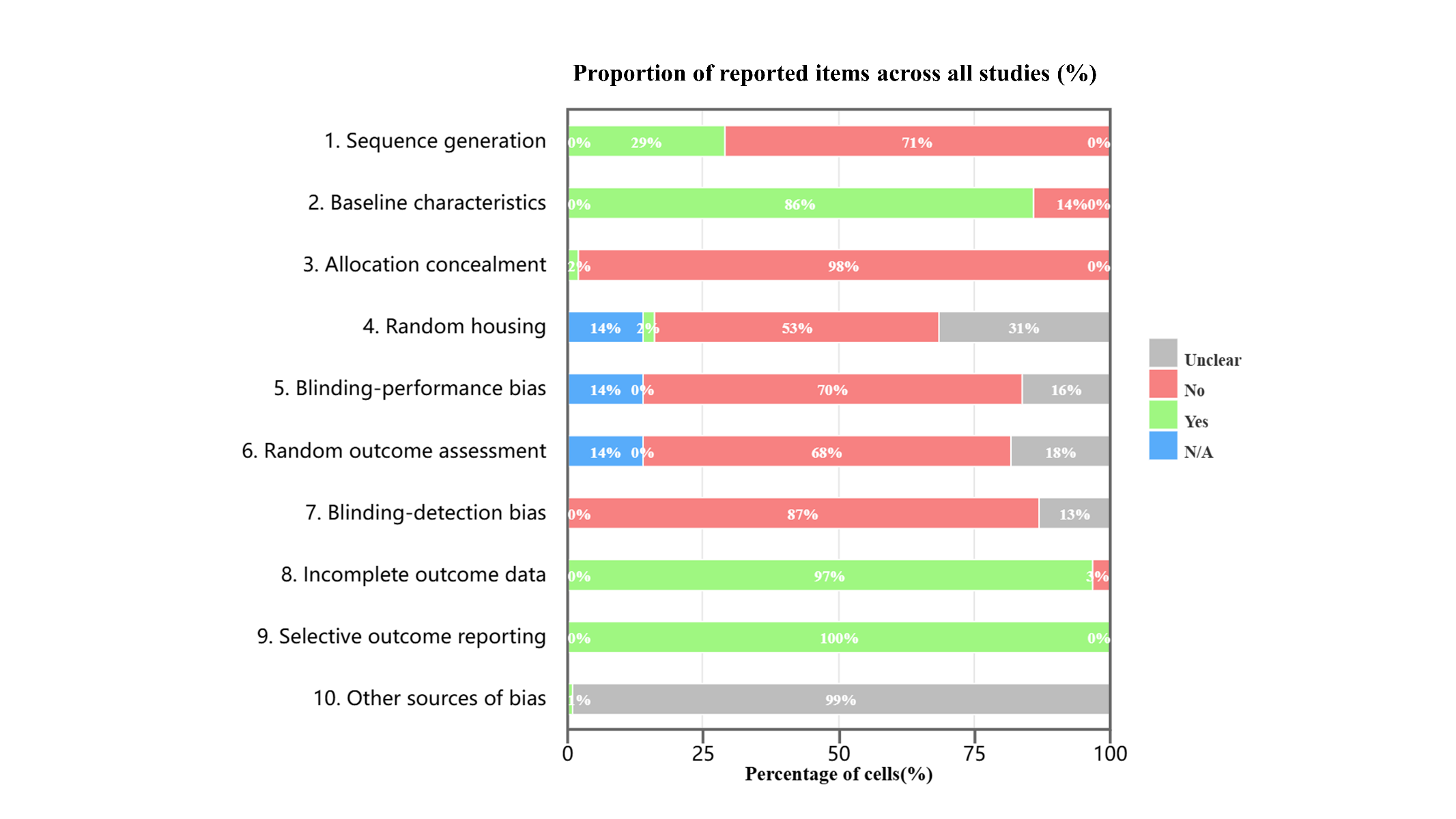


**Supplementary Fig 1.** Risk of bias trends across included studies using the SYRCLE tool.


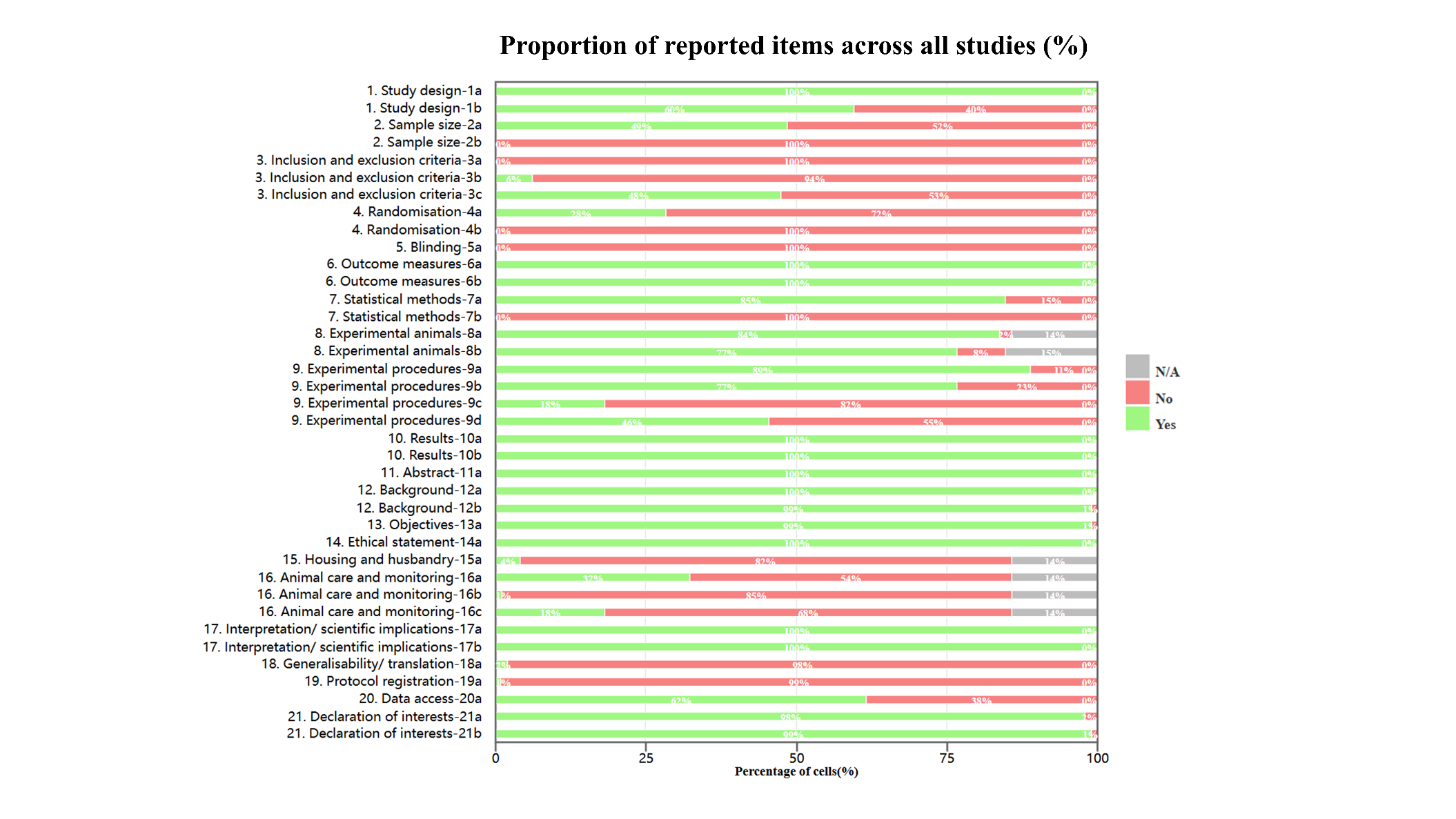


**Supplementary Fig 2.** ARRIVE 2.0 reporting quality assessment across included studies.

# Methods

**Search strategy**

The systematic literature review (SLR) followed the Preferred Reporting Items for Systematic Reviews and Meta-Analyses (PRISMA) guidelines **(Supplementary Tables 8-1–8-2)**. Eligible articles were identified through searches on PubMed, Embase, Scopus, and Web of Science up to March 29, 2024. Specific search strategies tailored to each database were employed **(****Supplementary Tables 9-1–9-7)**. The search terms included “cGAS-STING,” “Non-alcoholic Fatty Liver Disease,” “Liver Neoplasms,” “Hepatitis,” “Chemical and Drug-Induced Liver Injury,” “Liver Cirrhosis,” “Hepatic Reperfusion Injury,” and “Parasitic Liver Diseases,” among others. Two independent researchers (Y.C. and Z.H.) conducted the search, utilizing Medical Subject Headings (MeSH) terms when applicable.

**Study selection**

Following the publication and registration of the SLR protocol on PROSPERO(ID: CRD42024530171), two reviewers (Y.C. and Z.H.) independently screened all titles and abstracts. The screening process comprised two stages: the initial stage involved assessing titles and abstracts, while the second stage focused on reviewing the full texts of selected articles to determine their relevance for data extraction. In cases of disagreement between the reviewers during abstract and full-text screening, a third reviewer (Y.F.) resolved any discrepancies.

**Literature screening criteria**

Title and abstract screening were conducted to identify studies focusing on the cGAS-STING pathway in various liver diseases, including both animal and cellular experiments to elucidate the pathway's role in disease pathogenesis. Studies that did not meet the inclusion criteria—such as those lacking animal or cell experiments, reviews, case reports, or those not centered on the cGAS-STING pathway or liver diseases—were excluded. This review specifically included original peer-reviewed research articles that examined the expression of cGAS or STING in liver diseases of both animal and human origin. The PICO approach was employed to establish inclusion criteria for articles, as outlined in (**Supplementary Table 3**). For studies meeting the criteria for full-text review, data related to liver in vitro cell experiments, animal studies, genetic manipulation studies (e.g., gene knockouts, overexpression), pharmacological studies (e.g., utilizing cGAS-STING pathway agonists or inhibitors), mechanistic studies (e.g., investigating the molecular mechanisms of the STING pathway in liver diseases), solvent control (treatment involving a solvent that dissolved the drug or compound), gene knockout or knockdown control (where the control group may consist of wild-type cells or animals treated with gene editing vectors only), and normal control were extracted. The review primarily focused on key outcomes, including factors and expressions such as cGAS and STING, liver function indices like alanine aminotransferase (ALT) and aspartate aminotransferase (AST), lipid metabolism markers including TNF-α, IL-1β, lipid droplets, ROS, SOD, CAT, and caspase-3; fibrosis markers such as Col1a1, Col3a1, α-SMA, Fn, TGF-β, and GFAP; inflammatory response indicators like p-IRF3, IFN-β, p-p65/p65, IL6, IL1b, TNF-α, p-p62, IL10, and F4/80; and apoptosis markers such as Bax/Bcl2, C-Casp3, and cleaved PARP/PARP.

**Risk of bias assessment**

The quality of animal experiments was evaluated using SYRCLE’s Risk of Bias (RoB) tool for animal studies. This tool consists of ten items and related questions to assess experimental bias. Response options include “Yes” for studies meeting evaluation criteria with no bias, “No” for studies showing bias, and “Unclear” for studies not mentioning criteria, thus presenting an unclear risk of bias. The quality assessment was performed by Y.C. and reviewed by Z.H. and Y.F.

**Study reporting quality assessment**

The quality of the study was evaluated following the updated ARRIVE 2.0 guidelines. These criteria consist of 21 assessment points, divided into two categories: a core set of 10 elements and a supplementary set of 11 elements. The core set outlines the basic necessary components that must be incorporated in all animal research to allow for accurate evaluation of the findings by readers and reviewers. The supplementary set offers additional contextual information to enhance the study. In this analysis, the researchers transformed the ARRIVE 2.0 components into a series of 38 inquiries: 22 corresponding to the core elements and 16 associated with the supplementary set. Each study was evaluated with a response of “Yes,” “No,” or “Not Applicable (N/A)” for each inquiry.

**Data extraction**

Data extraction was independently performed by two reviewers (Y.C. and Y.F.). Data were obtained from published articles or extracted from figures and categorized according to outcome indicators. When examining the role of the cGAS-STING pathway in different liver diseases, we collected and categorized information such as article details (title, authors, publication year), animal models used (species, strain, treatment conditions), characteristics of cell lines (name, type, treatment conditions), and specific results (activation status of cGAS-STING pathway, key biological processes, cellular components, molecular functions, pathways involved). The comprehensive analysis encompassed the involvement of cGAS-STING in liver diseases, focusing on its mechanisms in inflammation, cell death, fibrosis, and tumorigenesis, as well as discussing current research advancements and the potential for therapeutic targeting. By systematically organizing and extracting these data, a thorough understanding of the cGAS-STING pathway’s role in liver diseases and its therapeutic possibilities was achieved. Due to the numerous outcome indicators and inconsistencies among studies, a meta-analysis was not conducted. Differences between conference abstracts and final papers were assessed, and final papers were included whenever possible.
